# Identification of a 6-RBP gene signature for a comprehensive analysis of glioma and ischemic stroke: Cognitive impairment and aging-related hypoxic stress

**DOI:** 10.3389/fnagi.2022.951197

**Published:** 2022-09-01

**Authors:** Weiwei Lin, Qiangwei Wang, Yisheng Chen, Ning Wang, Qingbin Ni, Chunhua Qi, Qian Wang, Yongjian Zhu

**Affiliations:** ^1^Department of Neurosurgery, The Second Affiliated Hospital of Zhejiang University School of Medicine, Hangzhou, China; ^2^Key Laboratory of Precise Treatment and Clinical Translational Research of Neurological Diseases of Zhejiang, Hangzhou, China; ^3^Department of Orthopedics, Shanghai General Hospital, Shanghai Jiao Tong University School of Medicine, Shanghai, China; ^4^Brain Center, Affiliated Zhejiang Hospital, Zhejiang University School of Medicine, Hangzhou, China; ^5^Postdoctoral Workstation, Department of Central Laboratory, The Affiliated Taian City Central Hospital of Qingdao University, Taian, China; ^6^College of Mathematical Medicine, Zhejiang Normal University, Jinhua, China

**Keywords:** elderly, glioma, ischemic stroke, RNA binding protein (RBP), dementia

## Abstract

There is mounting evidence that ischemic cerebral infarction contributes to vascular cognitive impairment and dementia in elderly. Ischemic stroke and glioma are two majorly fatal diseases worldwide, which promote each other's development based on some common underlying mechanisms. As a post-transcriptional regulatory protein, RNA-binding protein is important in the development of a tumor and ischemic stroke (IS). The purpose of this study was to search for a group of RNA-binding protein (RBP) gene markers related to the prognosis of glioma and the occurrence of IS, and elucidate their underlying mechanisms in glioma and IS. First, a 6-RBP (*POLR2F, DYNC1H1, SMAD9, TRIM21, BRCA1*, and *ERI1*) gene signature (RBPS) showing an independent overall survival prognostic prediction was identified using the transcriptome data from TCGA-glioma cohort (*n* = 677); following which, it was independently verified in the CGGA-glioma cohort (*n* = 970). A nomogram, including RBPS, 1p19q codeletion, radiotherapy, chemotherapy, grade, and age, was established to predict the overall survival of patients with glioma, convenient for further clinical transformation. In addition, an automatic machine learning classification model based on radiomics features from MRI was developed to stratify according to the RBPS risk. The RBPS was associated with immunosuppression, energy metabolism, and tumor growth of gliomas. Subsequently, the six RBP genes from blood samples showed good classification performance for IS diagnosis (AUC = 0.95, 95% CI: 0.902–0.997). The RBPS was associated with hypoxic responses, angiogenesis, and increased coagulation in IS. Upregulation of *SMAD9* was associated with dementia, while downregulation of *POLR2F* was associated with aging-related hypoxic stress. *Irf5*/*Trim21* in microglia and *Taf7*/*Trim21* in pericytes from the mouse cerebral cortex were identified as RBPS-related molecules in each cell type under hypoxic conditions. The RBPS is expected to serve as a novel biomarker for studying the common mechanisms underlying glioma and IS.

## Introduction

There is mounting evidence that ischemic cerebral infarction contributes to vascular cognitive impairment and dementia in elderly, but the origins of ischemic cerebral infarction are unclear. Understanding the vascular pathologies that cause ischemic cerebral infarction may yield strategies to prevent their occurrence and reduce their deleterious effects on brain function (Hartmann et al., 2018). Worldwide, ischemic stroke (IS) accounts for about 70% of all stroke events, with a proportion as high as 87% in the United States, and is also the second leading cause of death (Musuka et al., 2015; Benjamin et al., 2019; Phipps and Cronin, 2020). Glioma is a common type of invasive brain tumor and the leading cause of primary brain tumor-related deaths (Musuka et al., 2015; Velasco et al., 2019; Wang E. et al., 2019). Among them, glioblastoma multiforme (GBM; WHO IV) accounts for 45.2% of all primary malignant tumors of the central nervous system (CNS) and is one of the fatal tumor types, with a median survival time of fewer than 15 months (Ostrom et al., 2013; Bi et al., 2020; Wang et al., 2020).

Clinical studies show that glioma and cerebral ischemia can promote each other's occurrence during disease development and treatment (Fraum et al., 2011; Seidel et al., 2013; Wojtasiewicz et al., 2013; Farkas et al., 2018; Noda et al., 2022). Previous studies have reported that the incidence of brain tumors is higher in a cohort of patients with IS than in those without a history of IS (Qureshi et al., 2015; Chen et al., 2017; Tanislav et al., 2019). Similarly, stroke is a common complication among patients with tumor. A postmortem-based study reported that about 14.6% of non-CNS cancer cases showed cerebrovascular disease (CVD) (Graus et al., 1985). Moreover, embolic strokes are the most common cause of strokes in patients with cancer, possibly due to intravascular coagulopathy (Cestari et al., 2004); patients with active cancer show multiple infarcts (Kikuno et al., 2021). Gliomas account for 60% of ischemic strokes secondary to primary brain tumors, whereby complications due to surgery and radiotherapy form the majority (Kreisl et al., 2008). In these coexisting diseases, stroke is usually missed, often leading to increased neurological disabilities and injuries in susceptible individuals. Therefore, the pathogenesis of glioma could provide potential mechanisms for cerebral ischemia.

RNA-binding protein (RBP) is a large protein family, which plays a vital role in regulating gene expression through interactions with RNA. These proteins participate in many biological processes, such as splicing, lysis, and polyadenylation, as well as mRNA editing, localization, stabilization, and translation (Kedde et al., 2007; Liao et al., 2020; Van Nostrand et al., 2020). In addition, some studies suggest that the interaction between RBP and RNA plays a vital role in the occurrence and development of cancers (including renal cell carcinoma, triple-negative breast cancer, and lung squamous cell carcinoma) (Mohibi et al., 2019; Duan and Zhang, 2020; Kim et al., 2020; Li et al., 2020; Qin et al., 2020). In this context, many RBPs are reportedly associated with a poor prognosis of gliomas (Shao et al., 2013; Barbagallo et al., 2018; Lan et al., 2020). In IS, several RBPs participate in the development and influence the prognoses of these patients by promoting inflammatory reactions (Zhou et al., 2014; Sharma et al., 2021), increasing cerebrovascular permeability, promoting vasogenic cerebral edema (Ardelt et al., 2017), regulating apoptosis (Si et al., 2020; Zhang et al., 2020), and protecting neurons (Fang et al., 2021).

In the development of glioma and ischemic stroke events, some common pathways, such as hypoxia, brain inflammation, angiogenesis, and hypercoagulability, have been identified (Ghosh et al., 2019). Among them, hypoxia is the most widely accepted basis for building research models for studying the common mechanisms underlying glioma and IS (Søndergaard et al., 2002; Kasivisvanathan et al., 2011). However, the specific mechanism of co-occurrence or mutual promotion of glioma and ischemic stroke remains unclear. Many clinical studies have described this problem from a clinical perspective and expounded the possible common pathway underlying the pathogenesis from the perspective of each disease. Several RBP molecular or molecular combination markers are used to identify specific subgroups of patients with glioma, showing poor survival. Similarly, several RBPs are involved in the development of IS. However, there is a lack of a comprehensive analysis of the RBP family of genes in the common pathway underlying glioma and IS. Through an in-depth study on the role of RBPs, we hypothesized that RBP signature could not only provide an effective identification for molecular subtypes of patients with glioma with a poor prognosis but also yield certain reference values for the diagnosis of IS. Such biomarkers will also provide more reliable risk stratification and treatment targets for the clinicians to customize more accurate personalized treatment plans and ultimately improve the treatment efficacy.

Bioinformatics based on medical big data has solid advantages for analyzing the common molecular mechanisms and pathways for such coexisting diseases. In addition, the combination of radiomics and machine learning shows a good performance in image-based diagnosis or molecular subtype prediction and is more convenient for clinical application (Acs et al., 2020). The primary purpose of this study was to identify a group of RBP genes related to the prognosis of glioma and the occurrence of IS, and elucidate their mechanism in glioma, dementia, and IS. First, we identified a panel of RBP genes related to the prognosis and analyzed the pathogenesis of these genes in glioma. Next, using the radiomics features from MRI images, an automatic machine learning classifier was used to predict risk stratification based on this RBP gene signature in glioma. Finally, using bulk RNA sequencing (RNA-seq) and single-cell RNA sequencing (scRNA-seq) data, the classificatory performance and the potential mechanism of these RBP genes in IS were analyzed.

## Materials and methods

### Research design and data extraction

According to the research purpose, the study design was divided into two stages. The first stage involved the identification of a 6-RBP gene signature (RBPS) and functional analysis of the RBPS in glioma. The second stage was evaluating the expression and functions of the RBPS in IS and mouse cerebral cortex cells under hypoxic conditions.

The first stage could be subdivided into three steps as follows: the discovery and verification of biological gene markers and automatic machine learning prediction based on radiomics features. First, the standardized RNA expression profile data of 677 patients with glioma (including 698 tumor tissues and 5 adjacent normal tissue samples) were downloaded from TCGA (https://portal.gdc.cancer.gov/), and a 6-RBP gene signature related to the prognosis of glioma was identified. Next, the identified biomarkers were verified in independent clinical data sets using the transcriptome RNA expression profile data and clinical characteristics of patients with glioma (N = 970; Verification Cohort 1) in CGGA (https://www.cgga.org.cn/). Moreover, the clinical data of patients with Grade IV glioma in the GSE72951 data set (Erdem-Eraslan et al., 2016) (N = 110; Validation Cohort 2) and the gene expression profile data from gene chip analysis were obtained from the Gene Expression Omnibus (GEO) database for verification. Finally, using MRI-based radiomics features, an automatic machine learning classifier was constructed to predict the RBPS. MRI-based radiomics feature data from 132 patients with glioma were downloaded from TCIA (Clark et al., 2013) and used to train classifiers for predicting RBPS risk stratification (Bakas et al., 2017; Beers et al., 2018).

In the second stage, the possible mechanism underlying the six RBP genes in stroke and dementia was evaluated, and the gene regulatory network related to hypoxia was analyzed in mouse cerebral cortex cells. First, the GSE16561 dataset was retrieved from the GEO database to examine differentially expressed genes (DEGs) related to ischemic stroke. RNA-seq data in this dataset were derived from peripheral blood samples of 39 patients with ischemic stroke and 24 healthy controls (Barr et al., 2010; O'Connell et al., 2016, 2017). The GSE36980 dataset was used to explore DEGs associated with Alzheimer's disease, which included 33 patients with AD and 47 non-AD controls (Hokama et al., 2014). In addition, to study the expressions of the related genes at a single-cell level, RNA-seq data from 7,925 isolated mouse cerebral cortex cells were obtained from the GSE125708 dataset. In this data set, mice were divided into two groups: one group living in indoor air for 7 days was the normal oxygen concentration group, and the other group living in 7.5% oxygen concentration for 7 days was the hypoxia concentration group. Using this dataset, we examined the regulatory changes for the RBPS-related genes with changes in the oxygen concentration (Heng et al., 2019).

### Analysis of differentially expressed RBP genes

A total of 1,542 RNA-binding protein genes were collected from a published dataset (Gerstberger et al., 2014). Differentially expressed RBP genes were analyzed between tumor samples and normal samples adjacent to cancer in the TCGA-glioma dataset. An adjusted *p* value < 0.05 using the Benjamini-Hochberg false discovery rate (FDR) method (FDR < 0.05) and a logarithmic value of fold change >1 (|log_2_FC| > 1) were used as the cut-off criteria to screen differentially expressed RBP genes. Differentially expressed genes (DEGs) were used to perform Gene Ontology (GO) and Kyoto Encyclopedia of Genes and Genomes (KEGG) pathway enrichment analysis using the online DAVID database. The protein-protein interaction (PPI) analysis of DE-RBP genes was performed using STRING software (https://string-db.org/). Cytoscape software was used to build a sub-network to identify the PPI network's core DEGs. The “limma” (Ritchie et al., 2015) and “sva” (Leek et al., 2012) R packages were used to remove the batch effect for the gene expression data of the shared RBP genes in TCGA, CGGA, and GSE72951 datasets.

### Construction of a 6-RBP gene signature

To identify a clinically translatable RBP gene signature, the univariate Cox proportional hazard regression model and the Lasso penalty Cox regression model were used for evaluating the association of RBP genes in predicting overall survival (OS) in patients with glioma. Next, RBPS was constructed, and its value in predicting OS was evaluated. The risk score (RS) of the RBPS was calculated based on the linear combination of the gene expression (*EXP*_i_) multiplied by the corresponding coefficient (Coef_i_).


(1) $$\begin{array}{l} RS=\overset{n}{\underset{i=1}{\sum }}Coef_{i}\times EXP_{i} \end{array}$$

The median value of the gene signature risk score was used as a cut-off threshold to divide the entire patients with glioma into high- and low-risk groups. The Kaplan-Meier (K-M) method was used to plot survivor curves. The log-rank test was used to calculate the statistical difference between the two groups to evaluate the correlation of the RBPS with the OS outcome. Receiver operator characteristic (ROC) curve analysis of the RBPS with prognosis was performed using the “survivalROC” package (https://CRAN.R-project.org/package=survivalROC), and 95% confidence intervals (CI) of the area under the curve (AUC) were calculated by the “timeROC” (Blanche et al., 2013) package.

### Risk stratification of the RBPS

The expressions of the RBPS genes in samples were analyzed using the “pheatmap” package. The risk scores of RBPS were sorted from low to high to evaluate the relationship between the risk scores and patients' living status and overall survival time. Circosplot was drawn using the “RCircos” (Zhang et al., 2013) package to show the copy number variant status distribution of the RBPS genes and their position on chromosomes. To explore the relationship between the expression and copy number variant status of the RBPS genes, the differential expression analyses of RBPS genes among different copy number variants were performed to explore the role of a gene copy number variant in RBPS genes expression.

### Gene set enrichment analysis

Gene set enrichment analysis (GSEA) is a bioinformatics algorithm used to identify the differential expression of biological pathways between two biological states (Subramanian et al., 2005). GSEA was used to identify the pathway related to the RBPS. The “c2.cp.kegg.v7.1.symbols.gmt[Curated]” gene set collection from the Molecular Signatures Database (MSigDB) was used as a reference for enrichment analysis (Subramanian et al., 2005; Liberzon et al., 2011, 2015). The false discovery rate (FDR) and the normalized enrichment score (NES) were used to sort the KEGG pathways.

### Association between the RBPS and glioma stemness

The tumor stemness index refers to the gradual loss of cell differentiation phenotype and acquisition of progenitor cell and stem-cell-like characteristics during tumor progression (Malta et al., 2018). Two types of glioma stemness indices, namely, the RNA expression-based stemness score (RNAss) and the DNA methylation-based stemness score (DNAss) were downloaded from UCSC Xena (Goldman et al., 2020) to evaluate the correlation between the RBPS and glioma stemness indices. The stemness indices range from 0 to 1, where 0 indicates a high degree of differentiation, and 1 indicates undifferentiated.

### Immune-related tumor microenvironment and potential compounds

First, the “ESTIMATE” algorithm (Yoshihara et al., 2013) was used to calculate the immune-related tumor microenvironment features from gene expression data, including stromal, immune, and ESTIMATE scores. The profiles of six immune subtype categories representing TME features and potential therapeutic and prognostic implications were downloaded from UCSC Xena (Thorsson et al., 2018). In addition, the abundance of 22 infiltrating immune cell types was calculated and inferred from RNA expression profiles using CIBERSORTx (Newman et al., 2019). Moreover, a list of important immune checkpoint molecules, including *PD-1, PDL1*, and *CTLA-4*, was obtained. In the TCGA-glioma cohort, the correlations between these immune-related features and RBPS were analyzed. Finally, to identify the potential drugs targeting these RBPS genes, drug concentration and gene expression profiles were downloaded from CellMiner (Reinhold et al., 2012) to perform correlation analysis. Drugs were filtered according to FDA's approval results for clinical trials.

### Radiomics-based TPOT analysis

Radiomics features data were downloaded from TCIA to establish an Automatic Machine Learning (AutoML) prediction model. Radiomics features were extracted from T1WI, T2WI, Flair, and T1Gd images (Bakas et al., 2017; Beers et al., 2018), including 483 usable features. Univariate logistic regression analysis evaluated the association between each Radiomics feature and RBPS in patients with glioma, and RBPS-related radiomics features were selected for autoML model training. The steps of autoML model construction include features selection, parameters selection, and final model selection, which were fully automated using the Tree-based Pipeline Optimization Tool (TPOT) (Le et al., 2020). TPOT is an automated machine learning tool based on Python, which uses genetic algorithm programming (https://github.com/rhiever/tpot) to optimize the machine learning pipeline. Before TPOT analysis, the dataset was randomly divided into a training set (99 patients) and a test set (33 patients) according to 3:1, and the random number state was fixed at 42. The training process was set as follows: generations = 100, population size = 100, and 10-fold cross-validation on the training set. Finally, TPOT will return a model with the best classification performance and parameters. The TPOT was repeated 20 times, and the models were sorted by the area under the curve (AUC). After that, the top 10 models with the best performance were screened out. In addition, ROC curves and precision-recall curves were also used to compare the performance of these ten models. By comparing the sensitivity, specificity, accuracy, AUC, and average precision (AP) of these ten models, the best model was finally determined based on the accuracy metrics (Su et al., 2019).

### Single-cell analysis

RNA-seq data of 7,925 single cells from the mouse cerebral cortex under normoxia and hypoxia conditions were analyzed using the “Seurat” package (Stuart et al., 2019). Based on pre-set filter conditions (at least 200 expressed genes but no more than 6,000 expressed genes, RNA counts >1,000, mitochondrial gene expression <20%, and hemoglobin-related gene expression <1%), a total of 7,789 cells and 14,271 gene features were filtered for further single-cell analysis. The scRNA-seq data were integrated with the “SCTransform” function and then processed using “RunPCA” and “RunUMAP” functions, including noise removal, information extraction, and cell dimension reduction. The results of cell dimensionality reduction were visualized with uniform manifold propagation and projection (UMAP) (Becht et al., 2019) to observe the effect of batch effect removal between groups. The “FindNeighbors” and “FindClusters” functions were used to detect cell clusters. Finally, each cell cluster was annotated according to the commonly used marker genes of cell types. After cell annotation, microglia, astrocytes, and pericytes were extracted as cell subsets, and “FindMarker” was used to calculate differentially expressed genes in those cell types between hypoxia and normoxia conditions.

### Single-cell regulatory network inference and clustering

Single-cell regulatory network inference and clustering (SCENIC) was used to identify the main gene regulatory networks in each cell type between different groups from single-cell transcripts (Aibar et al., 2017). First, pySCENIC (version 0.11) was used to identify the major transcription factors and their corresponding gene regulatory networks in mouse cerebral cortex cells. Transcription factors and their gene regulatory networks constitute a regulatory module called regulon. Based on the expression of transcription factors and downstream-regulated molecules in the regulon, the regulon activity score (RAS) is used to measure the regulatory ability of each regulon in each cell. Finally, based on the RAS, the regulon activity score (RSS) is calculated to describe the regulatory power of each regulon in each cell subtype, and the regulons in each cell type are ranked according to RSS so as to infer the influence of each regulon on cell transcription regulation in a specific cell type.

### Pseudotime analysis and cell trajectory inference

Monoclec3 (version 1.0) and Monoclec2 (version 2.4) (Trapnell et al., 2014; Qiu et al., 2017a,b; Cao et al., 2019) were used to calculate the pseudotime and analyze cell trajectory based on scRNA-seq transcripts from the mouse cerebral cortex for further identifying transcriptional differences among these cells and examining changes in RBPS and its transcription factors during cell fate transition. First, differentially expressed genes were determined for each cell type between normoxia and hypoxia groups. Then, the “DDRTree” method was used to calculate the cell state for each cell type. The velocyto.py (version 11.2) was used to calculate the RNA velocity in each cell. The workflow, annotation files, and visual tools can be obtained following the methods described in the previous studies (Vidal et al., 2019; Lin et al., 2021).

### Statistical analysis

All statistical analyses were performed using the R software (version 4.0.2, R Foundation for Statistical Computing, Vienna, Austria; http://www.r-project.org/) and Python (version 3.8). The “rms” R package was used to draw the nomogram. Spearman correlation coefficient and the Benjamini-Hochberg method adjusted-*p* value (FDR) were used for correlation analysis. All *p*-values were two-sided, and *p* < 0.05 was considered statistically significant.

## Results

### Differentially expressed RBP genes

First, a panel of 1,542 RBP genes was collected. Among them, 1,472 were selected to analyze the differentially expressed RBP genes between tumor and normal samples in TCGA. A total of 170 DEGs were identified according to the preset filter conditions, and the results are shown in the heat map (Supplementary Figure 1A). Subsequently, the GO and KEGG pathways for DEGs were analyzed, and the results showed that the differentially expressed RBP genes were mainly enriched in RNA processing-related pathways (Table 1). Furthermore, the protein-protein interactions (PPI) of DEGs were predicted and analyzed using the STRING website, following which a PPI sub-network analysis of DEGs was performed using the Cytoscape software (Supplementary Figures 1B,C). The core genes and molecular interaction networks related to the differential RBP genes were obtained through PPI analysis.

**Table 1:** GO function and KEGG pathway enrichment result.

**ID**	**Term**	***P*-value**
hsa03010	Ribosome	<0.001
hsa03015	mRNA surveillance pathway	<0.001
hsa03018	RNA degradation	<0.001
hsa03013	RNA transport	0.011
hsa03040	Spliceosome	0.022
GO:0000956	Nuclear-transcribed mRNA catabolic process	<0.001
GO:0006401	RNA catabolic process	<0.001
GO:0006402	mRNA catabolic process	<0.001
GO:0022626	Cytosolic ribosome	<0.001
GO:0000184	Nuclear-transcribed mRNA catabolic process, nonsense-mediated decay	<0.001
hsa03013	RNA transport	<0.001
hsa03018	RNA degradation	0.020
hsa03015	mRNA surveillance pathway	0.020
hsa04914	Progesterone-mediated oocyte maturation	0.020
hsa03008	Ribosome biogenesis in eukaryotes	0.022
hsa04114	Oocyte meiosis	0.027
hsa04962	Vasopressin-regulated water reabsorption	0.028
hsa05134	Legionellosis	0.040
GO:0008380	RNA splicing	<0.001
GO:0043484	Regulation of RNA splicing	<0.001
GO:0050684	Regulation of mRNA processing	<0.001
GO:0048024	Regulation of mRNA splicing, *via* spliceosome	<0.001
GO:0000377	RNA splicing, *via* transesterification reactions with bulged adenosine as nucleophile	<0.001

### Identification of the 6-RBP gene signature

**Figure 1:**
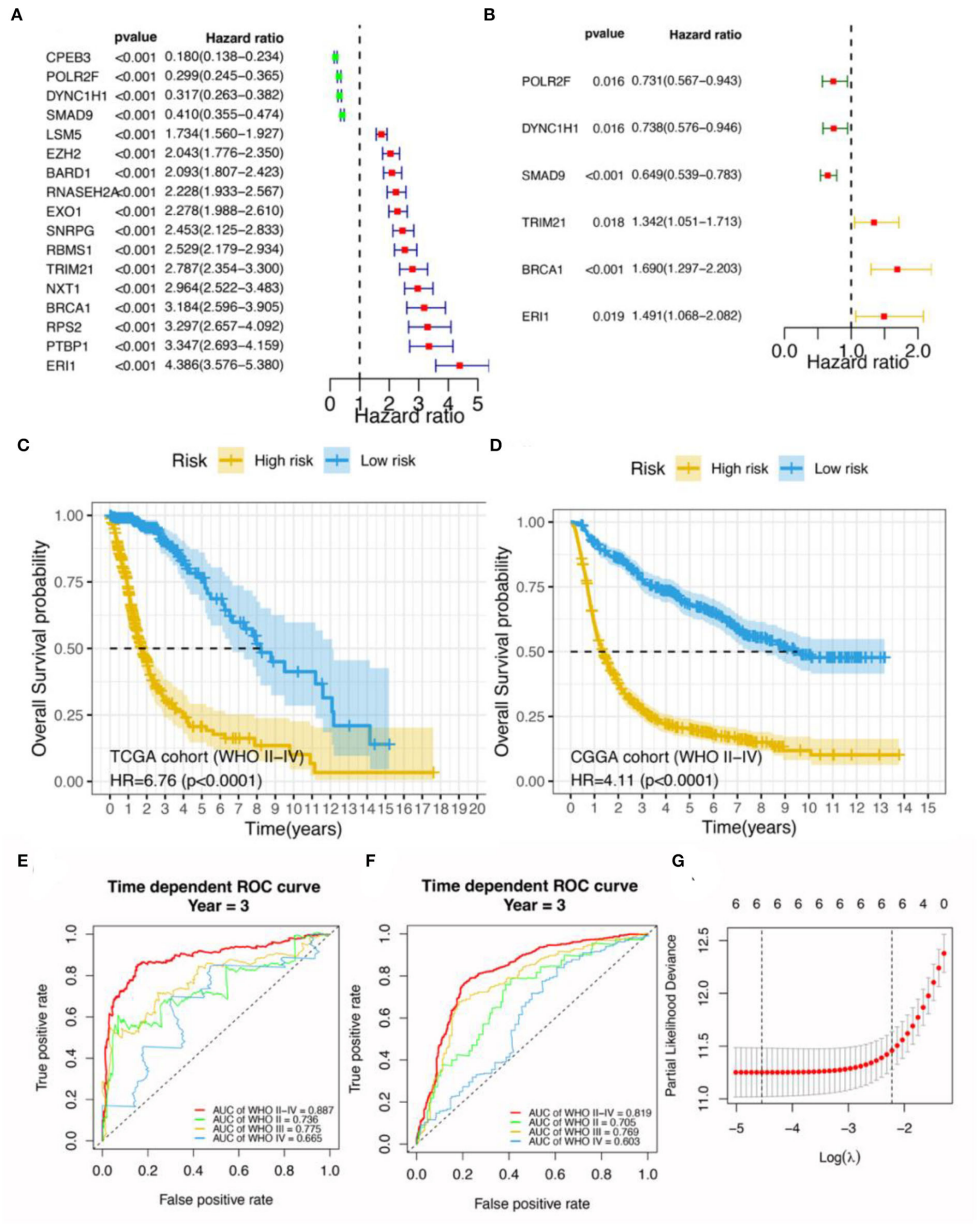
Construction of a prognostic signature based on OS events in glioma. **(A)** Univariate Cox analysis for the top 17 RBP genes. **(B)** Multivariate Cox analysis for six RBP genes. Survival analysis for patients with glioma between the high- and low-risk groups in **(C)** TCGA and **(D)** CGGA datasets. Yellow indicates high risk and blue indicates low risk for glioma. Bioinformatics analyses for the 6-gene risk stratification signature; receiver operator characteristic curve analysis for the 6-gene signature in **(E)** TCGA and **(F)** CGGA datasets. **(G)** Selection of the tuning parameter (lambda) in Cox-penalized regression analysis *via* 10-fold cross-validation in the TCGA cohort. The vertical dotted lines on the left and the right indicate “lambda.min” and “lambda.1se” criteria, respectively. The red dots represent partial likelihood deviation values, while the gray lines are the corresponding standard errors. AUC, the area under the curve.

**Figure 2:**
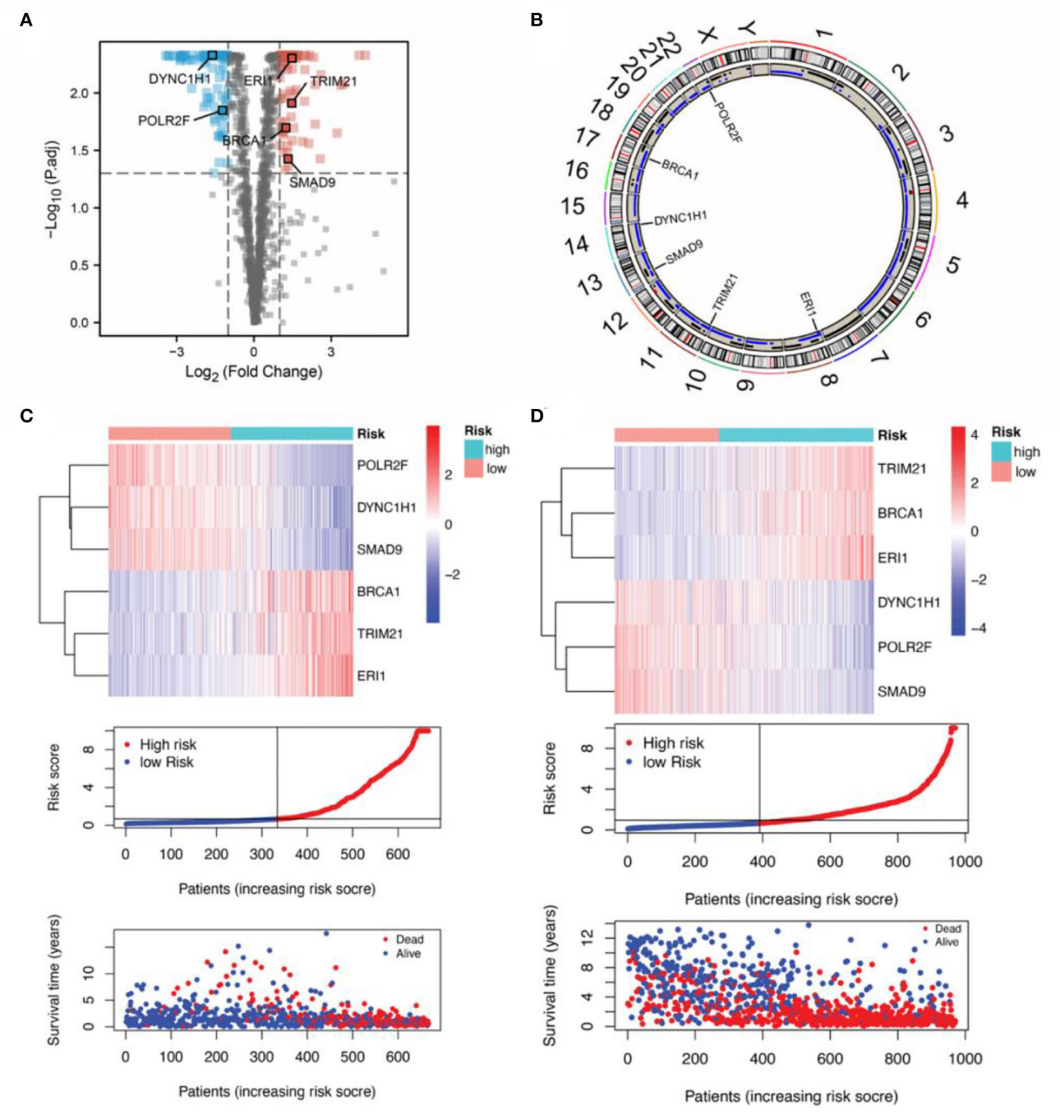
Characteristics of the six RBP genes in the RBPS. **(A)** Differently expressed genes between the normal and tumor samples are shown in the volcano plot. The dots in red represent upregulated genes (Yang et al., 2020), while those in green are signed downregulated genes (Boucas et al., 2015) in tumor samples. Significant differences were determined using the thresholds of |log_2_ FC|> 1 and FDR < 0.05. **(B)** The location of the six RBP genes on the 24 chromosomes, as well as the copy number variation events. The expression of the six RBP genes, distribution of the RBPS risk scores, survivor status, and survival time of the patients with glioma ranked by their risk scores in **(C)** TCGA and **(D)** the CGGA datasets.

First, 170 differentially expressed RBP genes were screened and the commonly shared intersecting genes in RNA expression profiles of patients with glioma in TCGA, CGGA, and GSE72951 datasets were obtained. The filtered expression profiles from these three datasets were further processed to remove batch effects. Next, by univariate Cox analysis for TCGA glioma expression profile data, a total of 100 RBP genes were analyzed along with the total survival time data, and the top 17 RBP genes significantly related to survival were screened out (Figure 1A). Finally, in the TCGA training set, the Lasso penalty Cox regression analysis was performed to screen gene variables, and a prognosis model was constructed according to the multivariate Cox regression model. Using the lambda.min threshold (Figure 1G), a 6-RBP gene signature (RBPS) was identified, comprising 6 RBP genes (*TRIM21, BRCA1, ERI1, POLR2F, DYNC1H1*, and *SMAD9*). The RBPS was associated with the adverse OS in glioma. The volcanic plot showed the differential analysis results of these six RBP genes, which showed that *POLR2F* and *DYNC1H1* were downregulated in glioma, while *TRIM21, BRCA1, ERI1*, and *SMAD9* were upregulated in glioma (Figure 2A). Figure 2B showed the copy number variation of these six RBP genes and their positions on 24 chromosomes. The RBPS risk score (RS) was calculated based on the linear combination of the expression values of the six RBP genes multiplied by their corresponding coefficients. The formula for calculating the RBPS RS was as follows:


(2) $$\begin{array}{l} RS_{RBPS}=0.294\times EXP_{TRIM21}+0.525\times EXP_{BRCA1}\\ +0.400\times EXP_{ERI1}-0.313\times EXP_{POLR2F}\\ -0.303\times EXP_{DYNC1H1}-0.432\times EXP_{SMAD9} \end{array}$$

Among the six RBP genes constituting the RBPS, higher expression levels of *POLR2F, DYNC1H1*, and *SMAD9* were associated with a lower risk of death (HR < 1). In contrast, higher expressions of *TRIM21, BRCA1*, and *ERI1* were associated with poorer overall survival (HR > 1; Figure 1B; Supplementary Table 1). Patients with glioma were stratified according to the median value of the RBPS risk score in the TCGA cohort and were divided into high-risk and low-risk groups. The 5-year OS rates for RBPS-derived high- and low-risk patients were 19 and 75%, respectively; WHO II-IV (HR: 6.76, 95% CI: 4.84–9.44; *p* < 0.001), WHO II (HR: 3.47, 95% CI: 1.68–7.18, *p* < 0.001), WHO III (HR: 2.85, 95% CI: 1.75–4.64, *p* < 0.001), WHO IV (HR: 1.54, 95% CI: 1.04–2.26, *p* = 0.028; Figure 1C; Supplementary Figures 2A–C).

Subsequent validation in the CGGA dataset showed the outcomes were consistent with the findings in the TCGA cohort; WHO II-IV (HR: 4.11, 95% CI: 3.40–4.95; *p* < 0.001), WHO II (HR: 2.02, 95% CI: 1.32–3.10; *p* = 0.001), WHO III (HR: 3.12, 95% CI: 2.32–4.19; *p* < 0.001), and WHO IV (HR: 1.28, 95% CI: 1.03–1.60, *p* = 0.027; Figure 1D; Supplementary Figures 2D–F). These findings indicated that Subsequent validation in tRBPS could predict adverse prognosis for patients with glioma as well as the glioma subgroups based on the WHO grades. In addition, the gene expression differences for these six RBP genes with respect to copy number variation events were analyzed (Supplementary Figures 3A–F). The results showed that copy number variants were significantly associated with mRNA expressions of *POLR2F* (*p* < 0.001), *DYNC1H1* (*p* < 0.001), *TRIM21* (*p* < 0.001), *SMAD9* (*p* < 0.001), and *ERI1* (*p* = 0.005). This suggests that copy number variants may be an important factor in the poor prognosis of RBPS.

### RBPS is associated with a poor OS for glioma

In the TCGA discovery cohort, the RBPS showed robustness for identifying the poor survival of gliomas, as evidenced by the good AUC values for WHO grades: WHO II-IV (AUC = 0.887, 95% CI: 0.854–0.937), WHO II (0.736, 95% CI: 0.577–0.919), WHO III (0.775, 95% CI: 0.7–0.893) and WHO IV (0.665, 95% CI: 0.417–0.855; Figure 1E). Similarly, in the CGGA validation cohort, the AUC value of RBPS for identifying poor OS prognoses in all patients with glioma was 0.819 (95% CI: 0.794–0.851): WHO II (0.705, 95% CI: 0.622–0.799), WHO III (0.769, 95% CI: 0.721–0.829), and WHO IV (0.603, 95% CI: 0.507–0.687; Figure 1F). These results indicated that the RBPS had a potential clinical value, and the gene signature comprised of the six RBP genes could be used to identify the adverse OS in patients with glioma with various WHO grades. Additionally, in the CGGA validation cohort, the expression of the six RBP genes, survival status, and survival time distribution for patients according to their RBPS risk scores are shown in Figures 2C,D.

### RBPS is an independent predictor of glioma risk and survival outcome

To further evaluate the performance of RBPS as a clinical marker for risk stratification, its utility was analyzed along with clinical features for predicting survival and prognosis. First, in the TCGA cohort, univariate and multivariate Cox regression analyses were performed for various clinical features, including age, sex, WHO grade, and histopathology, along with the RBPS. In univariate analysis, age (*p* < 0.001), WHO grade (*p* < 0.001), histopathology (*p* < 0.001), and RBPS (*p* < 0.001) were important predictors for adverse OS (Supplementary Figure 3G). Subsequently, multivariate Cox regression analysis showed that age (*p* < 0.001), grade (*p* < 0.001), and RBPS (*p* < 0.001) were independent risk factors in predicting adverse OS in patients with glioma (Supplementary Figure 3H). In the CGGA cohort, univariate and multivariate cox regression analyses were conducted. Apart from age, WHO grade, and histopathology, the clinical features included radiotherapy, chemotherapy, IDH mutation, 1p19q codeletion, and methylation status of the MGMT gene promoter region (MGMTp). The results showed that WHO classification (*p* < 0.001), age (*p* = 0.012), and RBPS (*p* < 0.001) remained independent risk factors in predicting adverse OS (Supplementary Figures 3I,J). These results verified that RBPS based on these six RBP genes was reliable in predicting OS and could be used as an independent predictor of survival outcomes in patients with glioma.

The GSE72951 dataset included patients with recurrent glioblastoma only. In this dataset, K-M analysis showed that the median survival time in the high-RBPS-risk group was longer than that in the low-RBPS-risk group (*p* = 0.010, Supplementary Figures 4A,B), while univariate and multivariate Cox analyses suggested no statistical correlation between RBPS and survival outcomes (Supplementary Figures 4C,D). According to statistical significance and comparison of RBPS risk scores of WHO IV glioma in the three data sets, it was speculated that the RBPS risk scores of patients with WHO IV glioma in the GSE72951 dataset were relatively close to each other, thereby resulting in no statistically significant correlation between RBPS and survival outcomes (Supplementary Figures 4E–L). In addition, the expressions of protective genes (*POLR2F, DYNC1H1*, and *SMAD9*) for glioma in the GSE72951 dataset increased, while those of the risk genes (*TRIM21, BRCA1*, and *ERI1*) decreased so that the risk scores of patients in GSE72951 were the lowest among the three groups, but the median overall survival time was the shortest among the three datasets. The survival time of patients with WHO IV in the GSE72951 data set was the shortest, which could be attributed to the fact that the total survival time in this data set was calculated from the first recurrence and could be related to the inclusion of patients with recurrent glioblastoma. In addition, these patients received CCNU and/or bevacizumab treatment, which may be the reason why gliomas in the GSE72951 data set have lower RBPS risk scores. These findings suggested that the RBPS risk score may show dynamic changes with chemotherapy, which may, in turn, reflect the therapeutic efficacy.

### Construction of a nomogram for predicting the OS for patients with glioma

**Figure 3:**
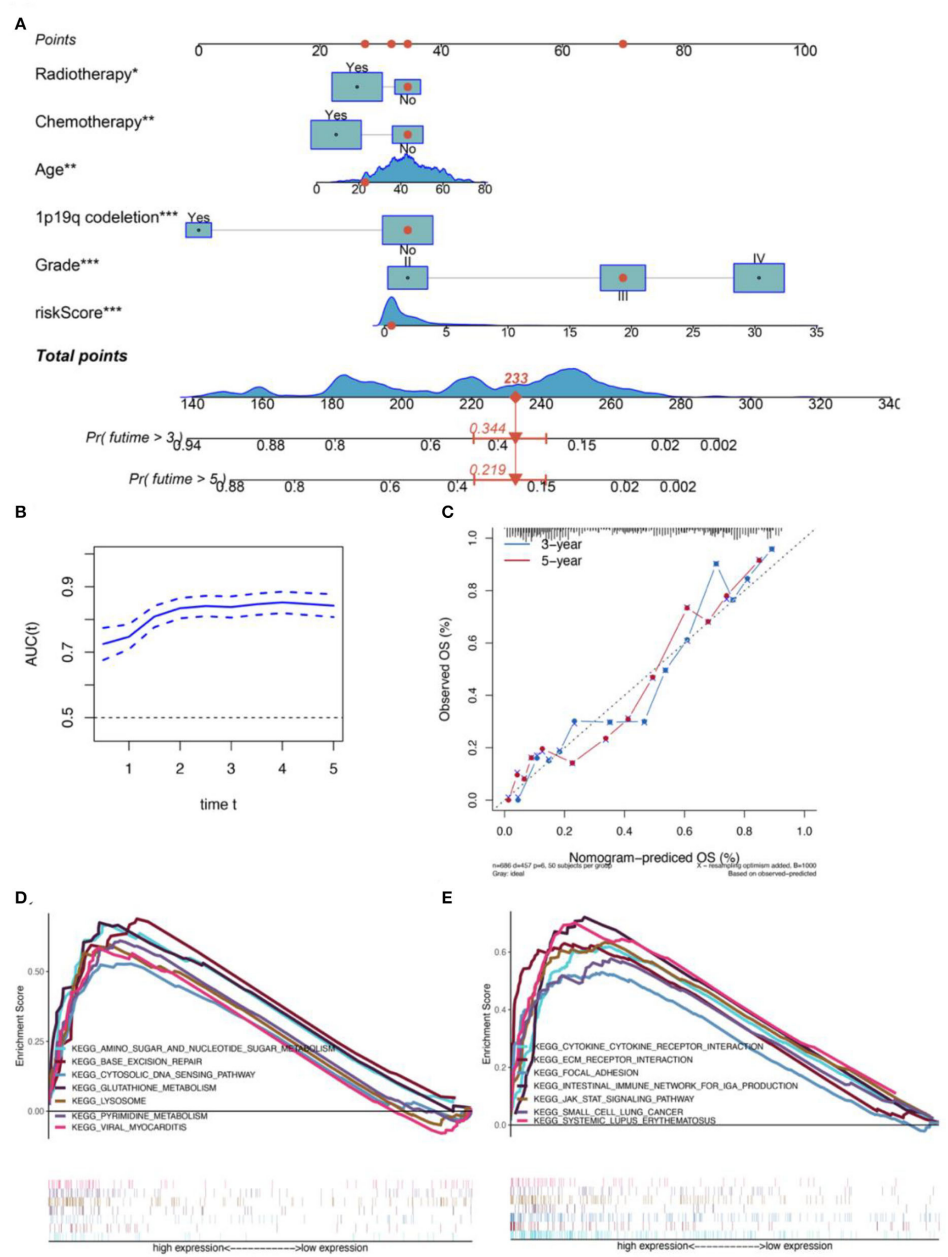
Construction of a CGGA-based clinical prediction model. **(A)** The nomogram for predicting the 3- and 5-year overall survival of patients with glioma based on the six independent prognostic factors from the CGGA dataset. **(B)** Relationship between the AUC values for the prognostic prediction model and the correspondingly predicted survival times. **(C)** The calibration plot shows that the prediction using the model is in good agreement with the actual situation. **(D)** Glutathione metabolism, an amino sugar, and nucleotide sugar metabolism, lysosome, pyrimidine metabolism, viral myocarditis, base exception repair, and cytosolic DNA-sensing path were significantly differentially enriched between the high- and low-risk-score groups in the TCGA dataset. **(E)** JAK-STAT signaling, ECM-receptor interaction, cytokine-cytokine receptor interaction, systematic lupus erythematosus, intestinal immune network for IgA production, focal adhesion, and small cell lung cancer pathways were differentially enriched between the high- and low-risk groups in the CGGA database.

In order to further improve the predictive ability and applicability of RBPS in clinical practice, RBPS, and other critical clinical features (WHO grade, age, radiotherapy, chemotherapy, and 1p19q codeletion) were used to construct a multivariate Cox regression model and a risk nomogram for ease of use in clinical settings for predicting survival probabilities of patients with glioma. The parameters of this model are listed in Table 2. As shown in Figure 3A, the total score was calculated based on the sum of scores for each factor. The higher the total score, the lower the OS rate for 1 year, 3 years, and 5 years. As shown in the example (the red dot) in the figure, a patient with WHO grade III and an RBPS risk score of 1 (wherein no radiotherapy, no chemotherapy, and no 1p19q codeletion all corresponded to 34 points, WHO grade III corresponded to 70 points, and the RBPS risk score of 1 corresponded to 33 points in the nomogram), the total score corresponding to all characteristics was 233 points, and the predicted survival probabilities for 3 years and 5 years based on this total score were 0.344 and 0.219, respectively. Figure 3B shows the AUC of the model between 0.74 and 0.85 for predicting the overall survival rate for 1–5 years. The calibration curve showed that the predicted values using the model were in good agreement with the actual values (Figure 3C), suggesting a good prediction performance.

**Table 2:** Prediction factors for survival in glioma.

**Variables**	**Prediction model**		
	**β**	**Hazard ratio (95% CI)**	***P* value**
Grade (III vs. II)	1.074	2.928 (2.506–3.421)	<0.001
Grade (IV vs. II)	1.753	5.773 (4.905–6.795)	<0.001
Age	0.011	1.011 (1.007–1.014)	0.006
Radiotherapy (yes vs. no)	−0.252	0.777 (0.689–0.876)	0.035
Chemotherapy (yes vs. no)	−0.357	0.7 (0.623–0.785)	0.002
1p19q codeletion (yes vs. no)	−1.043	0.352 (0.3–0.413)	<0.001
Risk score	0.062	1.064 (1.048–1.079)	<0.001

β is the Cox regression coefficient. For grade, radiotherapy, and 1p19q codeletion, HR represents the average HR over the entire time period.

### Gene set enrichment analysis for RBPS

GSEA was performed using MSigDB Collection [c2. cp.kegg. v7.1. symbols (curated)] to identify differentially expressed signaling pathways in gliomas between high- and low-risk groups of patients with glioma. All genes were ranked according to their fold changes between the high- and low-risk groups, following which a GSEA was performed. FDR < 0.05 was used to filter and select significant enrichment signaling pathways. The results showed that a high RBPS risk score was related to the carcinogenesis of glioma, including multiple pathways related to cellular metabolism, immunity, and proliferation (Figures 3D,E). Furthermore, based on the sharing signaling pathways in TCGA and CGGA datasets, GSEA showed that the RBPS was associated with cytokine-cytokine receptor interaction (TCGA: NES = 1.72, size = 264, FDR = 0.046; CGGA: NES = 1.91; size = 209; FDR = 0.037) and intestinal immune network for IgA production (TCGA: NES = 1.75, size = 46, FDR = 0.048; CGGA: NES = 1.86; size = 42; FDR = 0.040). Taken together, the activity of immune, metabolic, and proliferative pathways may be enhanced, which may be related to the enhanced carcinogenic phenotype in patients with a high RBPS risk score.

### Relationship between RBPS and glioma stemness

**Figure 4:**
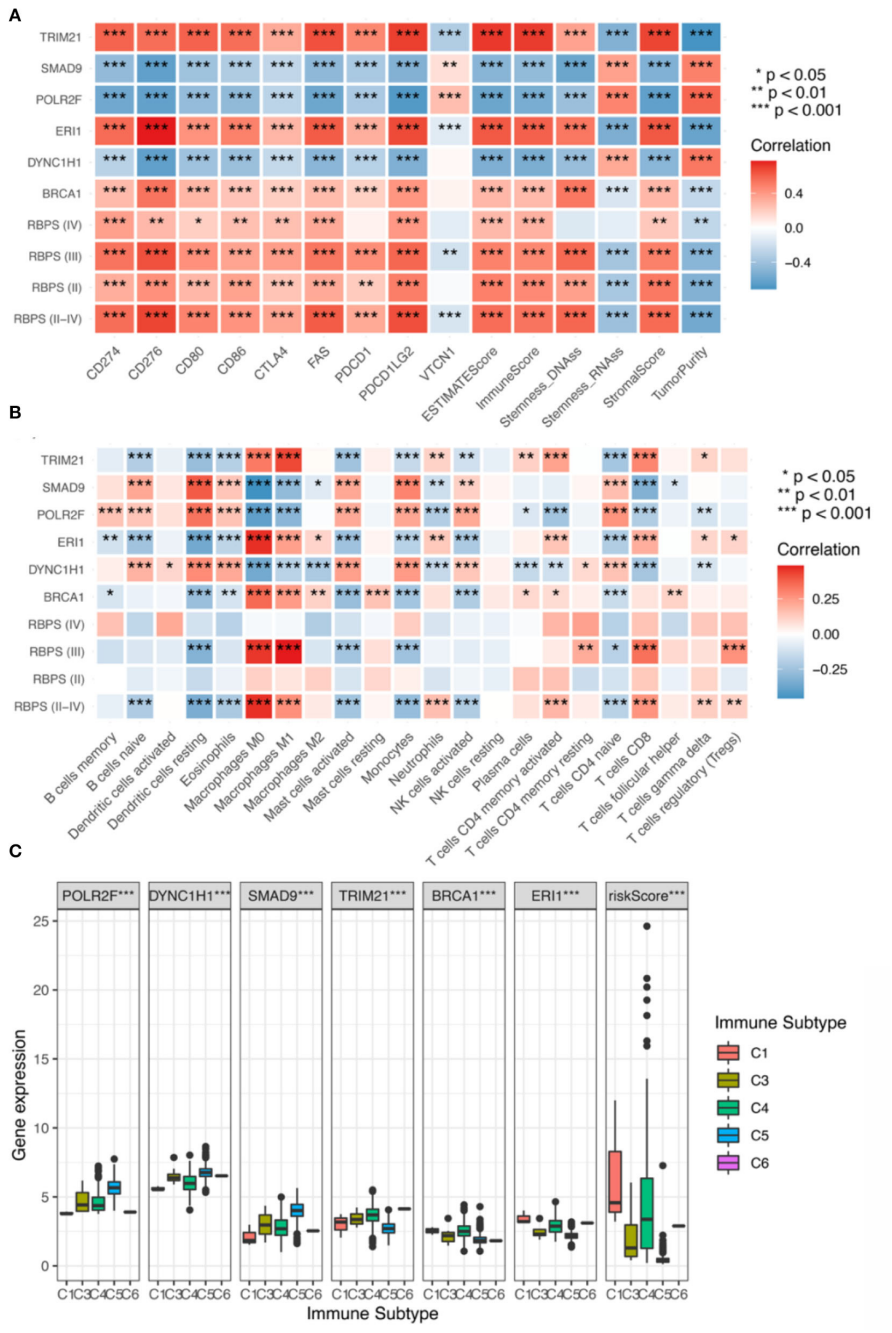
**(A)** Correlation analysis for the expression of the six RBPs and the RBPS with stemness (RNAss and DNAss), TME (the stromal score, the immune score, the ESTIMATE score, and tumor purity), and immune checkpoints (*CD274, CD276, CD80, CD86, CTLA4, PDCD1, PDCD1LG2*, and *VTCN1*). Correlation analysis of RBPS for Grades II, III, and IV glioma, respectively; red: positive correlation and blue: negative correlation. Relationship of the expressions of the six RBP genes (*POLR2F, DYNC1H1, SMAD9, TRIM21, BRCA1*, and *ERI1*) and RBPS with **(B)** infiltration of eight types of immune cells (B cells, CD8^+^ T cells, CD4^+^ T cells, NK cells, monocytes, macrophages, dendritic cells, neutrophils), and **(C)** immune subtypes in TCGA.

To evaluate the relationship between RBPS and tumor stemness of glioma, the correlation of the RBPS score with DNAss and RNAss was calculated (Figure 4A). In all WHO grade II-IV gliomas, DNAss was positively correlated with the RBPS score, *ERI1, BRCA1*, and *TRIM21*, while negatively correlated with *POLR2F, DYNC1H1*, and *SMAD9* [Spearman correlation, Benjamini-Hochberg (BH)-adjusted *p* < 0.05]. However, RNAss was negatively correlated with the RBPS score, *ERI1, BRCA1*, and *TRIM21*, while positively correlated with *POLR2F, DYNC1H1*, and *SMAD9* (Spearman, BH-adjusted *p* < 0.05). In WHO grade II and III gliomas, the correlation of RBPS with DNAss and RNAss also showed a similar pattern in the overall glioma cohort. However, no significant correlation between RBPS and stemness index was observed in WHO grade IV gliomas, which may be attributed to their high malignancy and stemness.

### Correlation between RBPS and tumor microenvironment

GSEA showed that RBPS was associated with immune-related pathways. In order to evaluate the relationship between RBPS and the immune microenvironment of glioma, the correlation between RBPS and immune-related characteristics was analyzed. Figure 4A shows that RBPS and these six RBP genes were significantly correlated with the stromal score (Spearman, BH-adjusted *p* < 0.05), the immune score (Spearman, BH-adjusted *p* < 0.05), the ESTIMATE score (Spearman, BH-adjusted *p* < 0.05), and tumor purity (Spearman, BH-adjusted *p* < 0.05), as, also, tumors of all WHO subtypes.

As shown in Figure 4B, significant correlations between RBPS and individual immune cell types were observed. Specifically, RBPS was positively correlated with CD8^+^ T cells, M1 and M0 macrophages, activated memory CD4^+^ T cells, regulatory T cells, γδ T cells, and neutrophils (Spearman, BH-adjusted *p* < 0.05), and negatively correlated with naive B cells, naive CD4^+^ T cells, eosinophils, activated mast cells, activated NK cells, monocytes, and dendritic cells (Spearman, BH-adjusted *p* < 0.05). In addition, the RBPS scores and the expressions of the six RBP genes were significantly different among the immune subtypes C1, C3, C4, C5, and C6 (the Kruskal-Wallis test, *p* < 0.05; Figure 4C). Among the WHO subtypes of glioma, the expression differences for RBPS and the six RBP genes among different immune subtypes were analyzed, and the results are illustrated in Supplementary Figure 5.

To further elucidate the potential role of RBPS in immunotherapy, the correlations of RBPS and six RBP genes with common immune checkpoint molecules were analyzed. The results showed that, for glioma, the RBPS scores were positively correlated with the expression of immune checkpoint molecules, *PDCD1, CD274, PDCD1LG2, CTLA4, CD86, CD80, CD276*, and *FAS* (Spearman, BH-adjusted *p* < 0.05) but negatively correlated with *VTCN1* (Spearman, BH-adjusted *p* < 0.05; Figure 4C). In WHO grades II, III, and IV gliomas, RBPS was positively correlated with *CD274, CD276, CD80, CD86, CTLA4, FAS*, and *PDCD1LG2*. Finally, to identify potential drugs that targeted RBPS, the potential drugs related to the expression of these six RBP genes were queried in the database, and a correlation analysis was performed. The top 16 compounds with the highest correlation with the six RBP genes are shown (Supplementary Figure 5D). As shown, the top 16 predicted compounds were mainly related to *DYNC1H1* and *POLR2F*.

### Radiomics features for RBPS and automatic machine learning prediction model

**Figure 5:**
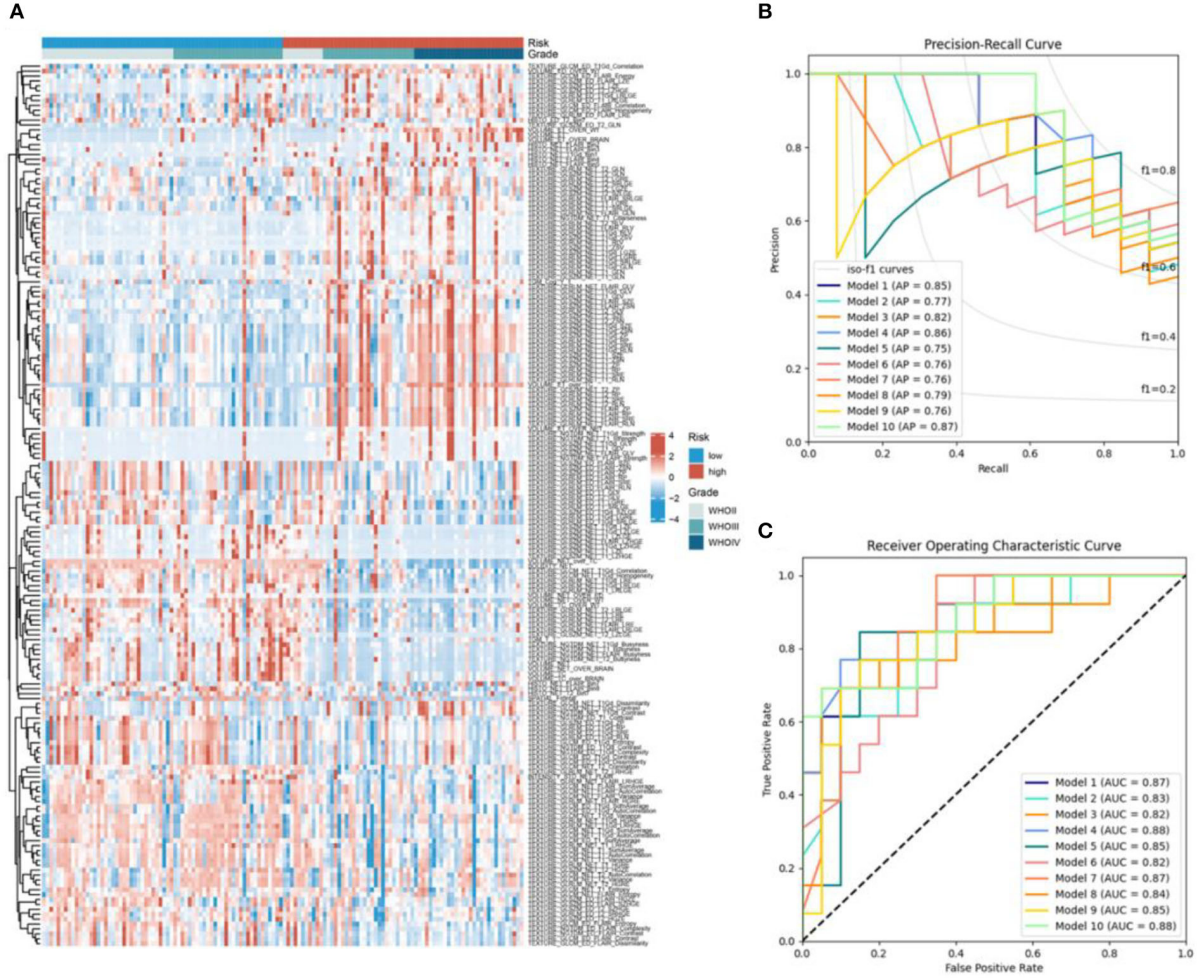
**(A)** The heatmap of the 180 radiomics features between the high- and low-RBPS-risk-score samples. **(B)** Receiver operating characteristic curves and **(C)** precision-recall curves for 10 models based on the testing set. AP, average precision; AUC, area under the curve.

First, by univariate logistic regression analysis, 180 radiomics features were selected according to *p* < 0.05 and included in the automatic machine learning model (Figure 5A). When splitting the training and test sets from the whole dataset to reduce the randomness in selecting patients for high- and low-RBPS risk between training different models and comparing their performances, the samples of the two sets were fixed (the random state was set at 42) and standard TPOT was performed. TPOT was used to calculate the average cross-validation score (AC) for each model in the training set (each model was trained 100 times/generation) and return the model with the best accuracy in the test set. Finally, by repeating the TPOT process ten times, ten independent classificatory models were obtained to predict the risk stratification according to RBPS. Overall, these 10 models showed good classification performances in training and test sets, along with high accuracy (Accuracy, ACC) (Supplementary Tables 2, 3). During the training process, each model showed the following performance in training and test sets: Model 1 (AC = 0.829, ACC = 0.727), Model 2 (AC = 0.868, ACC = 0.758), Model 3 (AC = 0.868, ACC = 0.667), Model 4 (AC = 0.829, ACC = 0.697), Model 5 (AC = 0.858, ACC = 0.818), Model 6 (AC = 0.859, ACC = 0.697), Model 7 (AC = 0.858, ACC = 0.697), Model 8 (AC = 0.839, ACC = 0.727), Model 9 (AC = 0.848, ACC = 0.788), Model 10 (AC = 0.829, ACC = 0.727), and 10 Average of models generated based on TPOT (AC = 0.848, ACC = 0.736). Among them, according to the accuracy in the test set, Model 5 was selected as it showed the best classification performance. Figures 5B,C show the average accuracy (AP) and the area under the curve (AUC) for the 10 models in the test set. The parameters of Model 5 are as follows: Model [5] = make_pipeline [binarizer (threshold = 0.3), OneHotEncoder (minimum_fraction = 0.15, sparse = false, threshold = 10], GradientBoostingClassifier [the learning_rate = 0.5, max_depth = 8, max_features = 0.3, min_samples_leaf = 1, min_samples_split = 3, n_estimators = 1t00, subsample = 0.95)]. In this model, Binarizer and OneHotEncoder were used to process the radiomics features (see Supplementary Table 4 for detailed parameter descriptions of the other nine models).

### The six RBP genes are associated with ischemic stroke, dementia, and aging

**Figure 6:**
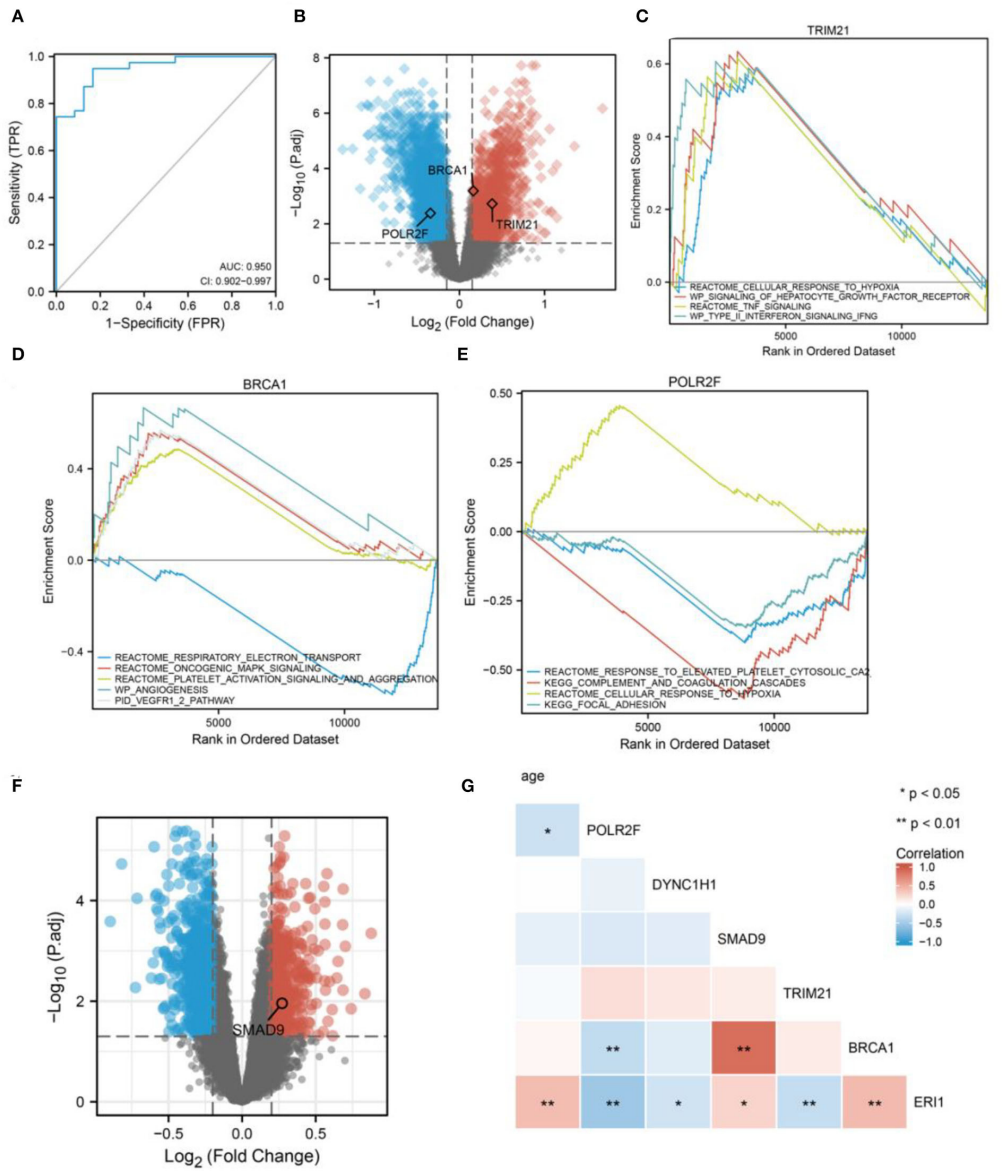
Diagnostic efficacy of the six RBP genes for IS. **(A)** Diagnostic efficacy of the six RBP genes for IS using blood samples (AUC = 0.950, 95% CI: 0.902–0.994). **(B)** The volcano plot shows the DERBPs associated with IS. GSEA for **(C)** *TRIM21*, **(D)** *BRCA1*, and **(E)** *POLR2F* in IS. **(F)** The volcano plot shows *SMAD9* is associated with dementia. **(G)** The correlation heatmap shows that aging correlates with *POLR2F* and *ERI1* expression.

In order to examine the potential role of RBPS genes in ischemic stroke, RNA transcripts from peripheral blood samples of 39 patients with ischemic stroke and 24 healthy controls were analyzed. The six RBP genes included in the RBPS could distinguish IS from the healthy control group (Figure 6A, AUC = 0.950). Differentially expressed analysis showed that *POLR2F, BRCA1*, and *TRIM21* in this RBPS were associated with ischemic stroke. Among them, *TRIM21* and *BRCA1* were upregulated, while *POLR2F* was downregulated in IS (Figure 6B). GSEA showed that the upregulation of *TRIM21* was significantly related to upregulated pathways, including (REACTOME) response to elevated platelet cytosolic Ca^2+^, (REACTOME) cellular response to hypoxia, (KEGG) complex and coagulation cascades, and (KEGG) focal adhesion (Figure 6C). In *BRCA1*-upregulated samples, (REACTOME) oncogenic MAPK signaling, (REACTOME) platelet activation signaling and aggregation, (WP) angiogenesis, and the (PID) VEGFR1 and VEGFR2 pathway were upregulated, while the (REACTOME) respiratory electron transport pathway was downregulated significantly (Figure 6D). In *POLR2F*-upregulated samples, (REACTOME) response to elevated platelet cytosolic Ca^2+^, (KEGG) complement and coagulation cascades, and (KEGG) focal adhesion pathways were downregulated, while (REACTOME) cellular response to hypoxia was upregulated (Figure 6E). In IS, the upregulation of *TRIM21* was related to platelet function activation, increased coagulation, and response to hypoxia. Upregulation of *BRCA1* was related to tumor progression, platelet activation, and angiogenesis. The downregulation of *POLR2F* was accompanied by an upregulation of platelet reaction and coagulation, and downregulation of hypoxia-related response.

Further analyses revealed that *SMAD9* in the RBPS was associated with the Alzheimer's disease onset (Figure 6F). In addition, aging was positively associated with *ERI1* expression and negatively with *POLR2F* expression (Figure 6G).

### Cell clustering shows the highest proportion of microglia and astrocytes

**Figure 7:**
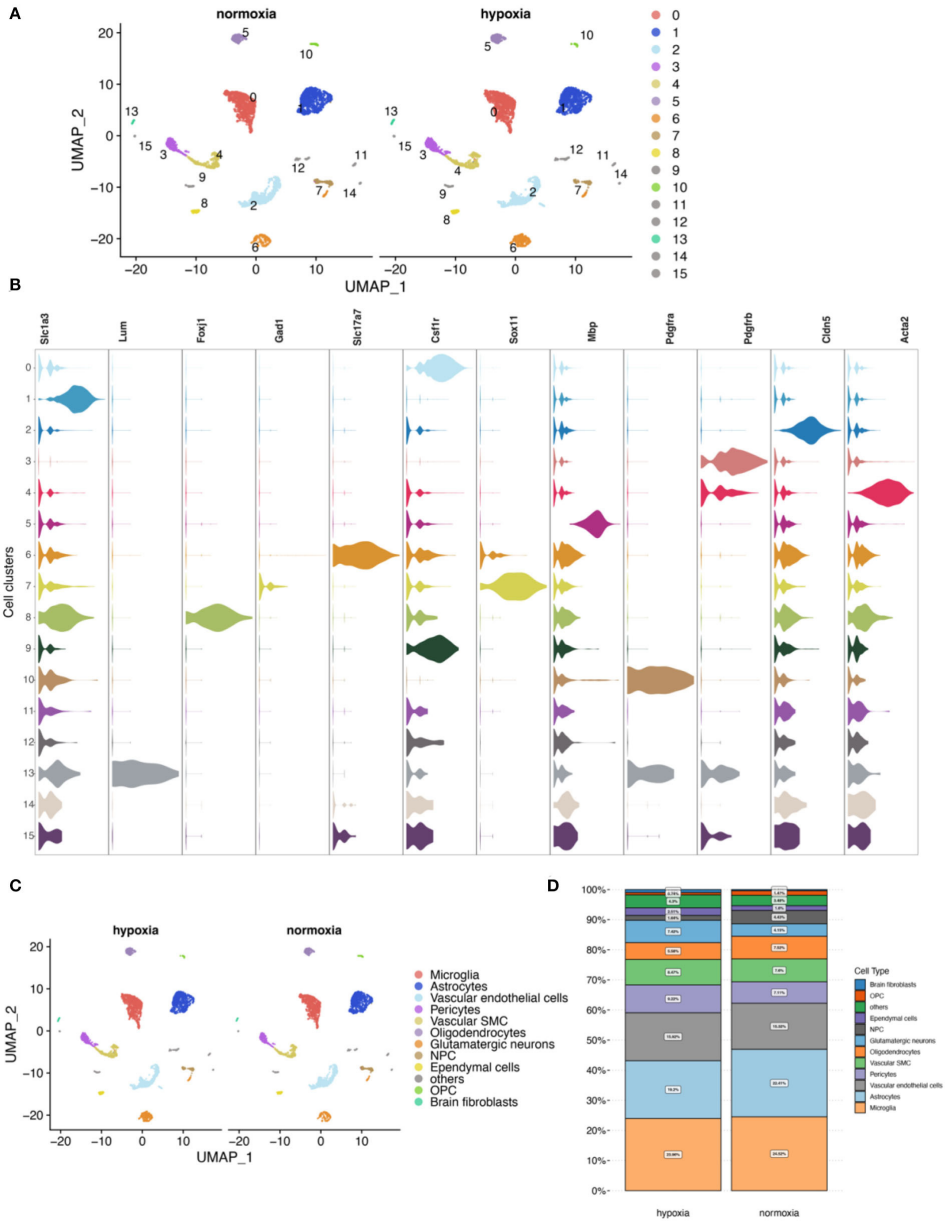
Cell clustering and annotation for the mouse cerebral cortex. **(A)** Single-cell analysis shows 16 cell clusters in hypoxic and normoxic conditions. **(B)** Expression of marker genes for cell annotation between the cell clusters. **(C)** Cell annotation results show 11 cell types. **(D)** Comparison of proportions of cells between the normoxia and the hypoxia groups.

First, quality control for single-cell RNA-seq (scRNA-seq) data (Supplementary Figures 6A–G) was performed according to cell characteristic distributions and preset quality filtering conditions. UMAP showed no significant batch effects for cells between the two groups after integration analysis (Supplementary Figure 6H). By clustering, 16 cell clusters were finally identified, and 11 cell types were annotated (Figures 7A–C; Supplementary Figures 6I, 7). Among them, microglia and astrocytes had the highest proportion (Figure 7D).

### RBPS-related genes associated with pseudotime in microglia

**Figure 8:**
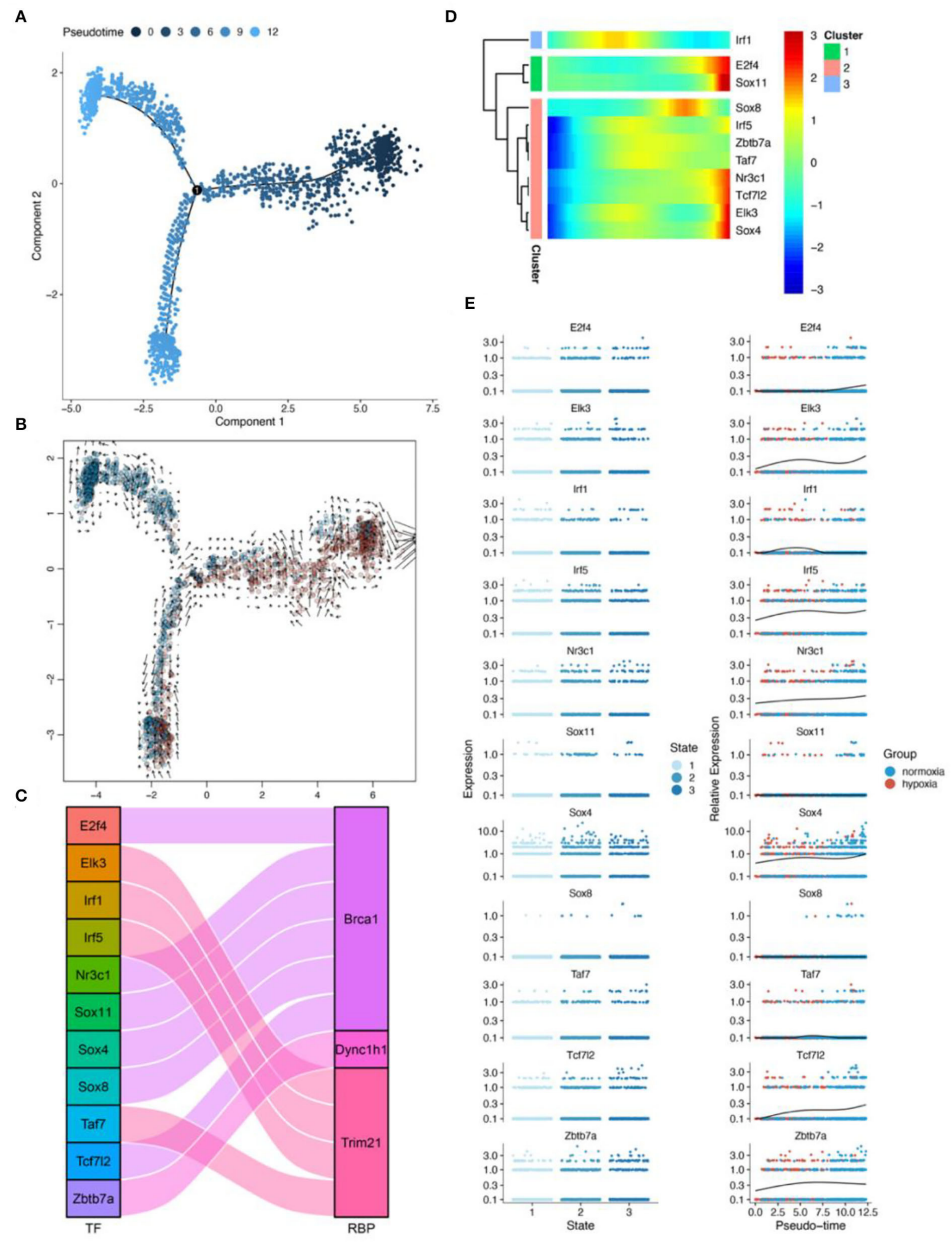
Results of pseudotime analysis for microglia. **(A)** The pseudotime distribution plot of microglia. **(B)** The RNA velocity plot; the longer is the arrow, the stronger is the transcriptional activity. **(C)** The Sankey diagram shows the RBPS-related transcription factors (*E2f4, Elk3, Irf1, Irf5, Nr3c1, Sox11, Sox4, Sox8, Taf7, Tcf712*, and *Zbtb7a*), which are associated with the pseudotime. **(D,E)** Changes in the expression of these RBPS-related transcription factors with changes in pseudotime.

Pseudotime analysis showed three main cell stages of microglia at normal- and low-oxygen concentrations (Figures 8A,B, Supplementary Figure 8). In microglia, *Sox4* and *Tcf7l2*, which regulated *Brca1*, and *Irf5*, which regulated *Trim21*, were significantly related to the pseudotime of these cells (Figures 8C,D). The expression of transcription factors and RBPs in microglia during the transition from hypoxia to normal oxygen concentrations (Figures 8A,E) were observed using the pseudotime distribution plot. *Sox4, Irf5*, and *Tcf7l2* were downregulated at the early stages of pseudotime but upregulated at normal-oxygen concentrations. These results suggested that *Sox4* and *Tcf7l2*, which regulated *Brca1*, and *Irf5*, which regulated *Trim21*, may change under hypoxia, thus participating in cellular phenotypic changes.

### RBPS-related genes associated with pseudotime in astrocytes

**Figure 9:**
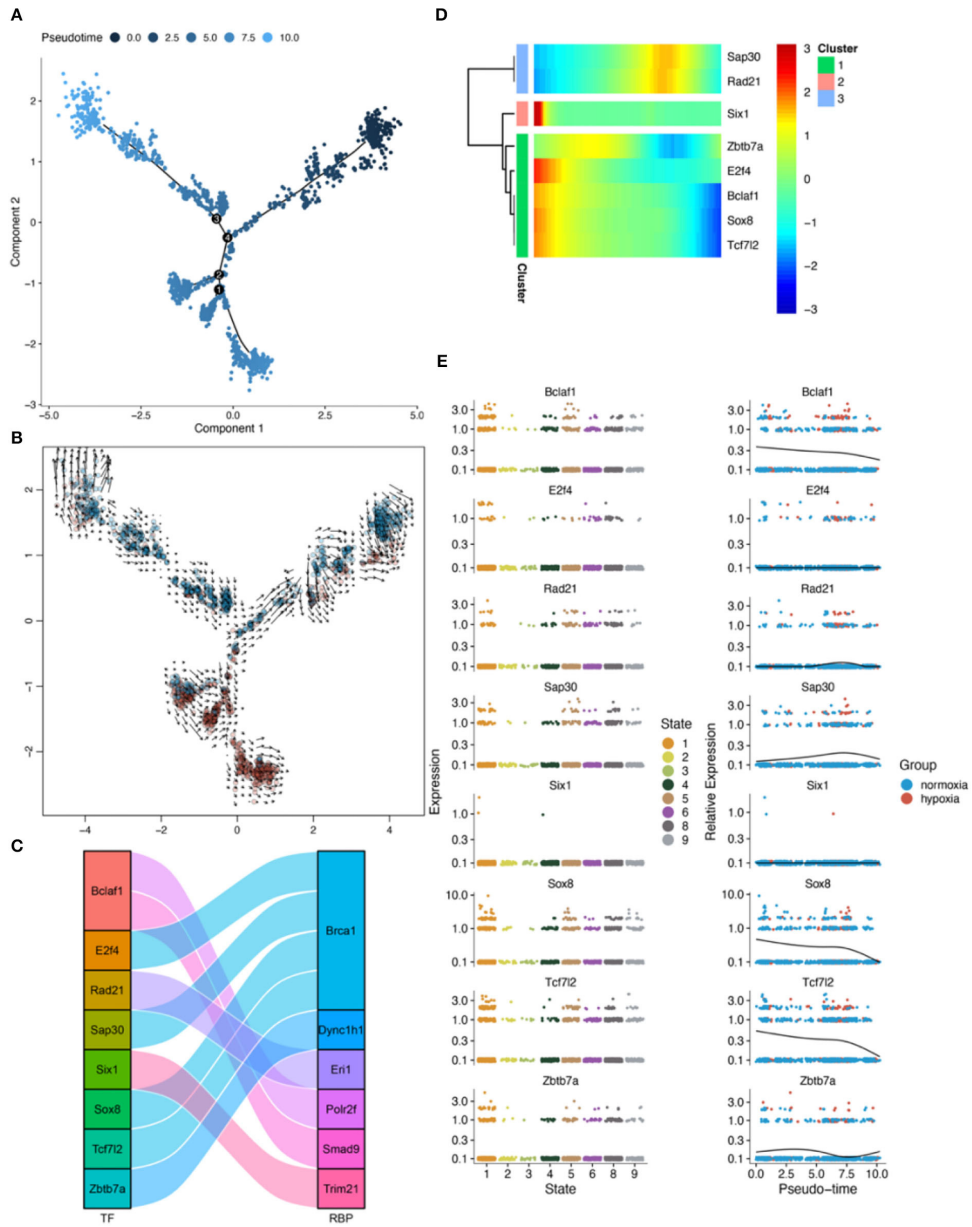
Results of pseudotime analysis for astrocytes. **(A)** The pseudotime distribution plot of astrocytes. **(B)** The RNA velocity plot, wherein the longer the arrow, the stronger the transcriptional activity. **(C)** The Sankey diagram shows the RBPS-related transcription factors (*Bclaf1, E2f4, Rad21, Sap30, Six1, Sox8, Tcf712*, and *Zbtb7a*), which are associated with the pseudotime. **(D,E)** Changes in the expression of these RBPS-related transcription factors with changes in pseudotime.

Pseudotime analysis showed that astrocytes went through eight major cell stages in normal-oxygen concentration and hypoxia conditions (Figures 9A,B; Supplementary Figure 9). According to SCENIC analysis, a regulatory relationship between *Tcf7l2* and *Brca1* was observed (Figure 9C). In astrocytes, *Tcf7l2* was an important pseudotime-related gene (Figure 9D). In addition, from the pseudotime distribution map, astrocytes were found in the early, middle, and late pseudotime stages in the normal oxygen concentration group, while, in the hypoxia group, astrocytes were dominant in the middle stage and lesser in the early and late stages; *Tcf7l2* increased in the early stages and decreased toward the later stage (Figures 9A,E). These results suggested that (*Brca1*-related) *Tcf712* may play a role in the transition to a hypoxic environment.

### RBPS-related genes associated with pseudotime in pericytes

**Figure 10:**
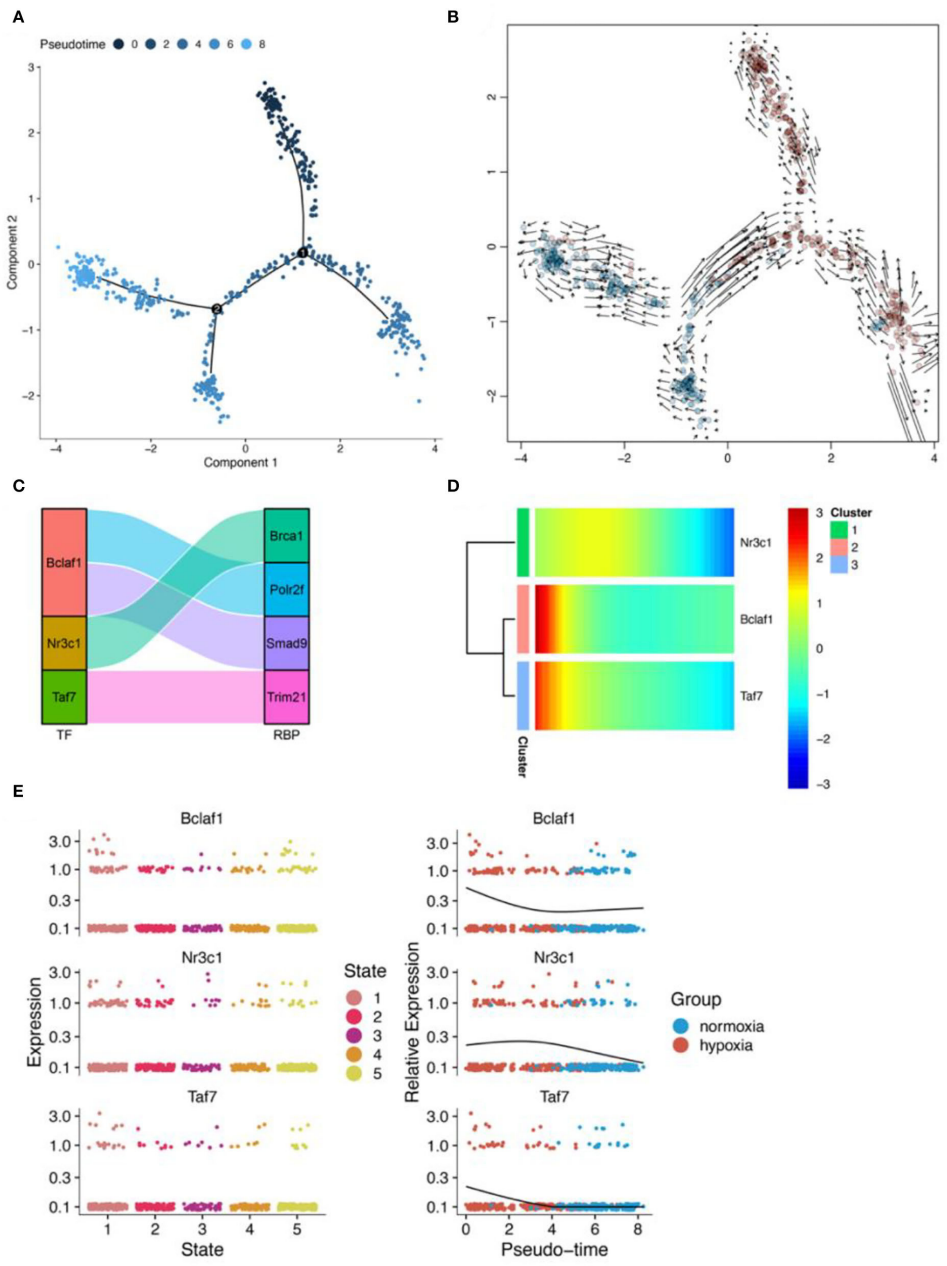
Results of pseudotime analysis for pericytes. **(A)** The pseudotime distribution plot of pericytes. **(B)** The RNA velocity plot, wherein the longer the arrow, the stronger is the transcriptional activity. **(C)** The Sankey diagram shows the RBPS-related transcription factors (Bclaf1, Nr3c1, Taf7), which are associated with the pseudotime. **(D,E)** Changes in the expression of these RBPS-related transcription factors with changes in pseudotime.

Pseudotime analysis showed that pericytes went through six cell stages (Figures 10A,B; Supplementary Figure 10) in normal oxygen concentration and hypoxia conditions. According to the prediction of the gene regulatory network by SCENIC analysis, a regulatory relationship between *Taf7* and *Trim21* was obtained (Figure 10C). In astrocytes, *Taf7* was an important pseudotime-related gene (Figure 10D). In addition, as shown in the pseudotime distribution plot, pericytes were obviously stagnating in the early pseudotime stages under hypoxia (Figure 10A). *Taf7* increased at an early stage of pseudotime but decreased toward the end stage; pericytes under hypoxia were mostly dominant in the early stage of pseudotime (Figure 10E). These results suggested that *Taf7* may play an important role in cell-state transition between hypoxia and normal oxygen conditions.

### Pseudotime-related regulons

**Figure 11:**
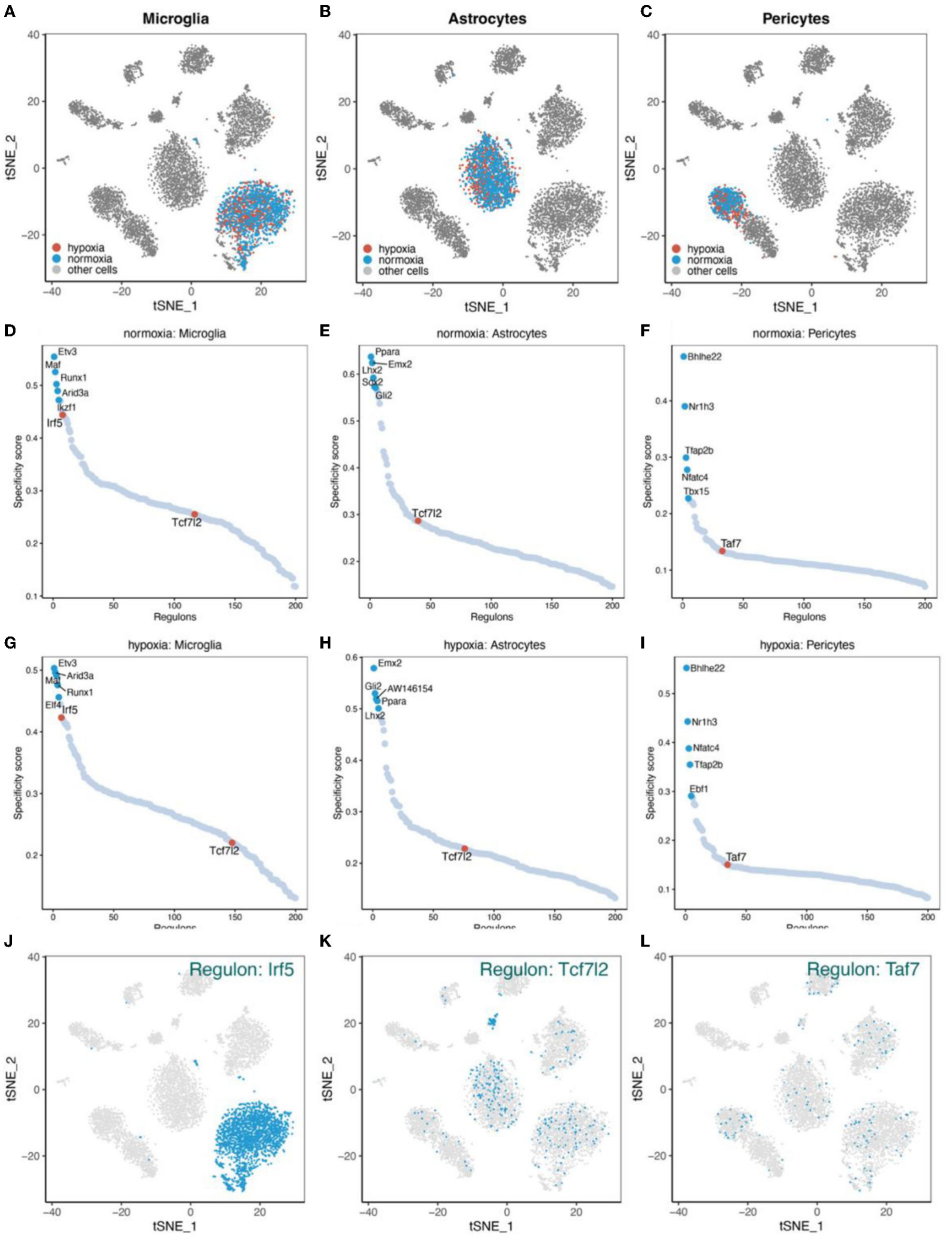
Major regulons in microglia, astrocytes, and pericytes. **(A–C)** tSNE shows the distribution of microglia, astrocytes, and pericytes. **(D–F)** Major gene regulatory networks in the three types of cells under normoxia condition, wherein red dots represent the gene regulatory networks regulated by corresponding transcription factor related to the RBPS. **(G–I)** Major gene regulatory networks in the three types of cells under hypoxia condition. **(J–L)** Distribution of regulons associated with the RBPS in cells.

SCENIC analysis was performed for single-cell data to identify important regulons of each cell subtype. In the SCENIC analysis flow, UMAP and tSNE showed single-cell dimension reduction results and the distributions for each cell type (Supplementary Figure 11). Figures 11A–C show the distribution of microglia, astrocytes, and pericytes under normal- and low-oxygen conditions. Irf5 was an essential and specific regulon of microglia in the normal oxygen and hypoxia concentration groups (Figures 11D,G,J). The RSS and rank of Tcf7l2 were related to oxygen concentration. The rank of Tcf7l2 in the normoxia group was higher than that in the hypoxic group (Figures 11E,H,K). The Taf7 regulon played a regulatory role in many other cells apart from pericytes (Figures 11F,I,L).

## Discussion

The RBP family of proteins plays an important regulatory role in glioma and IS (Shao et al., 2013; Zhou et al., 2014; Barbagallo et al., 2018; Lan et al., 2020; Si et al., 2020; Zhang et al., 2020; Sharma et al., 2021); however, there is a lack of a systematic analysis of the role of RBP in both these diseases. Herein, we describe a set of previously unreported six RBP genes that can be used to predict the prognosis of glioma and diagnostic classification for IS. In particular, we found that the RBPS was associated with tumor immunosuppression in glioma and hypoxia and coagulation in IS. In addition, automatic machine learning was used to predict the risk stratification based on RBPS in glioma. In this RBPS, *SMAD9* was found to be associated with dementia; *POLR2F* and *ERI1* were identified to be associated with aging. In view of hypoxia as the basis of common models for studying glioma and IS, the expressions of these six RBP genes in microglia, astrocytes, and pericytes, along with their gene regulatory networks, were analyzed using single-cell data from the mouse cerebral cortex. The six RBP genes and the transcription factors in their gene regulatory networks were analyzed using pseudotime analyses between normal oxygen and hypoxia conditions. *Irf5*/*Trim21* and *Tcf712*/*Brca1* in microglia, *Tcf712*/*Brca1* in astrocytes, and *Taf7*/*Trim21* in pericytes were identified as RBPS-related genes that were regulated in response to hypoxia. These new findings indicated that RBPs, post-transcriptional regulators, are essential regulatory molecules involved in the underlying common pathways in the development of glioma and IS.

### Significance of identification of molecular markers for glioma

Glioma is the most common primary intracranial tumor with high mortality, among which glioblastoma is the most malignant type (Liu et al., 2020). According to molecular genetic characteristics, some important glioma subtypes, including IDH mutation, TERT promoter, and 1p/19q codeletion, improve the therapeutic efficacy for glioma (Wang et al., 2020). It is worth trying to identify biomarkers that are robust and can guide the treatment and predict a prognosis so as to stratify the patients according to the risk and help choose appropriate treatment methods.

### Role of RBP in tumors

Previous studies have shown that RBP plays a vital role in tumor progression. For example, in the progression of HCC, global changes in RBP are more evident than those of transcription factors (Dang et al., 2017). In immunity, RBP CAPRIN1 promotes innate immunity mediated by IFN-γ-STAT1 by stabilizing the Stat1 mRNA (Xu H. et al., 2019). In addition, some studies suggest that the genetic system of RBP dysfunction can provide methods for describing different immunological conditions (Kafasla et al., 2014). In AML, the effects of RBM39 deletion on splicing further lead to preferential lethality for AML with spliceosome mutations, which provides a strategy for the treatment of those carrying RBP-splicing mutations (Wang E. et al., 2019; Villanueva et al., 2020). In glioma, although some studies report several RBPs related to a poor prognosis of these patients (Boucas et al., 2015; Bhargava et al., 2017; Barbagallo et al., 2018; Velasco et al., 2019; Wang J. et al., 2019; Lan et al., 2020; Wang et al., 2020), their potential clinical application, including for an individual prognostic risk assessment, lacks systematic evaluation. In the research on RBPs, some new technologies have been developed to enrich and extract RBPs and their homologous RNAs, such as the orthogonal organic phase separation (OOPS) (Villanueva et al., 2020), which is a fast, efficient, and reproducible method to purify cross-linked RNA-protein complexes in an unbiased manner, thus making it more efficient for identifying and studying new RBPs. Taken together, we first developed a risk stratification gene signature based on RBP gene expression profiles and an automatic machine learning prediction model based on radiomics for individualized risk assessment of patients with glioma. Below, we discuss the roles of these core RBPs in glioma genesis.

### Identification of RBPS and the role of the six RBP genes in glioma

First, six prognostic-related RBP genes (*POLR2F, DYNC1H1, SMAD9, TRIM21, BRCA1*, and *ERI1*) were obtained from the TCGA-glioma dataset. Based on these six RBP genes, a 6-RBP gene signature (RBPS) with risk stratification characteristics was constructed. Among them, *POLR2F, DYNC1H1*, and *SMAD9* in tumor tissues of patients with glioma were downregulated as compared to normal tissues adjacent to cancer, while *TRIM21, BRCA1*, and *ERI1* were upregulated. In literature, only *BRCA1, TRIM21*, and *POLR2F* have been implicated in the progression of glioma (Rasmussen et al., 2016; Yang et al., 2020; Zhao et al., 2020). Breast cancer susceptibility gene (*BRCA*) mutations, including *BRCA1*, are found in several tumors (Sun et al., 2020). Umphlett et al. (2020) reported a case of a patient with GBM with extensive metastases, whereby *BRCA1* (p.I571T) was considered the possible driving mutation. Through bioinformatics analyses based on the GSE53733 dataset, Yang et al. (2020) report that *POLR2F* is one of the four potential key genes that affect the OS in GBM. Higher levels of *TRIM21* expression are associated with a poor prognosis of glioma and promote proliferation, drug resistance, and migration of glioma cells (Zhao et al., 2020). *SMAD9* mutations have been reported in the progression of gastrointestinal ganglioma. In addition, a low expression of SMAD9 is related to a poor OS in lung adenocarcinoma (Ngeow et al., 2015; Zhai et al., 2021). The microtubule motor protein encoded by *DYNC1H1* is involved in many cellular processes, such as mitosis and intracellular transport. *DYNC1H1* mutations have been implicated in nervous system diseases (Hoang et al., 2017) and pancreatic cancer (Furukawa et al., 2011), and these mutations are consistent with a high immune activity of tumor mutation load in various cancer types (Bai et al., 2020). In addition, *DYC1H1* is upregulated in gastric cancer (Gong et al., 2019) and downregulated in primary gallbladder carcinoma (Huang et al., 2014). In mice, Eri1 is a histone mRNA-related protein involved in RNA metabolism pathways and various cellular processes regulated by RNA (Thomas et al., 2014). Declercq et al. (2020) show that the exogenous nuclease, ERI1, interacts with PB2, PB1, and NP components of the viral ribonucleoprotein, thus promoting viral transcription. Previous studies have reported that gene copy number variations in glioma may lead to changes in RBP gene expression (Bhargava et al., 2017), which was also observed in this study. In addition, in tumor stemness, RBPS was positively correlated with DNAss but negatively correlated with RNAss. For results of RBP genes and tumor stemness, we speculated that, due to the characteristics of post-transcriptional regulation of RBPs, the correlations of DNAss and RNAss with RBP would be different, and the underlying mechanism needs to be elucidated in the future.

### A high risk of RBPS in glioma is related to immunosuppression

GSEA showed differences in immune-related functional pathways between high- and low-RBPS-risk-score groups. By evaluating the relationship between RBPS and immune-related characteristics, it was possible to improve the understanding of the anti-tumor immune intervention and highlight feasible immunotherapeutic strategies. Therefore, the associations of RBPS with the tumor microenvironment, immune subtypes, immune cell types, and immune checkpoint molecules were further analyzed. Xu et al. (2021) report that higher stromal and immune scores predict a poor prognosis in patients with LGG. In LGG and GBM of this study, gliomas with higher RBPS risk are related to higher immune and stromal scores, thus indicating that the RBPS index was related to immune responses in gliomas. A previous study reports that macrophage infiltration indicates a worse OS in GBM (Iglesia et al., 2016). Differentiated GBM cells promote GSC-dependent tumor progression by enhancing macrophage infiltration into tumor tissues (Uneda et al., 2021). RBPS also showed a positive correlation with the proportion of infiltrated macrophages in the tumor, which indicated that RBPS may play a potential role in the involvement of macrophage infiltration in the development of glioma. Conventional type-1 dendritic cells (cDC1) play an important role in immunotherapy-mediated reactivation of tumor-specific CD8^+^ T cells to promote tumor regression (Liang et al., 2021). In this study, RBPS was negatively correlated with dendritic cell infiltration and positively correlated with CD8^+^ T cells, which implied that, in gliomas with a high RBPS risk, a complete CD8^+^ T cell reactivation for immunotherapy may be difficult due to the lack of dendritic cells, thus making the anti-tumor effects difficult to be achieved. Due to several reasons, including inherent challenges in drug application, a unique immune environment of the brain, and heterogeneity between and within tumors, immune checkpoint blockade therapy has not been effective for GBM (Khasraw et al., 2020). A comprehensive understanding of the unique tumor microenvironment of the brain is important for glioma immunotherapy with immune checkpoint blockade (Qi et al., 2020). In this study, RBPS was positively correlated with the expressions of *CD274, CD276, CD80, CD86, CTLA4, FAS, PD1*, and *PDL1* in gliomas, indicating that the expressions of immune checkpoint-related genes increased with a high RBPS risk, thus leading to a worse prognosis. The relationship between RBPS and immune checkpoint molecules needs further studies.

### Automatic machine learning model predicts RBPS

Generally, the RBPS showed reliable prognostic value for predicting the OS and immune-related characteristics of glioma and comprised only six RBP genes, making its clinical translation convenient. Recently, with the development of computing power, researchers have tried to replace some expensive molecular detection techniques using MR image-based artificial intelligence so as to stratify the risk of tumor phenotypes, screen patients with cancer, and predict their responsiveness to treatment (Acs et al., 2020). Therefore, using MRI-based radiomics features, we developed an automatic machine learning classification model to predict the risk of RBPS in glioma, thus making the molecular signature more convenient and attractive for preoperative evaluation.

### Diagnostic performance and the roles of the six RBP genes in IS

The prediction model based on the six RBP genes from blood samples could also predict the occurrence of IS, suggesting their association with IS. Among the six RBP genes, *TRIM21* and *BRCA1* were upregulated in IS, while *POLR2F* was downregulated in IS. Functional pathway enrichment analysis showed that *TRIM21* upregulation was related to platelet activation, enhanced coagulation, and response to hypoxia. Previous studies have shown that *TRIM21* is mainly expressed in hematopoietic cells, wherein it is induced by IFNs in case of infections and autoimmune diseases (Sjöstrand et al., 2013). Pan et al. (2016) show that TRIM21 modulates redox homeostasis through the ubiquitination of p62, and *TRIM21*-deficient cells exhibit enhanced antioxidant responses and reduced cell death under oxidative stress. In addition, TRIM21 deficiency induces naive T cells to differentiate into Th17 and promotes IL-17 expression, along with a stable atherosclerotic plaques phenotype formation (Brauner et al., 2018). In cerebral ischemia/reperfusion (I/R), BRCA1 overexpression can alleviate or prevent nerve injury caused by I/R due to reduced production of reactive oxygen species (ROS) and lipid peroxidation (Xu et al., 2018). Overexpression of BRCA1 in neural stem cells (NSCs) reduces apoptosis and oxidative stress after the oxygen-glucose deprivation/reoxygenation (OGD/R) insert, stimulating their proliferation, thus improving the therapeutic effects of NSC transplantation in cases of ischemic stroke (Xu P. et al., 2019). Genome-wide association analysis shows that *POLR2F* (22q13.1) is associated with periventricular white matter hyperintensions (PVWMH), and PVWMH are associated with ischemic stroke (Armstrong et al., 2020).

### Dual action of ROS under hypoxia

In the cerebrovascular unit, hypoxia can induce astrocytes, microglia, pericytes, and neuronal cells to produce ROS and reactive nitrogen species (RNS) (Sumbayev and Yasinska, 2007; Chen et al., 2013). ROS and RNS play dual roles in the neurovascular unit, destroying tissues and macromolecules upon injury (global cerebral ischemia and reperfusion injury) while promoting cellular proliferation, tissue repair and regeneration, and angiogenesis in the recovery stage (acute ischemic stroke and hypoxic tumor core) (Kalogeris et al., 2014).

### The role of hypoxic stress in tumor immunity and angiogenesis

In tumors, hypoxic stress plays an important role in tumor progression and immune escape by controlling angiogenesis, promoting immunosuppression, and tumor resistance (Noman et al., 2015). Several hypoxia-induced immunosuppressive cells in the hypoxic zones of solid tumors, such as myeloid-derived suppressor cells, tumor-associated macrophages (MDSCs), and T-regulatory (Treg) cells, have been reported (Mantovani et al., 2002; Ohta et al., 2011). Hypoxia increases MDSC-mediated T cell tolerance by upregulating the tumoral MDSC expression of PD-L1 (Noman et al., 2014); hypoxia-inducible factor-1 (HIF-1) is the primary regulator of PD-L1 (Barsoum et al., 2014). Hypoxia decreases the expression of several molecular markers of differentiation and maturation of DCs in response to lipopolysaccharide and inhibits the stimulating ability of DCs to activate T cell functions (Mancino et al., 2008). In addition, VEGF produced by human tumors can inhibit the functional maturation of DCs and promote the escape of tumor cells (Gabrilovich et al., 1996). Hypoxic stress increases the lytic functions of CD8^+^ T cells and decreases their proliferation and differentiation (Noman et al., 2015). Hypoxia attracts Treg cells to the tumor bed by affecting the distribution of cytokines in the tumor microenvironment and enhancing the immunosuppressive functions of Treg cells (Noman et al., 2015). For cancer stem cells (CSCs), hypoxia and HIFs are considered to induce tumor cells to dedifferentiate into immature phenotypes and maintain their stemness (Kallergi et al., 2009; Semenza, 2012). In this study, RBPS in glioma was related to tumor immunosuppression. Glucose and amino acid metabolism increased in gliomas with a high RBPS risk score. This may serve the increased demand for energy and oxygen of highly proliferating tumor masses. In addition, among these six RBP genes, the upregulation of *TRIM21* and *BRCA1* in IS was related to angiogenesis and responses to hypoxia. The upregulation of *TRIM21* and *BRCA1* and the downregulation of *POLR2F* were related to platelet activation and increased coagulation, thus suggesting that an imbalance among these genes may result in a state of hypercoagulation, which could easily lead to an ischemic cerebral infarction. Along with aging, *POLR2F* was downregulated, while *ERI1* was upregulated. In IS, downregulated *POLR2F* was associated with downregulation of pathways in response to hypoxic responses, implying that *POLR2F* may be associated with aging-related hypoxic stress.

Astrocytes, microglia, and pericytes are important cells that maintain brain homeostasis. In order to observe the regulation and potential mechanism underlying the six RBP genes in the hypoxic environment at the cellular level, the gene regulatory network in various cell types was investigated.

### Gene regulatory networks in astrocytes

Astrocytes are the most abundant cell types in the central nervous system. As an integral part of the neuron-glial system, astrocytes serve as housekeeping functions, including the formation of the blood-brain barrier (BBB), regulation of cerebral blood flow, repair of blood vessels (Williamson et al., 2021), and the resistance to oxidative stress (Blanc et al., 1998; Ransom and Ransom, 2012). After an ischemic stroke, reactive astrogliosis involving astrocytes exerts harmful and beneficial effects on neuronal survival and nerve recovery (Liu and Chopp, 2016; Xu et al., 2020). The upregulation of *BRCA1* in IS was related to tumor promotion, platelet activation, and angiogenesis. On examining the gene regulatory network in astrocytes, *Brca1* was identified in Tcf712 regulon that was upregulated under hypoxia. These results suggested that *Tcf712*/*Brca1* may play an important role in the response of astrocytes to hypoxic stress.

### Gene regulatory networks and immune responses of microglia

As resident macrophages in the central nervous system, microglia are the first immune cells that perceive and respond immediately during cerebral ischemia (Lambertsen et al., 2019). During a stroke, with the dynamic changes in pathology, microglia undergo polarization (Tsuyama et al., 2018). According to the phenotypic changes in microglia, they can be roughly classified into pro-inflammatory (M1) or anti-inflammatory (M2) types (Ransohoff, 2016). The interferon regulatory factor (IRF) family of proteins has an important relationship with microglial polarization after stroke (Zhao et al., 2017; Al Mamun et al., 2018). For instance, IRF4 negatively regulates inflammation and promotes M2 polarization of macrophages (Eguchi et al., 2013), while IRF5 induces M1 polarization (Paun et al., 2008). Recent studies have shown that the IRF5-IRF4 regulatory axis in microglia regulates neuroinflammation after ischemic stroke and affects stroke outcomes (Al Mamun et al., 2020). IRF5 mediates pro-inflammatory activation of microglia and affects anti-inflammatory responses (Fan et al., 2020; Al Mamun et al., 2021). We found that Irf5 regulon was an important regulator in microglia, and *Trim21* was a downstream molecule in this gene regulatory network. The expression of *Irf5* was downregulated under hypoxia, which may be related to the time of experimental conditions, suggesting that the main microglia types may be changing toward the anti-inflammatory phenotype after living in 7.5% oxygen concentration for 7 days. The Tcf712 regulon was another important gene regulatory network identified in microglia, and Brca1 was a member of this network. The expression of *Tcf712* was also downregulated under hypoxia.

### Multiple microvascular regulatory functions of pericytes

Pericytes play an important role in regulating various microvascular functions, such as angiogenesis (Winkler et al., 2011), the formation and maintenance of the BBB (Armulik et al., 2010; Daneman et al., 2010), capillary blood flow regulation (Hall et al., 2014; Korte et al., 2022), neuroinflammatory regulation (Stark et al., 2013; Korte et al., 2022), glial plaque formation (Göritz et al., 2011), and stem cell characterization (Özen et al., 2014; Nakagomi et al., 2015). Pericytes are important therapeutic targets in stroke, glioma, Alzheimer's disease, spinal cord injury, and other diseases due to their vital role in the nervous system diseases (Cheng et al., 2018). Variable permeability of BBB can be observed in the high cell proliferation regions, which may be related to an increase in the NG2-expressing pericytes herein (Jackson et al., 2017). In addition, hypoxic regions of tumors recruit activated pericytes through the regulation of hypoxia-inducible factors (Svensson et al., 2015). In acute ischemic stroke, pericyte HIF-1 can destroy BBB and affect the prognosis of stroke (Tsao et al., 2021). In addition, glioma stem cells can also differentiate into pericytes, thus supporting BBB integrity and angiogenesis (Cheng et al., 2013; Segura-Collar et al., 2021). Under hypoxia, *in vitro*, pericytes derived from the human brain acquire a microglial phenotype and are a new source of inflammatory cells during cerebral ischemia (Özen et al., 2014). Interestingly, *Trim21* (Irf5 and Taf7 regulon) is present in different gene regulatory networks of microglia and pericytes in response to hypoxia. This suggests that RBPs, as post-transcriptional regulators, participate in different regulatory pathways, thus performing various cellular functions.

Owing to the heterogeneity of pericytes (Armulik et al., 2011), the selection of specific cell markers and the correct identification of pericytes in “-omics” studies pose a challenge (Cheng et al., 2018). Using transcriptomics and proteomics, mRNA and protein expressions in pericytes at different positions of the capillary bed would be accurately defined.

This study is the first attempt to comprehensively evaluate the role of RBPs in glioma and IS using computational biology, thus providing a panoramic map of a panel of genes between the two diseases and a research paradigm for the study of such scientific issues. Using bulk RNA-seq and scRNA-seq data, we examined the important roles of a panel of RBP genes in glioma and IS and identified the relationship between the two diseases. In this study, a prognostic RBPS consisting of six RBP genes was identified for glioma. These six RBP genes obtained from blood samples had a high classificatory performance for diagnosing IS. RBPS was associated with immunosuppression, enhanced energy metabolism, and tumor growth in glioma, and hypoxia response, angiogenesis, and enhanced coagulation in IS. In this RBPS, *SMAD9* was found to be associated with dementia; *POLR2F* and *ERI1* were identified to be associated with aging. Under hypoxia, *Irf5*/*Trim21* in microglia and *Taf7*/*Trim21* in pericytes were identified as RBPS-related networks. There are some limitations to this study. The gene signature was developed based on large publicly available databases and retrospective cohorts. However, no independent clinical cohort in local hospitals for validation was evaluated. In addition, the properties of these RBP genes need to be verified at cellular levels and using animal models. With the identification of new RBP molecules, computational biological analyses need to be updated to identify important molecules in the occurrence and development of glioma and IS.

## Conclusion

In conclusion, we developed a 6-RBP gene signature associated with a glioma prognosis and an IS diagnosis. In addition, an automatic machine learning classification model based on radiomics features from MRI was developed to stratify the RBPS risk. The RBPS was associated with immunosuppression, energy metabolism, and enhanced tumor growth in glioma, and hypoxia response, angiogenesis, and increased coagulation in IS. Upregulation of *SMAD9* was associated with dementia, while downregulation of *POLR2F* was associated with aging-related hypoxic stress. The RBPS is expected to serve as a biomarker to study the common mechanism between glioma and IS. These six RBP gene markers play a critical role in the association of IS with glioma, as revealed by our study.

## Data availability statement

The original contributions presented in the study are included in the article/Supplementary material, further inquiries can be directed to the corresponding author/s.

## Author contributions

WL conceptualized the data, involved in formal analysis and methodology, investigated the study, and wrote the original draft. QW, YC, NW, QN, CQ, and QW participated in literature review, data collection, and statistical analysis. YZ conceptualized the study, supervised the data, and reviewed and edited the manuscript. All the authors read and approved the final manuscript.

## Funding

This study was funded by Provincial Key R&D Program, Science and Technology Department of Zhejiang Province (Grant No. 2017C03018), Key Program of Administration of Traditional Chinese Medicine, Zhejiang Province (Grant No. 2018ZZ015), Nursery Project of the Affiliated Tai'an City Central Hospital of Qingdao University (Grant No. 2022M PM06), Shandong Medical and Health Technology Development Fund (Grant No.202103070325), and 2021 Zhejiang Normal University Interdisciplinary Advance Research Fund.

## Conflict of interest

The authors declare that the research was conducted in the absence of any commercial or financial relationships that could be construed as a potential conflict of interest.

## Publisher's note

All claims expressed in this article are solely those of the authors and do not necessarily represent those of their affiliated organizations, or those of the publisher, the editors and the reviewers. Any product that may be evaluated in this article, or claim that may be made by its manufacturer, is not guaranteed or endorsed by the publisher.

## Abbreviations

TCGA: The cancer genome atlas
CGGA: Chinese glioma genome atlas
LGG: Lower-grade gliomas
GBM: Glioblastomas
RBP: RNA-binding protein
LASSO: Least absolute shrinkage and selection operator
GO: Gene ontology
GSEA: Gene set enrichment analysis
IDH: Isocitrate dehydrogenases
MGMT: O6-methylguanine-DNA methyltransferase
IS: Ischemic stroke.

## References

[B1] Acs B. Rantalainen M. Hartman J. (2020). Artificial intelligence as the next step towards precision pathology. J. Intern. Med. 288, 62–81. 10.1111/joim.13030 32128929

[B2] Aibar S. González-Blas C. B. Moerman T. Huynh-Thu V. A. Imrichova H. Hulselmans G.. (2017). SCENIC: single-cell regulatory network inference and clustering. Nat. Methods 14, 1083–1086. 10.1038/nmeth.4463 28991892 PMC5937676

[B3] Al Mamun A. Chauhan A. Qi S. Ngwa C. Xu Y. Sharmeen R.. (2020). Microglial IRF5-IRF4 regulatory axis regulates neuroinflammation after cerebral ischemia and impacts stroke outcomes. Proc. Natl. Acad. Sci. USA 117, 1742–1752. 10.1073/pnas.1914742117 31892541 PMC6983422

[B4] Al Mamun A. Chauhan A. Yu H. Xu Y. Sharmeen R. Liu F.. (2018). Interferon regulatory factor 4/5 signaling impacts on microglial activation after ischemic stroke in mice. Eur. J. Neurosci. 47, 140–149. 10.1111/ejn.13778 29131464 PMC5771847

[B5] Al Mamun A. Yu H. Sharmeen R. McCullough L. D. Liu F. (2021). IRF5 signaling in phagocytes is detrimental to neonatal hypoxic ischemic encephalopathy. Transl. Stroke Res. 12, 602–614. 10.1007/s12975-020-00832-x 32761315 PMC7862420

[B6] Ardelt A. A. Carpenter R. S. Iwuchukwu I. Zhang A. Lin W. Kosciuczuk E.. (2017). Transgenic expression of HuR increases vasogenic edema and impedes functional recovery in rodent ischemic stroke. Neurosci. Lett. 661, 126–131. 10.1016/j.neulet.2017.09.062 28982595 PMC5722242

[B7] Armstrong N. J. Mather K. A. Sargurupremraj M. Knol M. J. Malik R. Satizabal C. L.. (2020). Common genetic variation indicates separate causes for periventricular and deep white matter hyperintensities. Stroke 51, 2111–2121. 10.1161/STROKEAHA.119.027544 32517579 PMC7365038

[B8] Armulik A. Genové G. Betsholtz C. (2011). Pericytes: developmental, physiological, and pathological perspectives, problems, and promises. Dev. Cell 21, 193–215. 10.1016/j.devcel.2011.07.001 21839917

[B9] Armulik A. Genové G. Mäe M. Nisancioglu M. H. Wallgard E. Niaudet C.. (2010). Pericytes regulate the blood-brain barrier. Nature 468, 557–561. 10.1038/nature09522 20944627

[B10] Bai J. Yang B. Shi R. Shao X. Yang Y. Wang F.. (2020). Could microtubule inhibitors be the best choice of therapy in gastric cancer with high immune activity: mutant DYNC1H1 as a biomarker. Aging (Albany, NY) 12, 25101–25119. 10.18632/aging.104084 33221769 PMC7803585

[B11] Bakas S. Akbari H. Sotiras A. Bilello M. Rozycki M. Kirby J. S.. (2017). Advancing the Cancer Genome Atlas glioma MRI collections with expert segmentation labels and radiomic features. Sci. Data 4, 170117. 10.1038/sdata.2017.117 28872634 PMC5685212

[B12] Barbagallo D. Caponnetto A. Cirnigliaro M. Brex D. Barbagallo C. D'Angeli F.. (2018). CircSMARCA5 inhibits migration of glioblastoma multiforme cells by regulating a molecular axis involving splicing factors SRSF1/SRSF3/PTB. Int. J. Mol. Sci. 19, 480. 10.3390/ijms19020480 29415469 PMC5855702

[B13] Barr T. L. Conley Y. Ding J. Dillman A. Warach S. Singleton A.. (2010). Genomic biomarkers and cellular pathways of ischemic stroke by RNA gene expression profiling. Neurology 75, 1009–1014. 10.1212/WNL.0b013e3181f2b37f 20837969 PMC2942033

[B14] Barsoum I. B. Smallwood C. A. Siemens D. R. Graham C. H. (2014). A mechanism of hypoxia-mediated escape from adaptive immunity in cancer cells. Cancer Res. 74, 665–674. 10.1158/0008-5472.CAN-13-0992 24336068

[B15] Becht E. McInnes L. Healy J. Dutertre C.-A. Kwok I. W. H. Ng L. G.. (2019). Dimensionality reduction for visualizing single-cell data using UMAP. Nat. Biotechnol. 37, 38–44. 10.1038/nbt.4314 30531897

[B16] Beers A. Gerstner E. Rosen B. Clunie D. Pieper S. Fedorov A.. (2018). Dicom-seg conversions for TCGA-LGG, and TCGA-GBM segmentation datasets. Cancer Imaging Arch. 10.7937/TCIA.2018.ow6ce3ml

[B17] Benjamin E. J. Muntner P. Alonso A. Bittencourt M. S. Callaway C. W. Carson A. P.. (2019). Heart disease and stroke statistics-2019 update: a report from the American Heart Association. Circulation 139, e56–e528. 10.1161/CIR.0000000000000659 30700139

[B18] Bhargava S. Patil V. Mahalingam K. Somasundaram K. (2017). Elucidation of the genetic and epigenetic landscape alterations in RNA binding proteins in glioblastoma. Oncotarget 8, 16650–16668. 10.18632/oncotarget.14287 28035070 PMC5369992

[B19] Bi J. Chowdhry S. Wu S. Zhang W. Masui K. Mischel P. S.. (2020). Altered cellular metabolism in gliomas—an emerging landscape of actionable co-dependency targets. Nat. Rev. Cancer 20, 57–70. 10.1038/s41568-019-0226-5 31806884

[B20] Blanc E. M. Bruce-Keller A. J. Mattson M. P. (1998). Astrocytic gap junctional communication decreases neuronal vulnerability to oxidative stress-induced disruption of Ca^2+^ homeostasis and cell death. J. Neurochem. 70, 958–970. 10.1046/j.1471-4159.1998.70030958.x 9489715

[B21] Blanche P. Dartigues J.-F. Jacqmin-Gadda H. (2013). Estimating and comparing time-dependent areas under receiver operating characteristic curves for censored event times with competing risks. Statist. Med. 32, 5381–5397. 10.1002/sim.5958 24027076

[B22] Boucas J. Fritz C. Schmitt A. Riabinska A. Thelen L. Peifer M.. (2015). Label-free protein-RNA interactome analysis identifies khsrp signaling downstream of the p38/Mk2 kinase complex as a critical modulator of cell cycle progression. PLoS ONE 10, e0125745. 10.1371/journal.pone.0125745 25993413 PMC4439058

[B23] Brauner S. Jiang X. Thorlacius G. E. Lundberg A. M. Östberg T. Yan Z.-Q.. (2018). Augmented Th17 differentiation in Trim21 deficiency promotes a stable phenotype of atherosclerotic plaques with high collagen content. Cardiovasc. Res. 114, 158–167. 10.1093/cvr/cvx181 29016728

[B24] Cao J. Spielmann M. Qiu X. Huang X. Ibrahim D. M. Hill A. J.. (2019). The single-cell transcriptional landscape of mammalian organogenesis. Nature 566, 496–502. 10.1038/s41586-019-0969-x 30787437 PMC6434952

[B25] Cestari D. M. Weine D. M. Panageas K. S. Segal A. Z. Deangelis L. M. (2004). Stroke in patients with cancer: incidence and etiology. Neurology 62, 2025. 10.1212/01.WNL.0000129912.56486.2B 15184609

[B26] Chen C.-W. Cheng T.-J. Ho C.-H. Wang J.-J. Weng S.-F. Hou Y.-C.. (2017). Increased risk of brain cancer incidence in stroke patients: a clinical case series, population-based and longitudinal follow-up study. Oncotarget 8, 108989–108999. 10.18632/oncotarget.22480 29312585 PMC5752498

[B27] Chen X. Chen H. Xu M. Shen J. (2013). Targeting reactive nitrogen species: a promising therapeutic strategy for cerebral ischemia-reperfusion injury. Acta Pharmacol. Sin. 34, 67–77. 10.1038/aps.2012.82 22842734 PMC4086503

[B28] Cheng J. Korte N. Nortley R. Sethi H. Tang Y. Attwell D.. (2018). Targeting pericytes for therapeutic approaches to neurological disorders. Acta Neuropathol. 136, 507–523. 10.1007/s00401-018-1893-0 30097696 PMC6132947

[B29] Cheng L. Huang Z. Zhou W. Wu Q. Donnola S. Liu J. K.. (2013). Glioblastoma stem cells generate vascular pericytes to support vessel function and tumor growth. Cell 153, 139–152. 10.1016/j.cell.2013.02.021 23540695 PMC3638263

[B30] Clark K. Vendt B. Smith K. Freymann J. Kirby J. Koppel P.. (2013). The Cancer Imaging Archive (TCIA): maintaining and operating a public information repository. J. Digit. Imaging 26, 1045–1057. 10.1007/s10278-013-9622-7 23884657 PMC3824915

[B31] Daneman R. Zhou L. Kebede A. A. Barres B. A. (2010). Pericytes are required for blood-brain barrier integrity during embryogenesis. Nature 468, 562–566. 10.1038/nature09513 20944625 PMC3241506

[B32] Dang H. Takai A. Forgues M. Pomyen Y. Mou H. Xue W.. (2017). Oncogenic activation of the RNA binding protein NELFE and MYC signaling in hepatocellular carcinoma. Cancer Cell. 32, 101–114.e8. 10.1016/j.ccell.2017.06.002 28697339 PMC5539779

[B33] Declercq M. Biquand E. Karim M. Pietrosemoli N. Jacob Y. Demeret C.. (2020). Influenza A virus co-opts ERI1 exonuclease bound to histone mRNA to promote viral transcription. Nucleic Acids Res. 48, 10428–10440. 10.1093/nar/gkaa771 32960265 PMC7544206

[B34] Duan Y. Zhang D. (2020). Identification of novel prognostic alternative splicing signature in papillary renal cell carcinoma. J. Cell. Biochem. 121, 672–689. 10.1002/jcb.29314 31407370

[B35] Eguchi J. Kong X. Tenta M. Wang X. Kang S. Rosen E. D.. (2013). Interferon regulatory factor 4 regulates obesity-induced inflammation through regulation of adipose tissue macrophage polarization. Diabetes 62, 3394–3403. 10.2337/db12-1327 23835343 PMC3781469

[B36] Erdem-Eraslan L. van den Bent M. J. Hoogstrate Y. Naz-Khan H. Stubbs A. van der Spek P.. (2016). Identification of patients with recurrent glioblastoma who may benefit from combined bevacizumab and CCNU therapy: a report from the BELOB trial. Cancer Res. 76, 525–534. 10.1158/0008-5472.CAN-15-0776 26762204

[B37] Fan Z. Zhao S. Zhu Y. Li Z. Liu Z. Yan Y.. (2020). Interferon regulatory factor 5 mediates lipopolysaccharide-induced neuroinflammation. Front. Immunol. 11, 600479. 10.3389/fimmu.2020.600479 33362784 PMC7755991

[B38] Fang Z. Wu D. Deng J. Yang Q. Zhang X. Chen J.. (2021). An MD2-perturbing peptide has therapeutic effects in rodent and rhesus monkey models of stroke. Sci. Transl. Med. 13:eabb6716. 10.1126/scitranslmed.abb6716 34108252

[B39] Farkas A. Schlakman B. Khan M. Joyner D. (2018). Glioblastoma presenting with acute middle cerebral artery territory infarct. J. Stroke Cerebrovasc. Dis. 27, e113–e114. 10.1016/j.jstrokecerebrovasdis.2018.01.019 29472157

[B40] Fraum T. J. Kreisl T. N. Sul J. Fine H. A. Iwamoto F. M. (2011). Ischemic stroke and intracranial hemorrhage in glioma patients on antiangiogenic therapy. J. Neurooncol. 105, 281–289. 10.1007/s11060-011-0579-4 21603965 PMC3168718

[B41] Furukawa T. Kuboki Y. Tanji E. Yoshida S. Hatori T. Yamamoto M.. (2011). Whole-exome sequencing uncovers frequent GNAS mutations in intraductal papillary mucinous neoplasms of the pancreas. Sci. Rep. 1, 161. 10.1038/srep00161 22355676 PMC3240977

[B42] Gabrilovich D. I. Chen H. L. Girgis K. R. Cunningham H. T. Meny G. M. Nadaf S.. (1996). Production of vascular endothelial growth factor by human tumors inhibits the functional maturation of dendritic cells. Nat. Med. 2, 1096–1103. 10.1038/nm1096-1096 8837607

[B43] Gerstberger S. Hafner M. Tuschl T. A. (2014). census of human RNA-binding proteins. Nat. Rev. Genet. 15, 829–845. 10.1038/nrg3813 25365966 PMC11148870

[B44] Ghosh M. K. Chakraborty D. Sarkar S. Bhowmik A. Basu M. (2019). The interrelationship between cerebral ischemic stroke and glioma: a comprehensive study of recent reports. Signal Transduct Target Ther. 4, 42. 10.1038/s41392-019-0075-4 31637020 PMC6799849

[B45] Goldman M. J. Craft B. Hastie M. Repečka K. McDade F. Kamath A.. (2020). Visualizing and interpreting cancer genomics data via the Xena platform. Nat. Biotechnol. 38, 675–678. 10.1038/s41587-020-0546-8 32444850 PMC7386072

[B46] Gong L.-B. Wen T. Li Z. Xin X. Che X.-F. Wang J.. (2019). DYNC1I1 promotes the proliferation and migration of gastric cancer by up-regulating IL-6 expression. Front. Oncol. (2019). 9:491. 10.3389/fonc.2019.00491 31249807 PMC6582752

[B47] Göritz C. Dias D. O. Tomilin N. Barbacid M. Shupliakov O. Frisén J.. (2011). A pericyte origin of spinal cord scar tissue. Science. 333, 238–242. 10.1126/science.1203165 21737741

[B48] Graus F. Rogers L. R. Posner J. B. (1985). Cerebrovascular complications in patients with cancer. Medicine (Baltimore) 64, 16–35. 10.1097/00005792-198501000-00002 3965856

[B49] Hall C. N. Reynell C. Gesslein B. Hamilton N. B. Mishra A. Sutherland B. A.. (2014). Capillary pericytes regulate cerebral blood flow in health and disease. Nature 508, 55–60. 10.1038/nature13165 24670647 PMC3976267

[B50] Hartmann D. A. Hyacinth H. I. Liao F. Shih A. Y. (2018). Does pathology of small venules contribute to cerebral microinfarcts and dementia? J. Neurochem. 144, 517–526. 10.1111/jnc.14228 28950410 PMC5869083

[B51] Heng J. S. Rattner A. Stein-O'Brien G. L. Winer B. L. Jones B. W. Vernon H. J.. (2019). Hypoxia tolerance in the Norrin-deficient retina and the chronically hypoxic brain studied at single-cell resolution. Proc. Natl. Acad. Sci. USA 116, 9103–9114. 10.1073/pnas.1821122116 30988181 PMC6500147

[B52] Hoang H. T. Schlager M. A. Carter A. P. Bullock S. L. (2017). DYNC1H1 mutations associated with neurological diseases compromise processivity of dynein-dynactin-cargo adaptor complexes. Proc. Natl. Acad. Sci. USA 114, E1597–E1606. 10.1073/pnas.1620141114 28196890 PMC5338514

[B53] Hokama M. Oka S. Leon J. Ninomiya T. Honda H. Sasaki K.. (2014). Altered expression of diabetes-related genes in Alzheimer's disease brains: the Hisayama study. Cereb Cortex 24, 2476–2488. 10.1093/cercor/bht101 23595620 PMC4128707

[B54] Huang H.-L. Yao H.-S. Wang Y. Wang W.-J. Hu Z.-Q. Jin K.-Z.. (2014). Proteomic identification of tumor biomarkers associated with primary gallbladder cancer. World J. Gastroenterol. 20, 5511–5518. 10.3748/wjg.v20.i18.5511 24833881 PMC4017066

[B55] Iglesia M. D. Parker J. S. Hoadley K. A. Serody J. S. Perou C. M. Vincent B. G.. (2016). Genomic analysis of immune cell infiltrates across 11 tumor types. J. Natl. Cancer Inst. 108:djw144. 10.1093/jnci/djw144 27335052 PMC5241901

[B56] Jackson S. ElAli A. Virgintino D. Gilbert M. R. (2017). Blood-brain barrier pericyte importance in malignant gliomas: what we can learn from stroke and Alzheimer's disease. Neuro-oncology 19, 1173–1182. 10.1093/neuonc/nox058 28541444 PMC5570196

[B57] Kafasla P. Skliris A. Kontoyiannis D. L. (2014). Post-transcriptional coordination of immunological responses by RNA-binding proteins. Nat. Immunol. 15, 492–502. 10.1038/ni.2884 24840980

[B58] Kallergi G. Markomanolaki H. Giannoukaraki V. Papadaki M. A. Strati A. Lianidou E. S.. (2009). Hypoxia-inducible factor-1alpha and vascular endothelial growth factor expression in circulating tumor cells of breast cancer patients. Breast Cancer Res. 11, R84. 10.1186/bcr2452 19919679 PMC2815547

[B59] Kalogeris T. Bao Y. Korthuis R. J. (2014). Mitochondrial reactive oxygen species: a double edged sword in ischemia/reperfusion vs preconditioning. Redox Biol. 2, 702–714. 10.1016/j.redox.2014.05.006 24944913 PMC4060303

[B60] Kasivisvanathan V. Shalhoub J. Lim C. S. Shepherd A. C. Thapar A. Davies A. H.. (2011). Hypoxia-inducible factor-1 in arterial disease: a putative therapeutic target. Curr. Vasc. Pharmacol. 9, 333–349. 10.2174/157016111795495602 20807188

[B61] Kedde M. Strasser M. J. Boldajipour B. Vrielink J. A. F. O. Slanchev K. le Sage C. Nagel R. (2007). RNA-binding protein Dnd1 inhibits microRNA access to target mRNA. Cell 131, 1273–1286. 10.1016/j.cell.2007.11.034 18155131

[B62] Khasraw M. Reardon D. A. Weller M. Sampson J. H. (2020). PD-1 inhibitors: do they have a future in the treatment of glioblastoma? Clin. Cancer Res. 26, 5287–5296. 10.1158/1078-0432.CCR-20-1135 32527943 PMC7682636

[B63] Kikuno M. Ueno Y. Takekawa H. Kanemaru K. Shimizu T. Kuriki A.. (2021). Distinction in prevalence of atherosclerotic embolic sources in cryptogenic stroke with cancer status. J. Am. Heart Assoc. 10, e021375. 10.1161/JAHA.120.021375 34689573 PMC8751843

[B64] Kim S.-J. Ju J.-S. Kang M.-H. Won J. E. Kim Y. H. Raninga P. V.. (2020). RNA-binding protein NONO contributes to cancer cell growth and confers drug resistance as a theranostic target in TNBC. Theranostics 10, 7974–7992. 10.7150/thno.45037 32724453 PMC7381744

[B65] Korte N. Ilkan Z. Pearson C. L. Pfeiffer T. Singhal P. Rock J. R.. (2022). The Ca2+-gated channel TMEM16A amplifies capillary pericyte contraction and reduces cerebral blood flow after ischemia. J. Clin. Invest. 2022, e154118. 10.1101/2022.02.03.479031 35316222 PMC9057602

[B66] Kreisl T. N. Toothaker T. Karimi S. DeAngelis L. M. (2008). Ischemic stroke in patients with primary brain tumors. Neurology 70, 2314–2320. 10.1212/01.wnl.0000314648.82924.6f 18541885

[B67] Lambertsen K. L. Finsen B. Clausen B. H. (2019). Post-stroke inflammation-target or tool for therapy? Acta Neuropathol. 137, 693–714. 10.1007/s00401-018-1930-z 30483945 PMC6482288

[B68] Lan Y. Lou J. Hu J. Yu Z. Lyu W. Zhang B.. (2020). Downregulation of SNRPG induces cell cycle arrest and sensitizes human glioblastoma cells to temozolomide by targeting Myc through a p53-dependent signaling pathway. Cancer Biol. Med. 17, 112–131. 10.20892/j.issn.2095-3941.2019.0164 32296580 PMC7142844

[B69] Le T. T. Fu W. Moore J. H. (2020). Scaling tree-based automated machine learning to biomedical big data with a feature set selector. Bioinformatics 36, 250–256. 10.1093/bioinformatics/btz470 31165141 PMC6956793

[B70] Leek J. T. Johnson W. E. Parker H. S. Jaffe A. E. Storey J. D. (2012). The sva package for removing batch effects and other unwanted variation in high-throughput experiments. Bioinformatics 28, 882–883. 10.1093/bioinformatics/bts034 22257669 PMC3307112

[B71] Li W. Li X. Gao L.-N. You C.-G. (2020). Integrated analysis of the functions and prognostic values of RNA binding proteins in lung squamous cell carcinoma. Front. Genet. 11, 185. 10.3389/fgene.2020.00185 32194639 PMC7066120

[B72] Liang Y. Hannan R. Fu Y.-X. (2021). Type I IFN activating type I dendritic cells for antitumor immunity. Clin. Cancer Res. 27, 3818–3824. 10.1158/1078-0432.CCR-20-2564 33692027

[B73] Liao J.-Y. Yang B. Zhang Y.-C. Wang X.-J. Ye Y. Peng J.-W.. (2020). EuRBPDB: a comprehensive resource for annotation, functional and oncological investigation of eukaryotic RNA binding proteins (RBPs). Nucleic Acids Res. 48, D307–D313. 10.1093/nar/gkz823 31598693 PMC6943034

[B74] Liberzon A. Birger C. Thorvaldsdóttir H. Ghandi M. Mesirov J. Tamayo P.. (2015). The Molecular Signatures Database (MSigDB) hallmark gene set collection. Cell Syst. 1, 417–425. 10.1016/j.cels.2015.12.004 26771021 PMC4707969

[B75] Liberzon A. Subramanian A. Pinchback R. Thorvaldsdottir H. Tamayo P. Mesirov J. P.. (2011). Molecular signatures database (MSigDB) 3.0. Bioinformatics 27, 1739–1740. 10.1093/bioinformatics/btr260 21546393 PMC3106198

[B76] Lin W. Wang Y. Chen Y. Wang Q. Gu Z. Zhu Y.. (2021). Role of calcium signaling pathway-related gene regulatory networks in ischemic stroke based on multiple WGCNA and single-cell analysis. Oxid. Med. Cell. Longev. 2021, 1–35. 10.1155/2021/8060477 34987704 PMC8720592

[B77] Liu W. Xu Z. Zhou J. Xing S. Li Z. Gao X.. (2020). High levels of HIST1H2BK in low-grade glioma predicts poor prognosis: a study using CGGA and TCGA data. Front. Oncol. 10, 627. 10.3389/fonc.2020.00627 32457836 PMC7225299

[B78] Liu Z. Chopp M. (2016). Astrocytes, therapeutic targets for neuroprotection and neurorestoration in ischemic stroke. Prog. Neurobiol. 144, 103–120. 10.1016/j.pneurobio.2015.09.008 26455456 PMC4826643

[B79] Malta T. M. Sokolov A. Gentles A. J. Burzykowski T. Poisson L. Weinstein J. N.. (2018). Machine learning identifies stemness features associated with oncogenic dedifferentiation. Cell 173, 338–354.e15. 10.1016/j.cell.2018.03.034 29625051 PMC5902191

[B80] Mancino A. Schioppa T. Larghi P. Pasqualini F. Nebuloni M. Chen I.-H.. (2008). Divergent effects of hypoxia on dendritic cell functions. Blood 112, 3723–3734. 10.1182/blood-2008-02-142091 18694997

[B81] Mantovani A. Sozzani S. Locati M. Allavena P. Sica A. (2002). Macrophage polarization: tumor-associated macrophages as a paradigm for polarized M2 mononuclear phagocytes. Trends Immunol. 23, 549–555. 10.1016/S1471-4906(02)02302-5 12401408

[B82] Mohibi S. Chen X. Zhang J. (2019). Cancer the'RBP'eutics-RNA-binding proteins as therapeutic targets for cancer. Pharmacol. Therap. 203, 107390. 10.1016/j.pharmthera.2019.07.001 31302171 PMC6848768

[B83] Musuka T. D. Wilton S. B. Traboulsi M. Hill M. D. (2015). Diagnosis and management of acute ischemic stroke: speed is critical. CMAJ Can. Med. Assoc. J. 2015, 887. 10.1503/cmaj.140355 26243819 PMC4562827

[B84] Nakagomi T. Kubo S. Nakano-Doi A. Sakuma R. Lu S. Narita A.. (2015). Brain vascular pericytes following ischemia have multipotential stem cell activity to differentiate into neural and vascular lineage cells. Stem Cells 33, 1962–1974. 10.1002/stem.1977 25694098

[B85] Newman A. M. Steen C. B. Liu C. L. Gentles A. J. Chaudhuri A. A. Scherer F.. (2019). Determining cell type abundance and expression from bulk tissues with digital cytometry. Nat. Biotechnol. 37, 773–782. 10.1038/s41587-019-0114-2 31061481 PMC6610714

[B86] Ngeow J. Yu W. Yehia L. Niazi F. Chen J. Tang X.. (2015). Exome sequencing reveals germline SMAD9 mutation that reduces phosphatase and tensin homolog expression and is associated with hamartomatous polyposis and gastrointestinal ganglioneuromas. Gastroenterology. 149, 886–889.e5. 10.1053/j.gastro.2015.06.027 26122142

[B87] Noda M. Inaji M. Karakama J. Arai Y. Kuroha M. Tamura K.. (2022). Ischemic stroke with multiple cerebral artery stenosis in a patient with an anaplastic astrocytoma during bevacizumab treatment: a case report. NMC Case Rep. J. 9, 13–17. 10.2176/jns-nmc.2021-0297 35340332 PMC8906840

[B88] Noman M. Z. Desantis G. Janji B. Hasmim M. Karray S. Dessen P.. (2014). PD-L1 is a novel direct target of HIF-1α, and its blockade under hypoxia enhanced MDSC-mediated T cell activation. J. Exp. Med. 211, 781–790. 10.1084/jem.20131916 24778419 PMC4010891

[B89] Noman M. Z. Hasmim M. Messai Y. Terry S. Kieda C. Janji B.. (2015). Hypoxia: a key player in antitumor immune response. A review in the theme: cellular responses to hypoxia. Am. J. Physiol. Cell Physiol. 309, C569–C579. 10.1152/ajpcell.00207.2015 26310815 PMC4628936

[B90] O'Connell G. C. Petrone A. B. Treadway M. B. Tennant C. S. Lucke-Wold N. Chantler P. D.. (2016). Machine-learning approach identifies a pattern of gene expression in peripheral blood that can accurately detect ischaemic stroke. NPJ Genomic Med. 1, 16038. 10.1038/npjgenmed.2016.38 29263821 PMC5685316

[B91] O'Connell G. C. Treadway M. B. Petrone A. B. Tennant C. S. Lucke-Wold N. Chantler P. D.. (2017). Peripheral blood AKAP7 expression as an early marker for lymphocyte-mediated post-stroke blood brain barrier disruption. Sci. Rep. 7, 1172. 10.1038/s41598-017-01178-5 28446746 PMC5430856

[B92] Ohta A. Diwanji R. Kini R. Subramanian M. Ohta A. Sitkovsky M.. (2011). In vivo T cell activation in lymphoid tissues is inhibited in the oxygen-poor microenvironment. Front. Immunol. 2, 27. 10.3389/fimmu.2011.00027 22566817 PMC3342240

[B93] Ostrom Q. T. Gittleman H. Farah P. Ondracek A. Chen Y. Wolinsky Y.. (2013). Statistical report: primary brain and central nervous system tumors diagnosed in the United States in 2006–2010. Neuro-Oncology 15, ii1–ii56. 10.1093/neuonc/not151 24137015 PMC3798196

[B94] Özen I. Deierborg T. Miharada K. Padel T. Englund E. Genové G.. (2014). Brain pericytes acquire a microglial phenotype after stroke. Acta Neuropathol. 128, 381–396. 10.1007/s00401-014-1295-x 24848101 PMC4131168

[B95] Pan J.-A. Sun Y. Jiang Y.-P. Bott A. J. Jaber N. Dou Z.. (2016). TRIM21 ubiquitylates SQSTM1/p62 and suppresses protein sequestration to regulate redox homeostasis. Mol. Cell 61, 720–733. 10.1016/j.molcel.2016.02.007 26942676 PMC4779181

[B96] Paun A. Reinert J. T. Jiang Z. Medin C. Balkhi M. Y. Fitzgerald K. A.. (2008). Functional characterization of murine interferon regulatory factor 5 (IRF-5) and its role in the innate antiviral response. J. Biol. Chem. 283, 14295–14308. 10.1074/jbc.M800501200 18332133 PMC2386920

[B97] Phipps M. S. Cronin C. A. (2020). Management of acute ischemic stroke. BMJ 368, l6983. 10.1136/bmj.l6983 32054610

[B98] Qi Y. Liu B. Sun Q. Xiong X. Chen Q. (2020). Immune checkpoint targeted therapy in glioma: status and hopes. Front. Immunol. 11, 578877. 10.3389/fimmu.2020.578877 33329549 PMC7729019

[B99] Qin H. Ni H. Liu Y. Yuan Y. Xi T. Li X.. (2020). RNA-binding proteins in tumor progression. J. Hematol. Oncol. 13, 90. 10.1186/s13045-020-00927-w 32653017 PMC7353687

[B100] Qiu X. Hill A. Packer J. Lin D. Ma Y.-A. Trapnell C.. (2017a). Single-cell mRNA quantification and differential analysis with Census. Nat. Methods 14, 309–315. 10.1038/nmeth.4150 28114287 PMC5330805

[B101] Qiu X. Mao Q. Tang Y. Wang L. Chawla R. Pliner H.. (2017b). Reversed graph embedding resolves complex single-cell developmental trajectories. Genomics. 14, 979–982. 10.1101/110668 28825705 PMC5764547

[B102] Qureshi A. I. Malik A. A. Saeed O. Adil M. M. Rodriguez G. J. Suri M. F. K.. (2015). Incident cancer in a cohort of 3,247 cancer diagnosis free ischemic stroke patients. Cerebrovasc. Dis. 39, 262–268. 10.1159/000375154 25871304

[B103] Ransohoff R. M. A. (2016). polarizing question: do M1 and M2 microglia exist? Nat. Neurosci. 19, 987–991. 10.1038/nn.4338 27459405

[B104] Ransom B. R. Ransom C. B. (2012). Astrocytes: multitalented stars of the central nervous system. Methods Mol. Biol. 814, 3–7. 10.1007/978-1-61779-452-0_1 22144296

[B105] Rasmussen R. D. Gajjar M. K. Tuckova L. Jensen K. E. Maya-Mendoza A. Holst C. B.. (2016). BRCA1-regulated RRM2 expression protects glioblastoma cells from endogenous replication stress and promotes tumorigenicity. Nat. Commun. 7, 13398. 10.1038/ncomms13398 27845331 PMC5116074

[B106] Reinhold W. C. Sunshine M. Liu H. Varma S. Kohn K. W. Morris J.. (2012). CellMiner: a web-based suite of genomic and pharmacologic tools to explore transcript and drug patterns in the NCI-60 cell line set. Cancer Res. 72, 3499–3511. 10.1158/0008-5472.CAN-12-1370 22802077 PMC3399763

[B107] Ritchie M. E. Phipson B. Wu D. Hu Y. Law C. W. Shi W.. (2015). limma powers differential expression analyses for RNA-sequencing and microarray studies. Nucleic Acids Res. 43, e47–e47. 10.1093/nar/gkv007 25605792 PMC4402510

[B108] Segura-Collar B. Garranzo-Asensio M. Herranz B. Hernández-SanMiguel E. Cejalvo T. Casas B. S.. (2021). Tumor-derived pericytes driven by EGFR mutations govern the vascular and immune microenvironment of gliomas. Cancer Res. 81, 2142–2156. 10.1158/0008-5472.CAN-20-3558 33593822

[B109] Seidel C. Hentschel B. Simon M. Schnell O. Heese O. Tatagiba M.. (2013). A comprehensive analysis of vascular complications in 3,889 glioma patients from the German Glioma Network. J. Neurol. 260, 847–855. 10.1007/s00415-012-6718-9 23104124

[B110] Semenza G. L. (2012). Hypoxia-inducible factors: mediators of cancer progression and targets for cancer therapy. Trends Pharmacol. Sci. 33, 207–214. 10.1016/j.tips.2012.01.005 22398146 PMC3437546

[B111] Shao J. Zhang J. Zhang Z. Jiang H. Lou X. Huang B.. (2013). Alternative polyadenylation in glioblastoma multiforme and changes in predicted RNA binding protein profiles. OMICS J Integr. Biol. 17, 136–149. 10.1089/omi.2012.0098 23421905 PMC3603499

[B112] Sharma A. Brenner M. Jacob A. Marambaud P. Wang P. (2021). Extracellular CIRP activates the IL-6Rα/STAT3/Cdk5 pathway in neurons. Mol. Neurobiol. 58, 3628–3640. 10.1007/s12035-021-02368-z 33783711 PMC10404139

[B113] Si W. Li Z. Huang Z. Ye S. Li X. Li Y.. (2020). Binding protein Motif 3 inhibits oxygen-glucose deprivation/reoxygenation-induced apoptosis through promoting stress granules formation in PC12 cells and rat primary cortical neurons. Front. Cell. Neurosci. 14, 559384. 10.3389/fncel.2020.559384 32982696 PMC7492797

[B114] Sjöstrand M. Ambrosi A. Brauner S. Sullivan J. Malin S. Kuchroo V. K.. (2013). Expression of the immune regulator tripartite-motif 21 is controlled by IFN regulatory factors. JI 191, 3753–3763. 10.4049/jimmunol.1202341 23975864

[B115] Søndergaard K. L. Hilton D. A. Penney M. Ollerenshaw M. Demaine A. G. (2002). Expression of hypoxia-inducible factor 1alpha in tumours of patients with glioblastoma. Neuropathol. Appl. Neurobiol. 28, 210–217. 10.1046/j.1365-2990.2002.00391.x 12060345

[B116] Stark K. Eckart A. Haidari S. Tirniceriu A. Lorenz M. von Brühl M.-L.. (2013). Capillary and arteriolar pericytes attract innate leukocytes exiting through venules and “instruct” them with pattern-recognition and motility programs. Nat. Immunol. 14, 41–51. 10.1038/ni.2477 23179077

[B117] Stuart T. Butler A. Hoffman P. Hafemeister C. Papalexi E. Mauck W. M.. (2019). Comprehensive integration of single-cell data. Cell. 177, 1888–1902.e21. 10.1016/j.cell.2019.05.031 31178118 PMC6687398

[B118] Su X. Chen N. Sun H. Liu Y. Yang X. Wang W.. (2019). Automated machine learning based on radiomics features predicts H3 K27M mutation in midline gliomas of the brain. Neuro-Oncology. 2019:noz184. 10.1093/neuonc/noz184 31563963 PMC7442326

[B119] Subramanian A. Tamayo P. Mootha V. K. Mukherjee S. Ebert B. L. Gillette M. A.. (2005). Gene set enrichment analysis: a knowledgE-based approach for interpreting genome-wide expression profiles. Proc. Natl. Acad. Sci. USA. 102, 15545–15550. 10.1073/pnas.0506580102 16199517 PMC1239896

[B120] Sumbayev V. V. Yasinska I. M. (2007). Mechanisms of hypoxic signal transduction regulated by reactive nitrogen species. Scand. J. Immunol. 65, 399–406. 10.1111/j.1365-3083.2007.01919.x 17444949

[B121] Sun P. Li Y. Chao X. Li J. Luo R. Li M.. (2020). Clinical characteristics and prognostic implications of BRCA-associated tumors in males: a pan-tumor survey. BMC Cancer 20, 994. 10.1186/s12885-020-07481-1 33054725 PMC7556962

[B122] Svensson A. Özen I. Genové G. Paul G. Bengzon J. (2015). Endogenous brain pericytes are widely activated and contribute to mouse glioma microvasculature. PLoS ONE 10, e0123553. 10.1371/journal.pone.0123553 25875288 PMC4395339

[B123] Tanislav C. Adarkwah C. C. Jakob L. Kostev K. (2019). Increased risk for cancer after stroke at a young age: etiological relevance or incidental finding? J. Cancer Res. Clin. Oncol. 145, 3047–3054. 10.1007/s00432-019-03022-x 31506741 PMC11810182

[B124] Thomas M. F. L'Etoile N. D. Ansel K. M. (2014). Eri1: a conserved enzyme at the crossroads of multiple RNA-processing pathways. Trends Genet. 30, 298–307. 10.1016/j.tig.2014.05.003 24929628 PMC4114243

[B125] Thorsson V. Gibbs D. L. Brown S. D. Wolf D. Bortone D. S. Yang T.-H. O.. (2018). The immune landscape of cancer. Immunity. 48, 812–830.e14. 10.1016/j.immuni.2018.03.023 29628290 PMC5982584

[B126] Trapnell C. Cacchiarelli D. Grimsby J. Pokharel P. Li S. Morse M.. (2014). The dynamics and regulators of cell fate decisions are revealed by pseudotemporal ordering of single cells. Nat. Biotechnol. 32, 381–386. 10.1038/nbt.2859 24658644 PMC4122333

[B127] Tsao C.-C. Baumann J. Huang S.-F. Kindler D. Schroeter A. Kachappilly N.. (2021). Pericyte hypoxia-inducible factor-1 (HIF-1) drives blood-brain barrier disruption and impacts acute ischemic stroke outcome. Angiogenesis 24, 823–842. 10.1007/s10456-021-09796-4 34046769 PMC8487886

[B128] Tsuyama J. Nakamura A. Ooboshi H. Yoshimura A. Shichita T. (2018). Pivotal role of innate myeloid cells in cerebral post-ischemic sterile inflammation. Semin. Immunopathol. 40, 523–538. 10.1007/s00281-018-0707-8 30206661

[B129] Umphlett M. Shea S. Tome-Garcia J. Zhang Y. Hormigo A. Fowkes M.. (2020). Widely metastatic glioblastoma with BRCA1 and ARID1A mutations: a case report. BMC Cancer 20, 47. 10.1186/s12885-020-6540-1 31959133 PMC6971940

[B130] Uneda A. Kurozumi K. Fujimura A. Fujii K. Ishida J. Shimazu Y.. (2021). Differentiated glioblastoma cells accelerate tumor progression by shaping the tumor microenvironment via CCN1-mediated macrophage infiltration. Acta Neuropathol Commun. 9, 29. 10.1186/s40478-021-01124-7 33618763 PMC7898455

[B131] Van Nostrand E. L. Freese P. Pratt G. A. Wang X. Wei X. Xiao R.. (2020). A large-scale binding and functional map of human RNA-binding proteins. Nature 583, 711–719. 10.1038/s41586-020-2077-3 32728246 PMC7410833

[B132] Velasco M. X. Kosti A. Penalva L. O. F. Hernández G. (2019). The diverse roles of RNA-binding proteins in glioma development. Adv. Exp. Med. Biol. 1157, 29–39. 10.1007/978-3-030-19966-1_2 31342436

[B133] Vidal R. Wagner J. U. G. Braeuning C. Fischer C. Patrick R. Tombor L.. (2019). Transcriptional heterogeneity of fibroblasts is a hallmark of the aging heart. JCI Insight 4, 131092. 10.1172/jci.insight.131092 31723062 PMC6948853

[B134] Villanueva E. Smith T. Queiroz R. M. L. Monti M. Pizzinga M. Elzek M.. (2020). Efficient recovery of the RNA-bound proteome and protein-bound transcriptome using phase separation (OOPS). Nat. Protoc. 15, 2568–2588. 10.1038/s41596-020-0344-2 32651564 PMC7613161

[B135] Wang E. Lu S. X. Pastore A. Chen X. Imig J. Chun-Wei Lee S.. (2019). Targeting an RNA-binding protein network in acute myeloid leukemia. Cancer Cell 35, 369–384.e7. 10.1016/j.ccell.2019.01.010 30799057 PMC6424627

[B136] Wang J. Qi J. Hou X. (2019). Systematically dissecting the function of RNA-binding proteins during glioma progression. Front. Genet. 10, 1394. 10.3389/fgene.2019.01394 32047515 PMC6997557

[B137] Wang Z. Tang W. Yuan J. Qiang B. Han W. Peng X.. (2020). Integrated analysis of RNA-binding proteins in glioma. Cancers (Basel) 12, E892. 10.3390/cancers12040892 32272554 PMC7226056

[B138] Williamson M. R. Fuertes C. J. A. Dunn A. K. Drew M. R. Jones T. A. (2021). Reactive astrocytes facilitate vascular repair and remodeling after stroke. Cell Rep. 35, 109048. 10.1016/j.celrep.2021.109048 33910014 PMC8142687

[B139] Winkler E. A. Bell R. D. Zlokovic B. V. (2011). Central nervous system pericytes in health and disease. Nat. Neurosci. 14, 1398–1405. 10.1038/nn.2946 22030551 PMC4020628

[B140] Wojtasiewicz T. J. Ducruet A. F. Noticewala S. S. Canoll P. McKhann G. M. (2013). De novo glioblastoma in the territory of a prior middle cerebral artery infarct. Case Rep. Neurol. Med. 2013, 1–5. 10.1155/2013/356526 24222871 PMC3810319

[B141] Xu H. Jiang Y. Xu X. Su X. Liu Y. Ma Y.. (2019). Inducible degradation of lncRNA Sros1 promotes IFN-γ-mediated activation of innate immune responses by stabilizing Stat1 mRNA. Nat. Immunol. 20, 1621–1630. 10.1038/s41590-019-0542-7 31740800

[B142] Xu P. Liu Q. Xie Y. Shi X. Li Y. Peng M.. (2018). Breast cancer susceptibility protein 1 (BRCA1) rescues neurons from cerebral ischemia/reperfusion injury through NRF2-mediated antioxidant pathway. Redox Biol. 18, 158–172. 10.1016/j.redox.2018.06.012 30014904 PMC6068089

[B143] Xu P. Shi X. Zhang X. Liu Q. Xie Y. Hong Y.. (2019). Overexpression of BRCA1 in neural stem cells enhances cell survival and functional recovery after transplantation into experimental ischemic stroke. Oxid. Med. Cell. Longev. 2019, 8739730. 10.1155/2019/8739730 31073355 PMC6470423

[B144] Xu S. Lu J. Shao A. Zhang J. H. Zhang J. (2020). Glial cells: role of the immune response in ischemic stroke. Front. Immunol. 11, 294. 10.3389/fimmu.2020.00294 32174916 PMC7055422

[B145] Xu S. Tang L. Dai G. Luo C. Liu Z. (2021). Immune-related genes with APA in microenvironment indicate risk stratification and clinical prognosis in grade II/III gliomas. Mol. Therap. Nucl. Acids 23, 1229–1242. 10.1016/j.omtn.2021.01.033 33665000 PMC7900014

[B146] Yang Y. Yan R. Zhang L. Meng X. Sun W. (2020). Primary glioblastoma transcriptome data analysis for screening survival-related genes. J. Cell. Biochem. 121, 1901–1910. 10.1002/jcb.29425 31633244

[B147] Yoshihara K. Shahmoradgoli M. Martínez E. Vegesna R. Kim H. Torres-Garcia W.. (2013). Inferring tumour purity and stromal and immune cell admixture from expression data. Nat. Commun. 4, 2612. 10.1038/ncomms3612 24113773 PMC3826632

[B148] Zhai Y. Zhao B. Wang Y. Li L. Li J. Li X.. (2021). Construction of the optimization prognostic model based on differentially expressed immune genes of lung adenocarcinoma. BMC Cancer 21, 213. 10.1186/s12885-021-07911-8 33648465 PMC7923649

[B149] Zhang H. Meltzer P. Davis S. (2013). RCircos: an R package for Circos 2D track plots. BMC Bioinform. 14, 244. 10.1186/1471-2105-14-244 23937229 PMC3765848

[B150] Zhang Z. Guo M. Liu Y. Liu P. Cao X. Xu Y.. (2020). RNPS1 inhibition aggravates ischemic brain injury and promotes neuronal death. Biochem. Biophys. Res. Commun. 523, 39–45. 10.1016/j.bbrc.2019.11.185 31831174

[B151] Zhao S.-C. Wang C. Xu H. Wu W.-Q. Chu Z.-H. Ma L.-S.. (2017). Age-related differences in interferon regulatory factor-4 and−5 signaling in ischemic brains of mice. Acta Pharmacol. Sin. 38, 1425–1434. 10.1038/aps.2017.122 28905935 PMC5672072

[B152] Zhao Z. Wang Y. Yun D. Huang Q. Meng D. Li Q.. (2020). TRIM21 overexpression promotes tumor progression by regulating cell proliferation, cell migration and cell senescence in human glioma. Am. J. Cancer Res. 10, 114–130. 32064156 PMC7017742

[B153] Zhou M. Yang W.-L. Ji Y. Qiang X. Wang P. (2014). Cold-inducible RNA-binding protein mediates neuroinflammation in cerebral ischemia. Biochim. Biophys. Acta General Subjects 1840, 2253–2261. 10.1016/j.bbagen.2014.02.027 24613680 PMC4061249

