# Supplementary material for: Identification of a 6-RBP gene signature for a comprehensive analysis of glioma and ischemic stroke: Cognitive impairment and aging-related hypoxic stress
Source: Front Aging Neurosci. 2022 Sep 1;14:951197. doi: 10.3389/fnagi.2022.951197 (PMC9476601; doi:10.3389/fnagi.2022.951197)
Supplement: Supplementary file 1 [file Data_Sheet_1.pdf]

Supplementary Figures

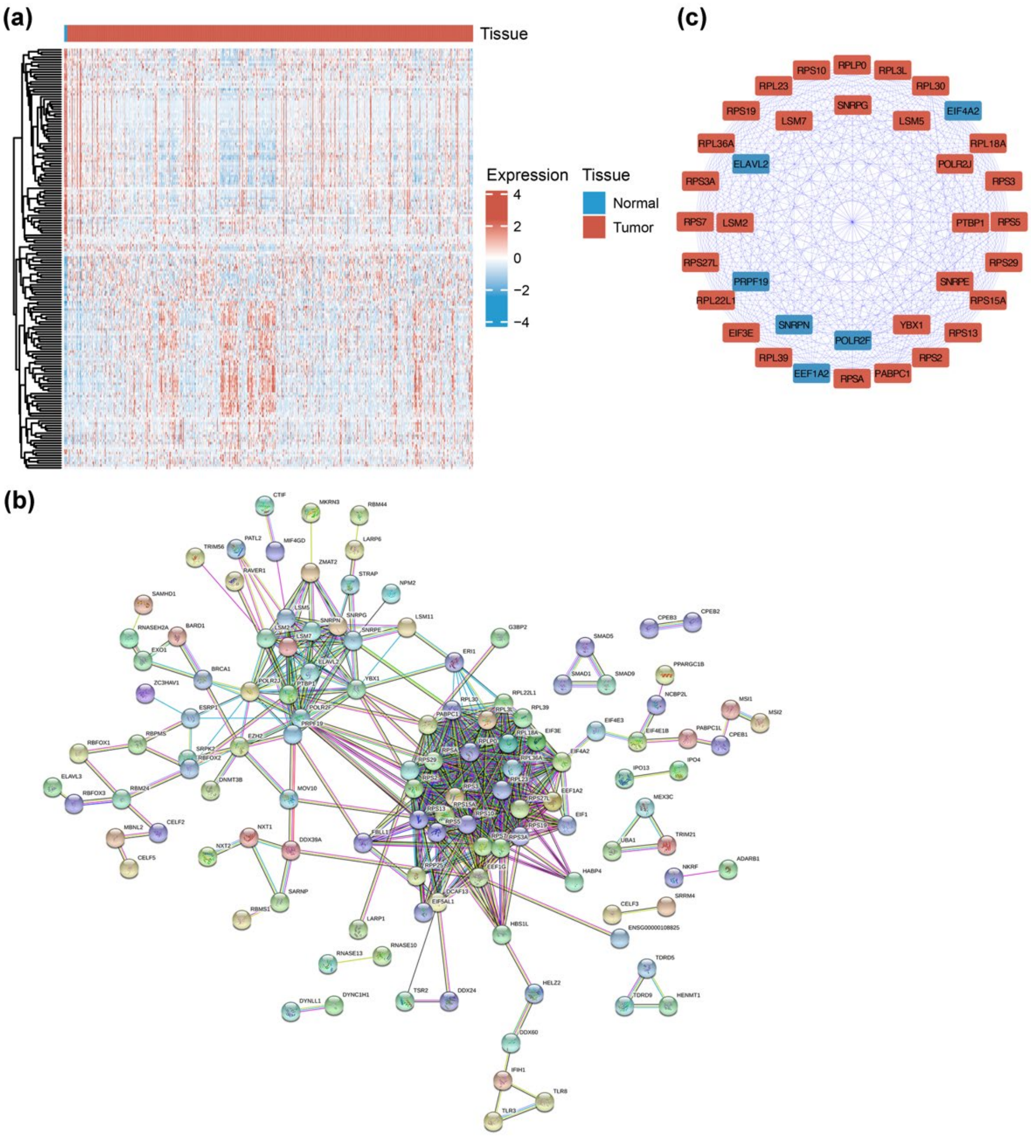

Supplementary Figure 1. Heatmap and Protein-protein interaction analysis of DE RBP genes. (a) Heatmap of the 170 differentially expressed RBP genes between the glioma and normal samples. (b) The STRING analysis of protein-protein interaction networks. (c) Analysis of PPI subnetworks using Cytoscape software.

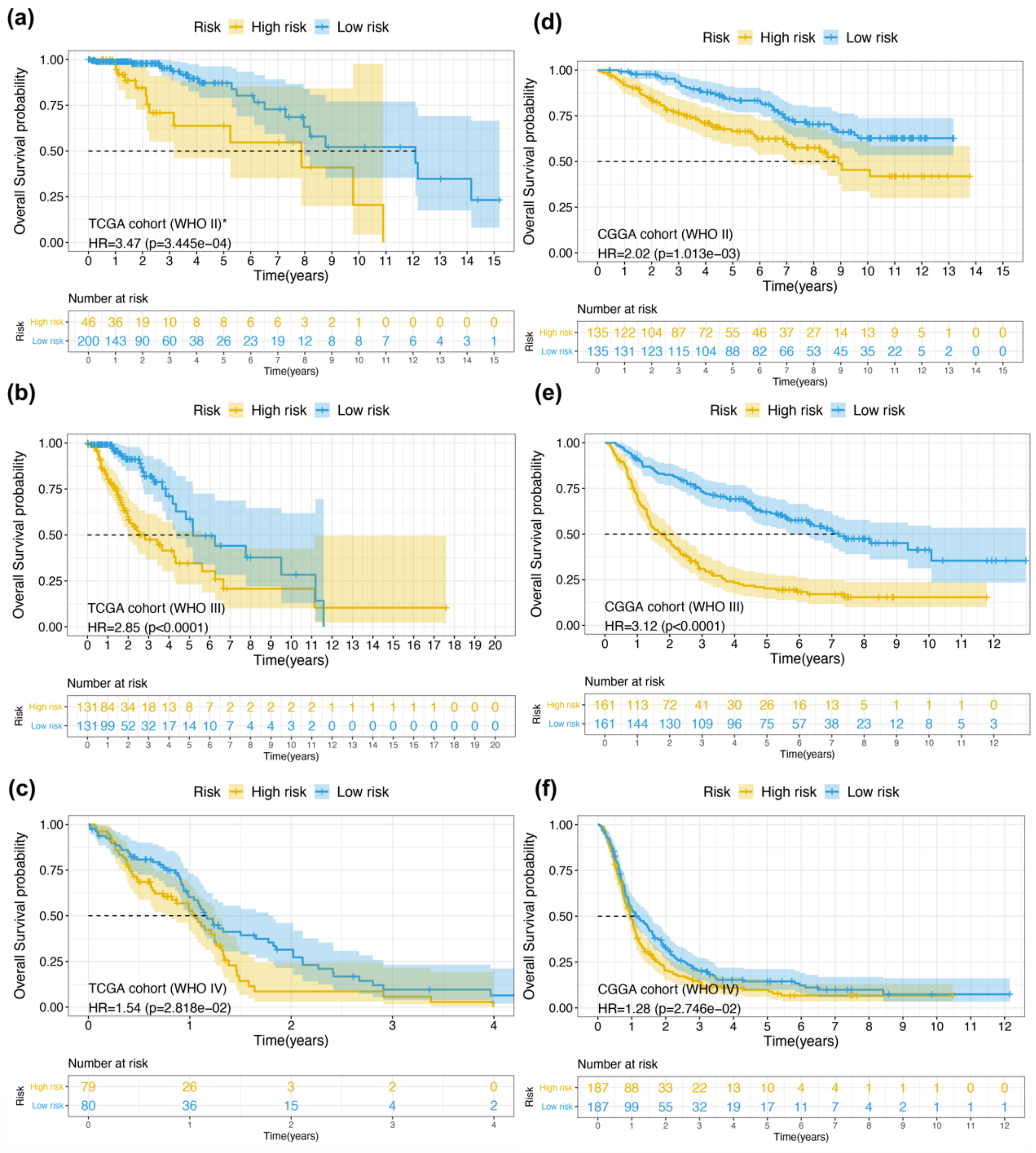

Supplementary Figure 2. Survival analysis of patients with glioma between the high- and low-risk groups by TCGA and CGGA database. Yellow indicated high risk and blue indicated low risk in glioma for WHO grade II, WHO grade III, and WHO grade IV ((a)–(c)) by TCGA database and ((d)–(f)) the CGGA database.

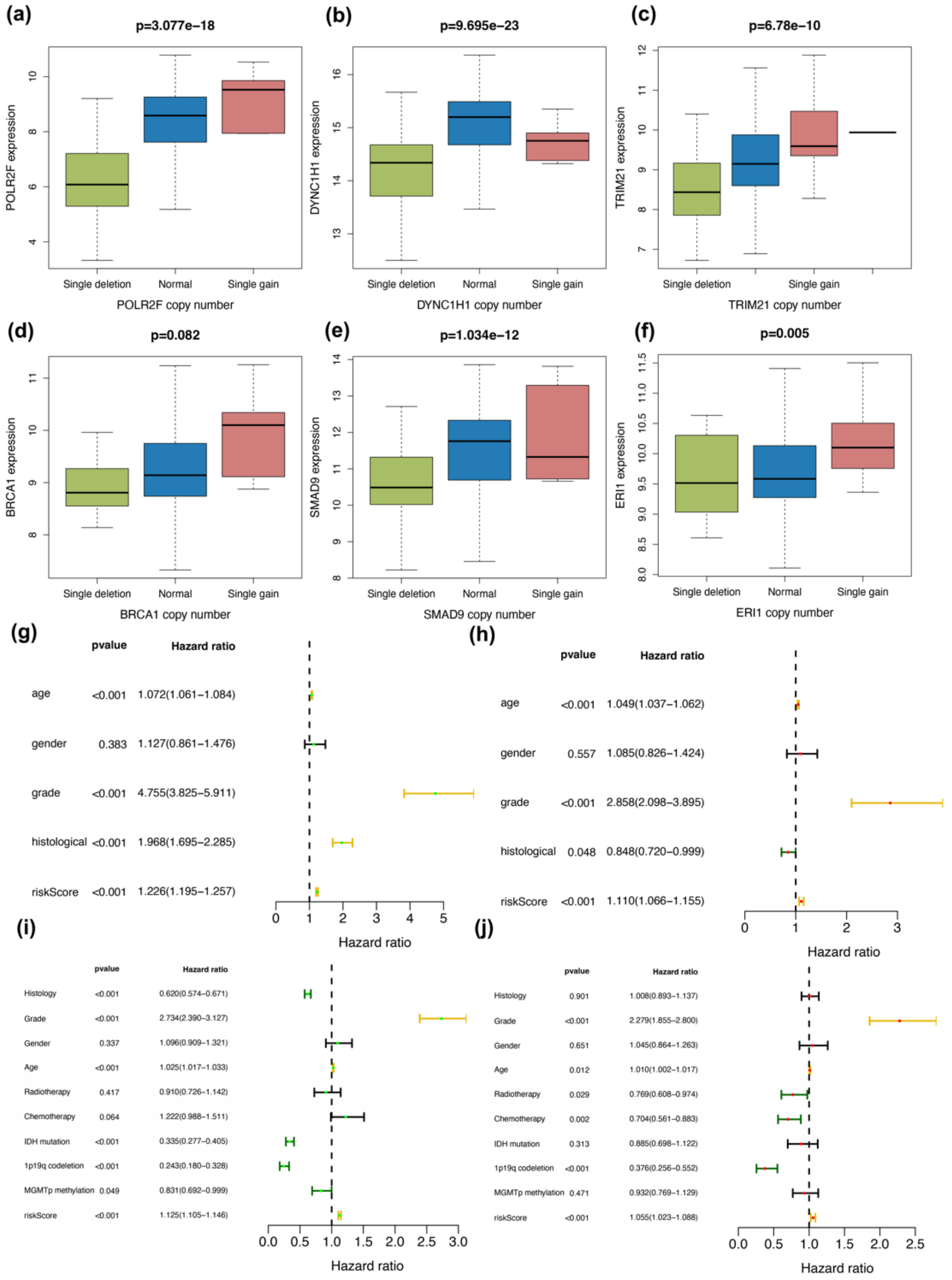

Supplementary Figure 3. Analysis of the six RBP genes ((a)–(f)) expression between their copy number variants, and cox analysis of overall survival based on the 6-gene signature and clinical characteristics. (g) Univariate and (h) multivariate Cox analysis of the 6-gene signature in the TCGA database. (i) Univariate and (j) multivariate Cox analysis of the 6-gene signature in the CGGA database.

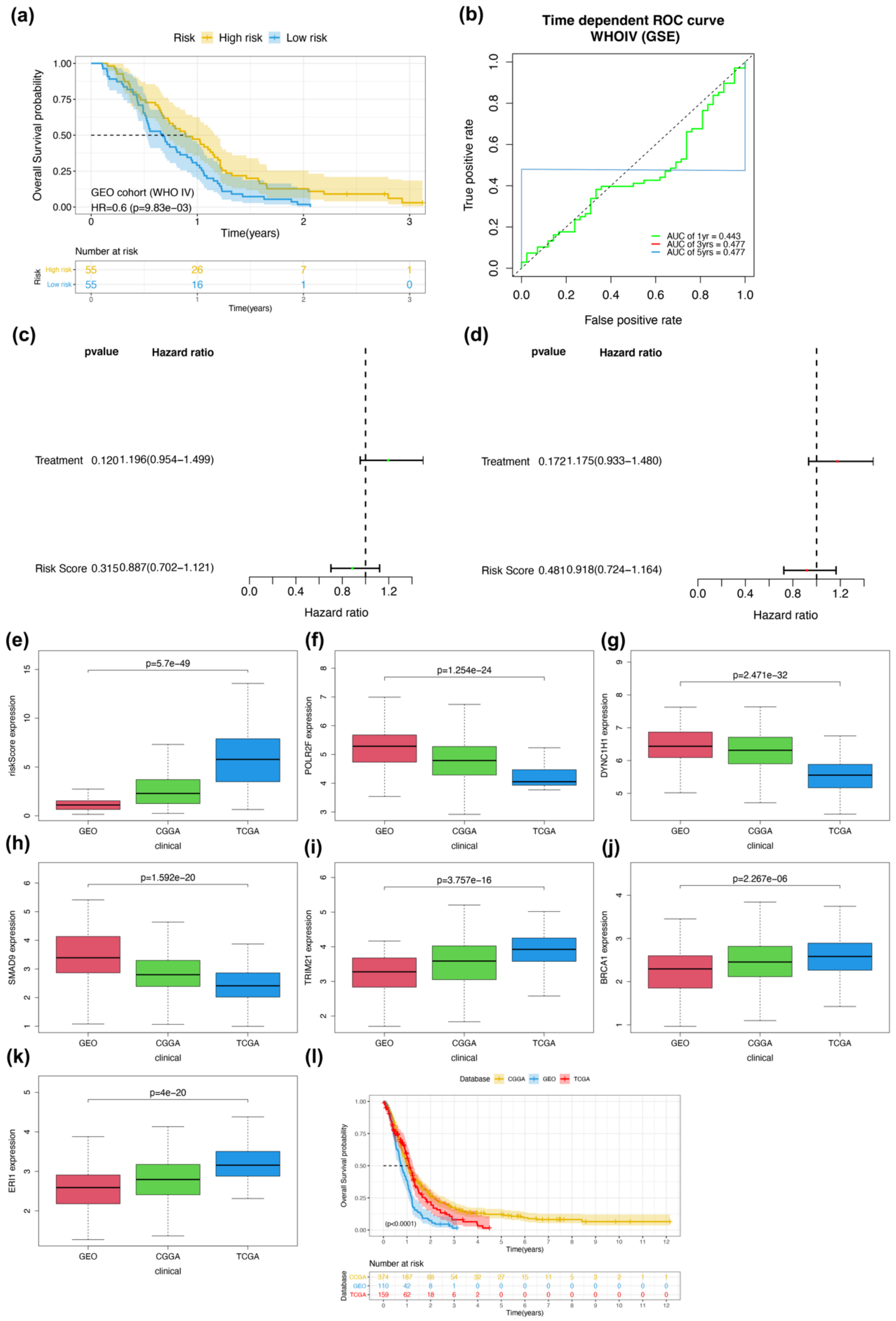

Supplementary Figure 4. Bioinformatics analysis of the 6-gene risk stratification signature in the GSE72951 database. (a) Survival analysis (KM method) of patients

with grade IV glioma showed a significant difference between the high- and low-risk groups ($P = 0.010$). (b) Receiver operator characteristic curve analysis of the 6 RBPS. (c) Univariate and (d) multivariate Cox analysis of the RBPS and treatment showed that neither treatment nor RBPS was associated with survival in the GSE72951 dataset. The risk score of the RBPS (e), the expression of the three protective genes ((f)–(h)), and the expression of the deleterious genes ((i)–(k)) were significantly different among the three datasets. (l) The survival curves plot showed a statistically significant difference in the median overall survival time between the three datasets, with the GSE72951 dataset having the worst OS.

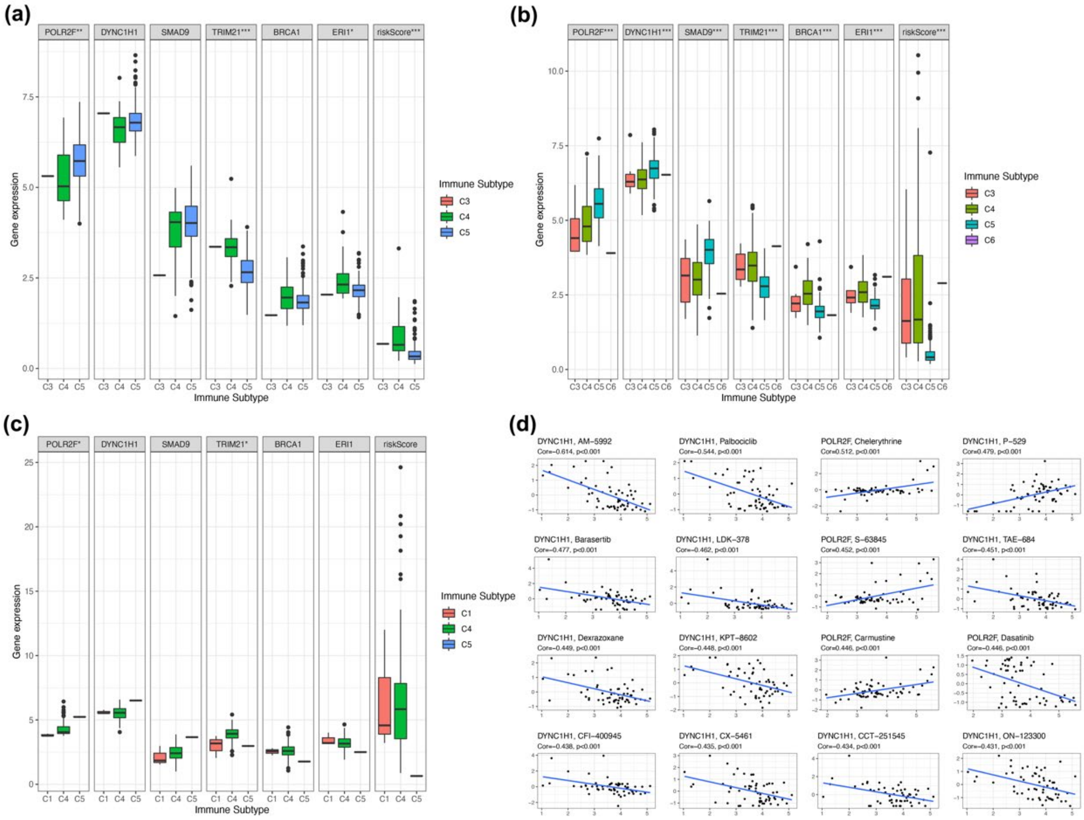

Supplementary Figure 5. Relationships between the expression of the 6 RBP genes (*POLR2F*, *DYNC1H1*, *SMAD9*, *TRIM21*, *BRCA1*, *ERI1*) and immune subtypes in grade (a) II, (b) III, and (c) IV glioma. (d) Relationships between the six RBP genes (*POLR2F*,

DYNC1H1, *SMAD9*, *TRIM21*, *BRCA1*, *ERI1*) and potential drugs (top 16 sorted by *P*-value).

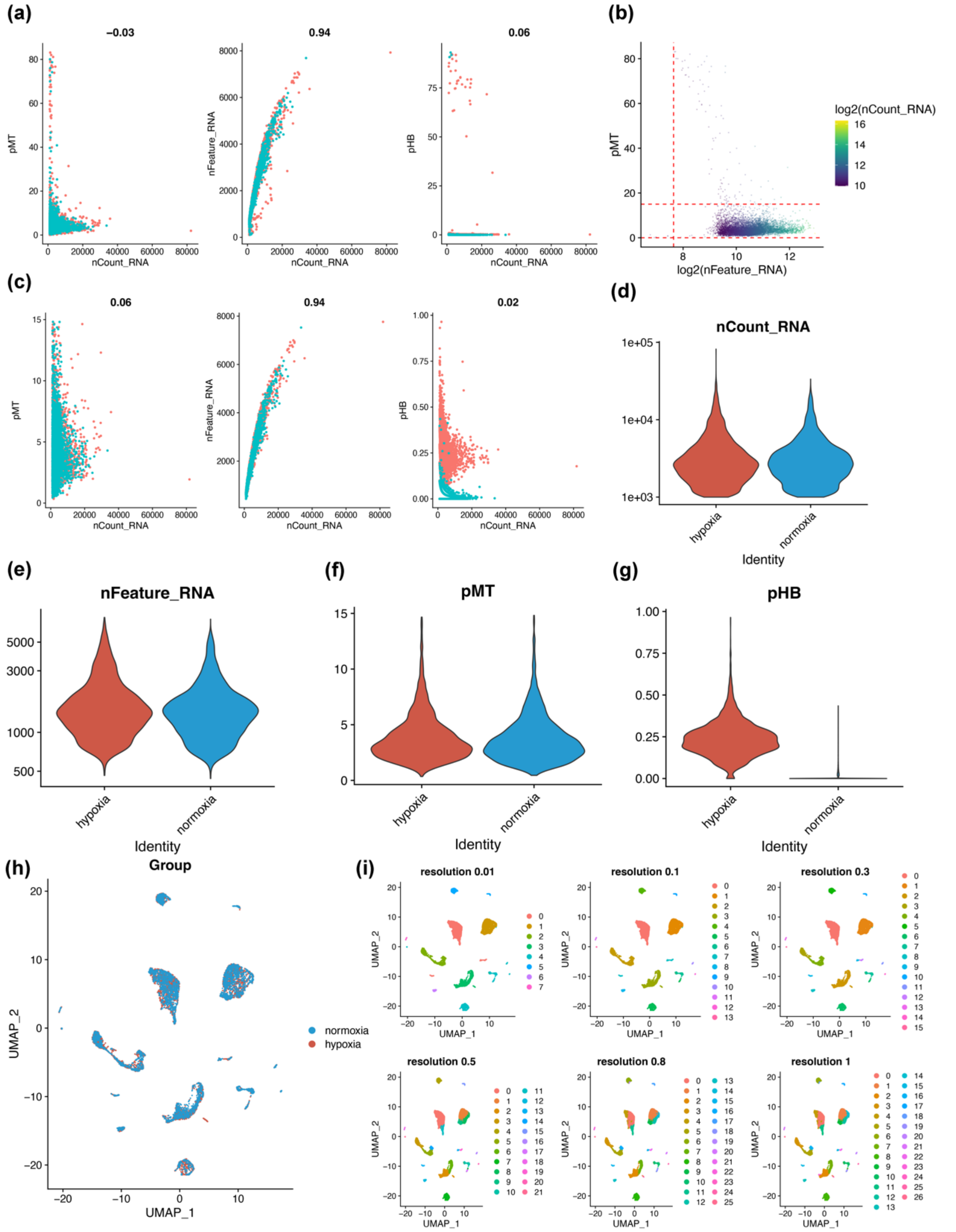

Supplementary Figure 6. Cell quality control in single cell analysis. (A) Distribution of cell characteristics before data quality filtering. (B) Conditions of cell filtration. (c)-

(g) Distribution of cellular characteristics after data quality filtering. (H) UMAP shows the batch effect between samples after data consolidation. (i) The results of cell clustering at different resolution values are displayed.

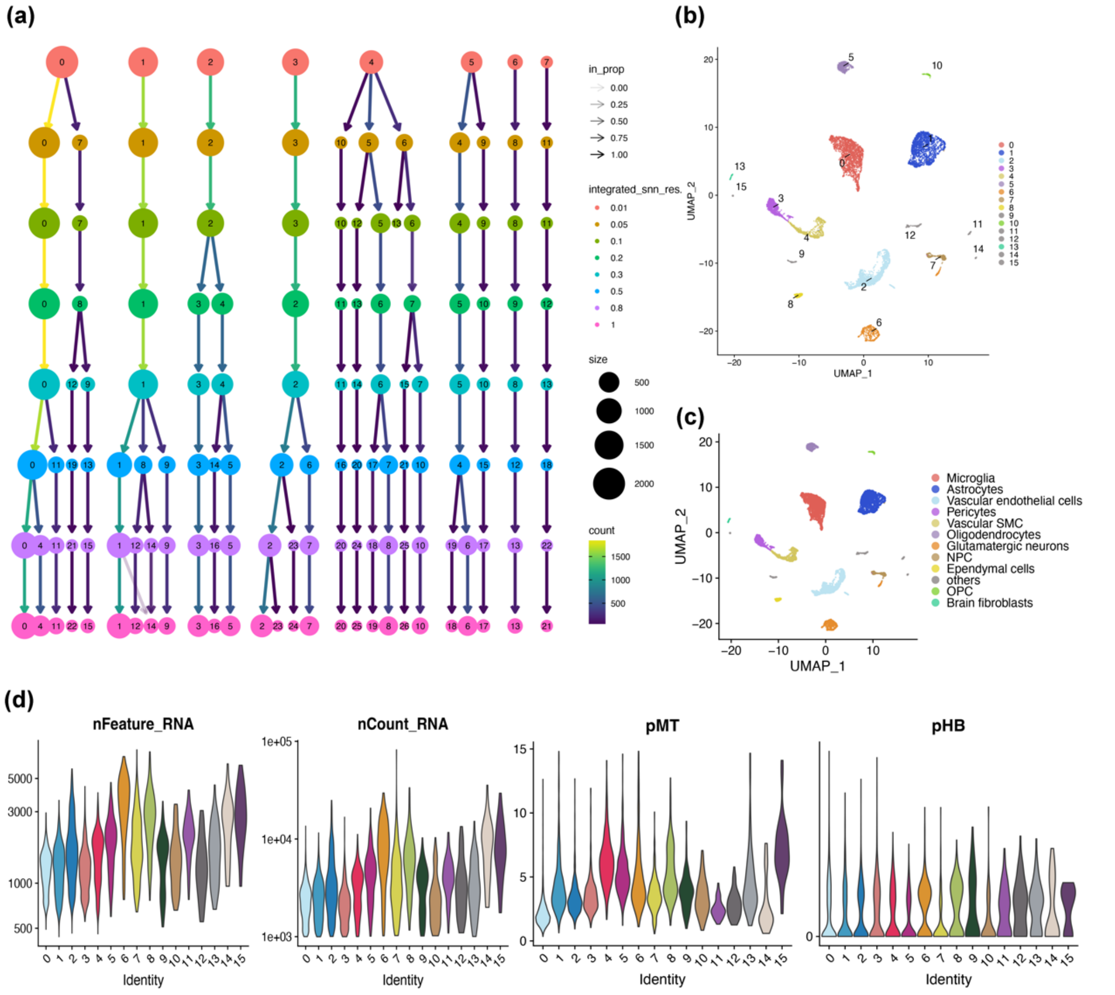

Supplementary Figure 7. Cell clustering process. (A) Cell clustering process under different solution values. (B) UMAP showed cell clustering results. (c) Cell annotation results. (D) The characteristic distribution of cells in these 16 cell clusters was displayed.

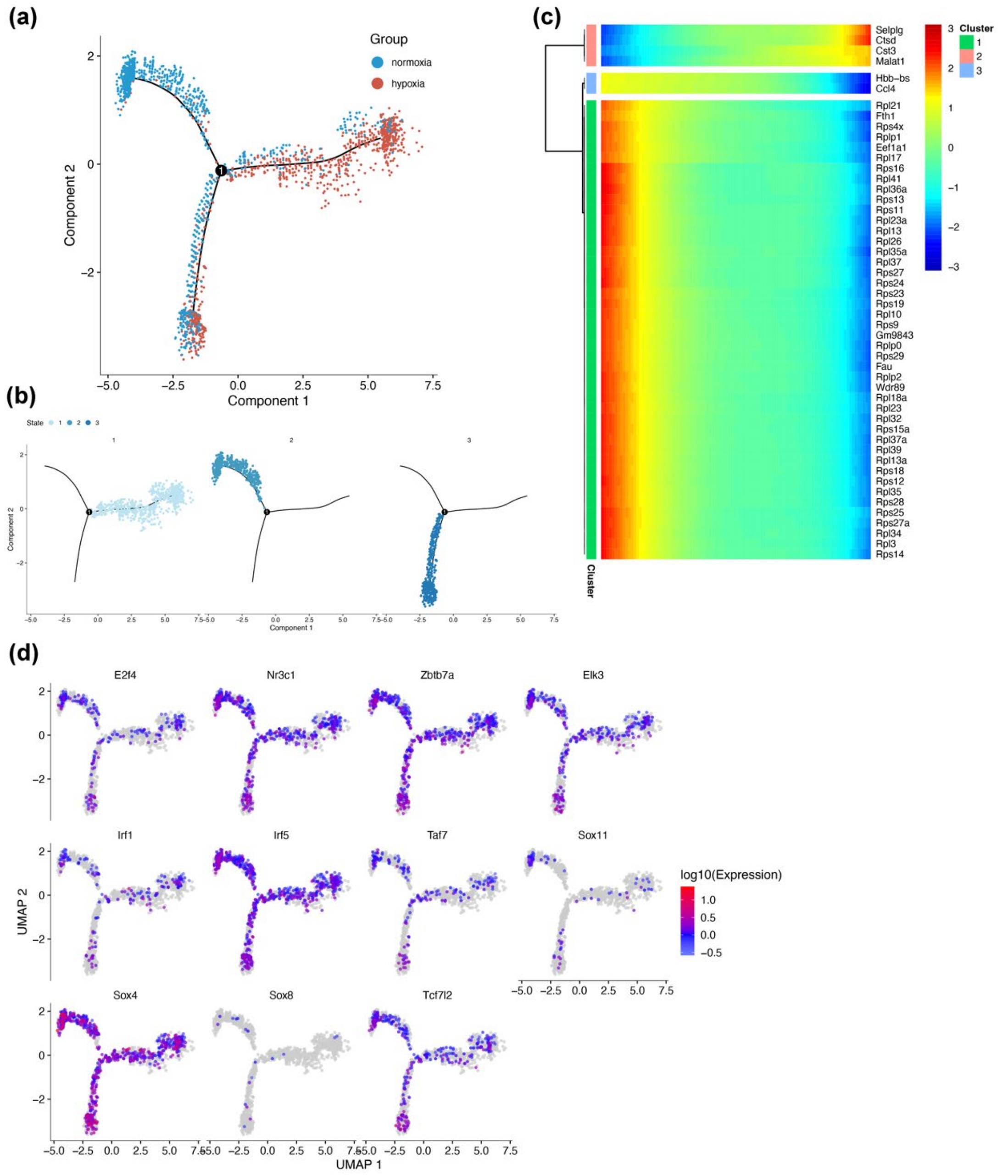

Supplementary Figure 8. Pseudo-time sequence analysis of microglia. (a) Show the corresponding distribution of normal oxygen group and hypoxia group in pseudo-time distribution map. (b) The quasi-time series results show three stages. (c) The top 50 DEGs most relevant to quasi-time series. (d) The distribution of RBP-related transcription factors in the pseudo-sequence diagram.

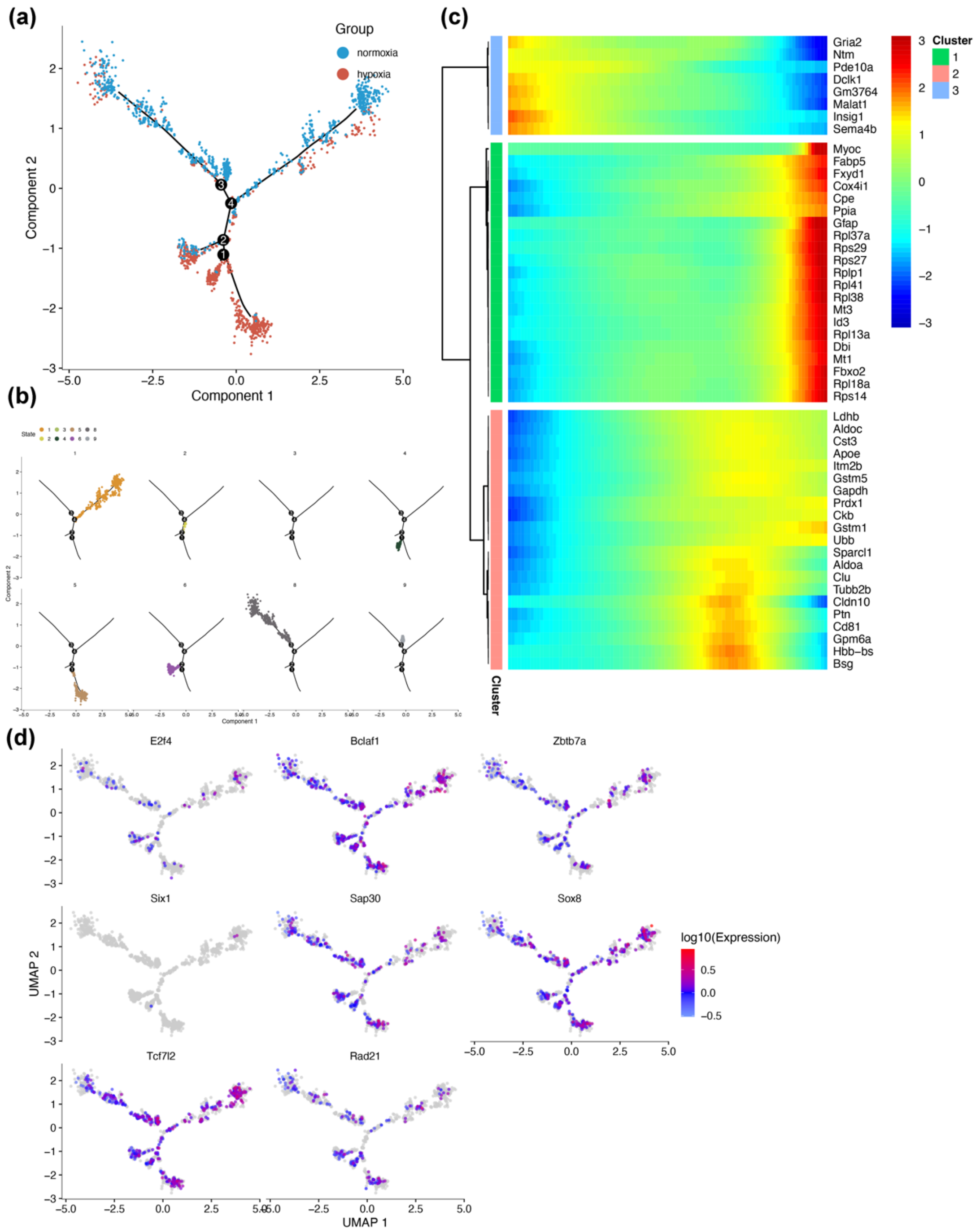

Supplementary Figure 9. Pseudo-temporal distribution of astrocytes. (a) To show the corresponding distribution of astrocytes in the pseudo-temporal distribution map in normal oxygen group and hypoxia group. (b) The quasi-time series results show three stages. (c) The top 50 DEGs most relevant to quasi-time series. (d) The distribution of RBP-related transcription factors in the pseudo-sequence diagram.

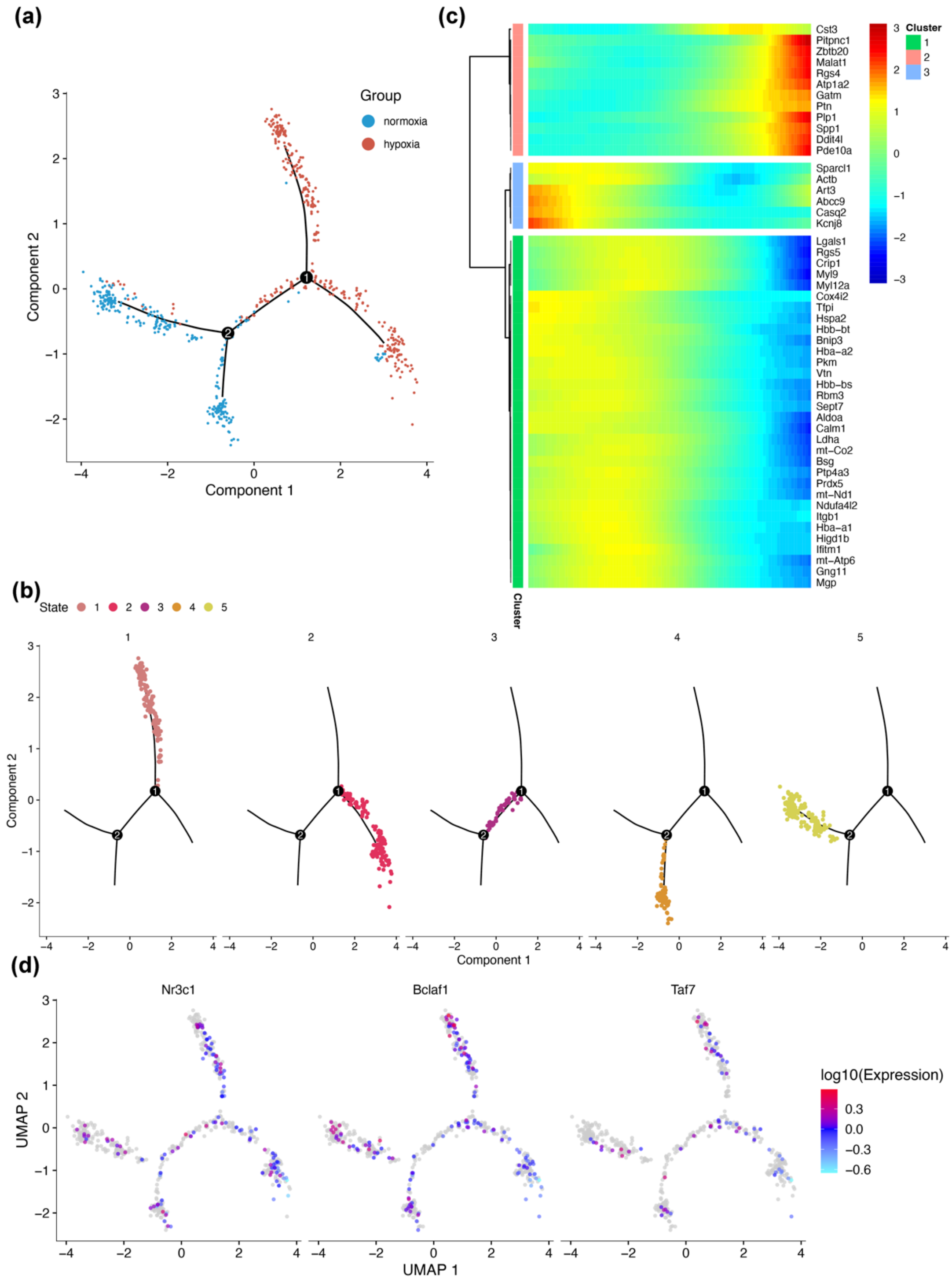

Supplementary Figure 10. Pseudo-temporal distribution of pericytes. (a) To show the corresponding distribution of pericytes in the pseudo-temporal distribution map in normal oxygen group and hypoxia group. (b) The quasi-time series results show three

stages. (c) The top 50 DEGs most relevant to quasi-time series. (d) The distribution of RBP-related transcription factors in the pseudo-sequence diagram.

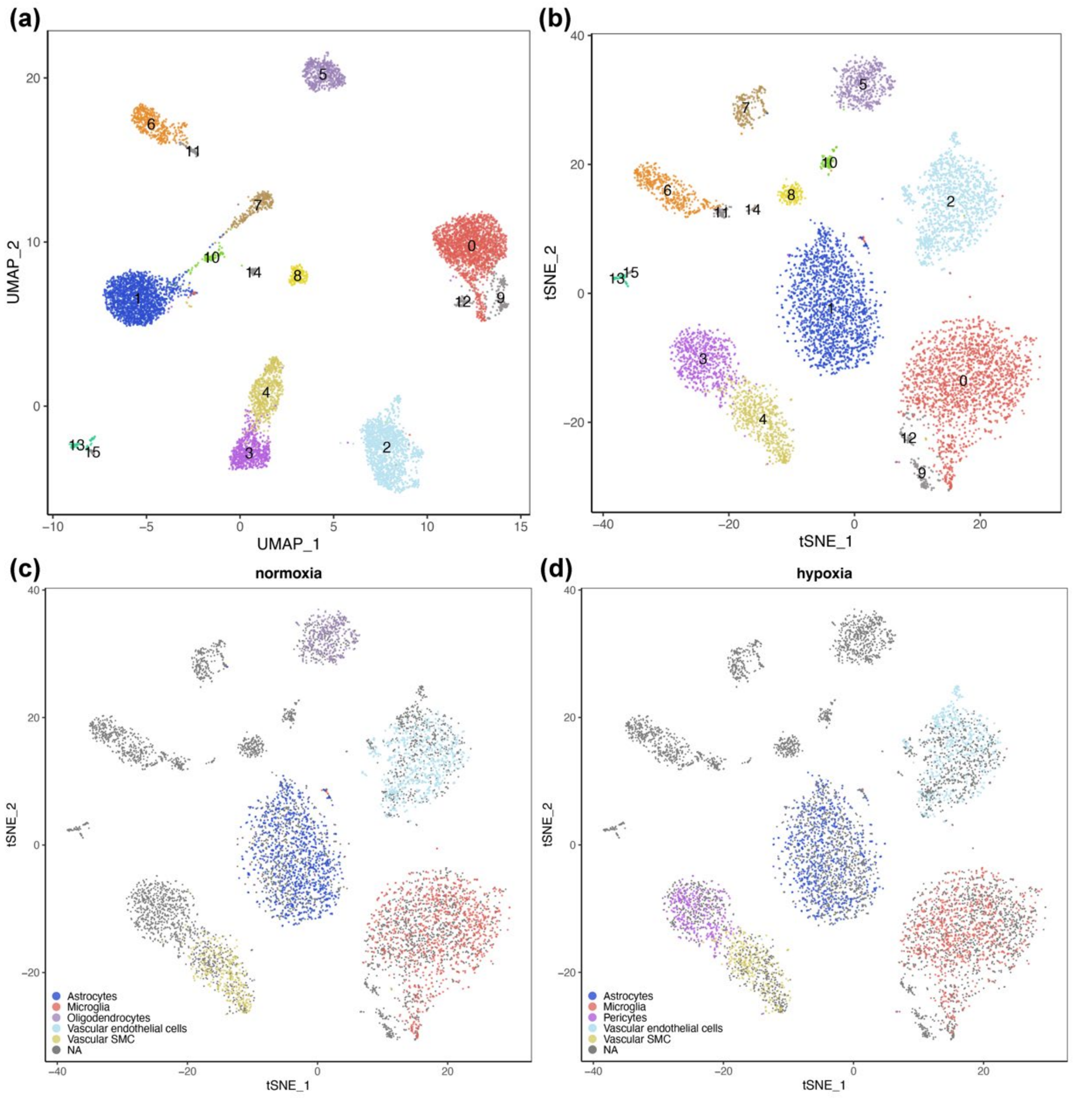

Supplementary Figure 11. Cell clustering results in SCENIC analysis. (A) The distribution of cells after clustering was displayed by UMAP. (b) The distribution of cells after clustering was displayed by tSNE. (c) Distribution of cell types in the normal oxygen concentration group. (D) Distribution of various cell types in the hypoxic group.

Supplementary Tables

Supplementary Table 1. Results of multivariate Cox model.

Gene	Coef	HR	HR.95L	HR.95H	*P* value
POLR2F	-0.313	0.731	0.567	0.943	0.016
DYNC1H1	-0.303	0.738	0.576	0.946	0.016
SMAD9	-0.432	0.649	0.539	0.783	0.000
TRIM21	0.294	1.342	1.051	1.713	0.018
BRCA1	0.525	1.690	1.297	2.203	<0.001
ERI1	0.400	1.491	1.068	2.082	0.019

Supplementary Table 2. Training model and comparison results of 10 TPOT models.

Model	training process		training set							
	Average CV score	Test Accuracy	Sensitivity	Specificity	Accuracy	Kappa Score	Hamming Loss	AUC	Average Precision	Precision
1	0.83	0.73	0.96	0.91	0.94	0.88	0.06	0.98	0.99	0.93
2	0.87	0.76	0.96	0.96	0.96	0.92	0.04	1.00	1.00	0.96
3	0.87	0.67	0.98	0.96	0.97	0.94	0.03	1.00	1.00	0.96
4	0.83	0.70	1.00	1.00	1.00	1.00	0.00	1.00	1.00	1.00
5	0.86	0.82	1.00	1.00	1.00	1.00	0.00	1.00	1.00	1.00
6	0.86	0.70	1.00	1.00	1.00	1.00	0.00	1.00	1.00	1.00
7	0.86	0.76	0.81	0.78	0.80	0.59	0.20	0.86	0.87	0.81
8	0.84	0.73	1.00	1.00	1.00	1.00	0.00	1.00	1.00	1.00
9	0.85	0.79	1.00	1.00	1.00	1.00	0.00	1.00	1.00	1.00
10	0.83	0.73	0.94	0.78	0.87	0.73	0.13	0.96	0.96	0.83

Supplementary Table 3. Testing model and comparison results of 10 TPOT models.

Model	testing set							
	Sensitivity	Specificity	Accuracy	Kappa Score	Hamming Loss	AUC	Average Precision	Precision
1	0.85	0.65	0.73	0.46	0.27	0.87	0.85	0.61
2	0.85	0.70	0.76	0.52	0.24	0.83	0.77	0.65
3	0.77	0.60	0.67	0.35	0.33	0.82	0.82	0.56
4	0.77	0.65	0.70	0.40	0.30	0.88	0.86	0.59
5	0.85	0.80	0.82	0.63	0.18	0.85	0.75	0.73
6	0.62	0.75	0.70	0.37	0.30	0.82	0.76	0.62
7	0.77	0.75	0.76	0.51	0.24	0.87	0.76	0.67
8	0.85	0.65	0.73	0.46	0.27	0.84	0.79	0.61
9	0.77	0.80	0.79	0.56	0.21	0.85	0.76	0.71
10	0.85	0.65	0.73	0.46	0.27	0.88	0.87	0.61

**Supplementary Table 4. Parameters of the 10 models.**
Model [1] = make_pipeline(<br>StackingEstimator(estimator=BernoulliNB(alpha=100.0, fit_prior=True)),<br>StackingEstimator(estimator=LinearSVC(C=15.0, dual=False, loss="squared_hinge", penalty="l2", tol=0.001)),<br>ExtraTreesClassifier(bootstrap=True, criterion="gini", max_features=0.15000000000000002, min_samples_leaf=18, min_samples_split=7, n_estimators=100)<br>)
Model [2] = make_pipeline(<br>Binarizer(threshold=0.35000000000000003),<br>SelectPercentile(score_func=f_classif, percentile=31),<br>ExtraTreesClassifier(bootstrap=False, criterion="gini", max_features=0.45, min_samples_leaf=2, min_samples_split=6, n_estimators=100)<br>)
Model [3] = make_pipeline(<br>Binarizer(threshold=0.30000000000000004),<br>StackingEstimator(estimator=RandomForestClassifier(bootstrap=True, criterion="gini", max_features=0.05, min_samples_leaf=11, min_samples_split=4, n_estimators=100)),<br>OneHotEncoder(minimum_fraction=0.25, sparse=False, threshold=10),<br>ExtraTreesClassifier(bootstrap=False, criterion="gini", max_features=0.9500000000000001, min_samples_leaf=1, min_samples_split=10, n_estimators=100)<br>)
Model[4] = make_pipeline(<br>StackingEstimator(estimator=GradientBoostingClassifier(learning_rate=0.1, max_depth=8, max_features=0.9000000000000001, min_samples_leaf=1, min_samples_split=9, n_estimators=100, subsample=0.5)),<br>StackingEstimator(estimator=GradientBoostingClassifier(learning_rate=1.0, max_depth=7, max_features=0.15000000000000002, min_samples_leaf=3, min_samples_split=3, n_estimators=100, subsample=0.4)),<br>ExtraTreesClassifier(bootstrap=True, criterion="entropy", max_features=0.6000000000000001, min_samples_leaf=14, min_samples_split=16, n_estimators=100)<br>)
Model [5] = make_pipeline(

Binarizer(threshold=0.30000000000000004),<br>OneHotEncoder(minimum_fraction=0.15, sparse=False, threshold=10),<br>GradientBoostingClassifier(learning_rate=0.5, max_depth=8, max_features=0.3, min_samples_leaf=1, min_samples_split=3, n_estimators=100, subsample=0.9500000000000001)<br>)<br>Model [6] = make_pipeline(<br>make_union(<br>StackingEstimator(estimator=ExtraTreesClassifier(bootstrap=False, criterion="gini", max_features=0.3, min_samples_leaf=4, min_samples_split=12, n_estimators=100)),<br>FunctionTransformer(copy)<br>),<br>Binarizer(threshold=0.15000000000000002),<br>RandomForestClassifier(bootstrap=False, criterion="gini", max_features=0.55, min_samples_leaf=1, min_samples_split=5, n_estimators=100)<br>)
Model [7] =make_pipeline(<br>make_union(<br>FunctionTransformer(copy),<br>FunctionTransformer(copy)<br>),<br>Normalizer(norm="l2"),<br>FeatureAgglomeration(affinity="l2", linkage="complete"),<br>StackingEstimator(estimator=SGDClassifier(alpha=0.01, eta0=0.1, fit_intercept=True, l1_ratio=0.25, learning_rate="invscaling", loss="modified_huber", penalty="elasticnet", power_t=100.0)),<br>RandomForestClassifier(bootstrap=True, criterion="gini", max_features=0.7000000000000001, min_samples_leaf=13, min_samples_split=15, n_estimators=100)<br>)
Model [8] = GradientBoostingClassifier(learning_rate=0.5, max_depth=6, max_features=0.7000000000000001, min_samples_leaf=2, min_samples_split=5, n_estimators=100, subsample=0.7500000000000001)
Model [9] = make_pipeline(<br>Binarizer(threshold=0.30000000000000004),<br>StackingEstimator(estimator=GradientBoostingClassifier(learning_rate=0.001, max_depth=4, max_features=0.55, min_samples_leaf=4, min_samples_split=2, n_estimators=100, subsample=0.2)),

ExtraTreesClassifier(bootstrap=False, criterion="gini", max_features=0.25, min_samples_leaf=1, min_samples_split=5, n_estimators=100)<br>)
Model [10] = make_pipeline(<br>StackingEstimator(estimator=SGDClassifier(alpha=0.0, eta0=0.1, fit_intercept=True, l1_ratio=0.0, learning_rate="constant", loss="perceptron", penalty="elasticnet", power_t=1.0)),<br>ExtraTreesClassifier(bootstrap=True, criterion="gini", max_features=0.1, min_samples_leaf=11, min_samples_split=3, n_estimators=100)<br>)
